# Honey bee (*Apis mellifera*) wing images: a tool for identification and conservation

**DOI:** 10.1093/gigascience/giad019

**Published:** 2023-03-27

**Authors:** Andrzej Oleksa, Eliza Căuia, Adrian Siceanu, Zlatko Puškadija, Marin Kovačić, M Alice Pinto, Pedro João Rodrigues, Fani Hatjina, Leonidas Charistos, Maria Bouga, Janez Prešern, İrfan Kandemir, Slađan Rašić, Szilvia Kusza, Adam Tofilski

**Affiliations:** Department of Genetics, Faculty of Biological Sciences, Kazimierz Wielki University, Bydgoszcz 85-090, Poland; Honeybee Genetics and Breeding Laboratory, Institute for Beekeeping Research and Development, Bucharest 013975, Romania; Honeybee Genetics and Breeding Laboratory, Institute for Beekeeping Research and Development, Bucharest 013975, Romania; Faculty of Agrobiotechnical Sciences, Josip Juraj Strossmayer University of Osijek, Osijek 31000, Croatia; Faculty of Agrobiotechnical Sciences, Josip Juraj Strossmayer University of Osijek, Osijek 31000, Croatia; Centro de Investigação de Montanha, Instituto Politécnico de Bragança, Campus de Santa Apolónia, Bragança 5300-253, Portugal; Laboratório Associado para a Sustentabilidade e Tecnologia em Regiões de Montanha (SusTEC), Instituto Politécnico de Bragança, Campus de Santa Apolónia, Bragança 5300-253, Portugal; Centre in Digitalization and Intelligent Robotics, Instituto Politécnico de Bragança, Campus de Santa Apolónia, Bragança 5300-253, Portugal; Laboratório Associado para a Sustentabilidade e Tecnologia em Regiões de Montanha (SusTEC), Instituto Politécnico de Bragança, Campus de Santa Apolónia, Bragança 5300-253, Portugal; Department of Apiculture, Institute of Animal Science–Ellinikos Georgikos Organismos ‘DIMITRA’, Nea Moudania 63200, Greece; Department of Apiculture, Institute of Animal Science–Ellinikos Georgikos Organismos ‘DIMITRA’, Nea Moudania 63200, Greece; Lab of Agricultural Zoology and Entomology, Agricultural University of Athens, Athens 11855, Greece; Agricultural Institute of Slovenia, Ljubljana SI-1000, Slovenia; Ankara University, Department of Biology, Faculty of Science, Ankara University, Beşevler-Ankara 06100, Turkey; Faculty of Ecological Agriculture, EDUCONS University, Sremska Kamenica 21208, Serbia; Centre for Agricultural Genomics and Biotechnology, University of Debrecen, Debrecen 4032, Hungary; Department of Zoology and Animal Welfare, University of Agriculture in Krakow, Krakow 31-425, Poland

**Keywords:** honey bee, *Apis mellifera*, biodiversity, conservation, wing, geometric morphometrics

## Abstract

**Background:**

The honey bee (*Apis mellifera*) is an ecologically and economically important species that provides pollination services to natural and agricultural systems. The biodiversity of the honey bee in parts of its native range is endangered by migratory beekeeping and commercial breeding. In consequence, some honey bee populations that are well adapted to the local environment are threatened with extinction. A crucial step for the protection of honey bee biodiversity is reliable differentiation between native and nonnative bees. One of the methods that can be used for this is the geometric morphometrics of wings. This method is fast, is low cost, and does not require expensive equipment. Therefore, it can be easily used by both scientists and beekeepers. However, wing geometric morphometrics is challenging due to the lack of reference data that can be reliably used for comparisons between different geographic regions.

**Findings:**

Here, we provide an unprecedented collection of 26,481 honey bee wing images representing 1,725 samples from 13 European countries. The wing images are accompanied by the coordinates of 19 landmarks and the geographic coordinates of the sampling locations. We present an R script that describes the workflow for analyzing the data and identifying an unknown sample. We compared the data with available reference samples for lineage and found general agreement with them.

**Conclusions:**

The extensive collection of wing images available on the Zenodo website can be used to identify the geographic origin of unknown samples and therefore assist in the monitoring and conservation of honey bee biodiversity in Europe.

## Data Description

**Figure 1:**
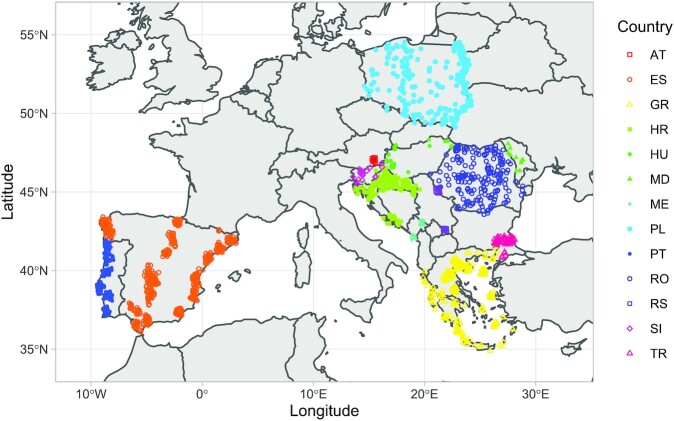
Locations from which honey bee samples were collected. Jitter was used to show multiple samples from the same or similar location.

**Figure 2:**
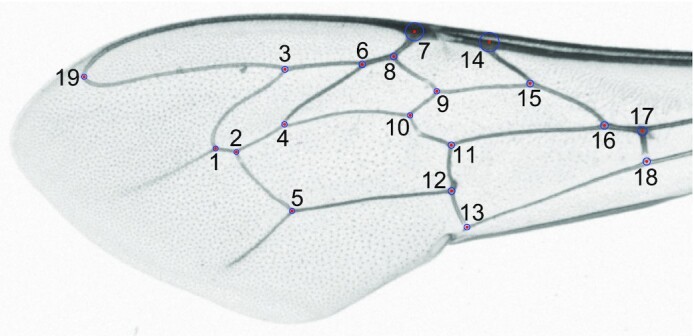
Position of landmarks on the forewing of a honey bee worker. The landmarks are indicated with red dots. The blue circles around them should be tangent to the venation outline in 3 points.

We provide 26,481 forewing images of honey bee workers. They represent 1,725 samples from 13 European countries (Table 1, Fig. 1). The shape of the wings was described using the coordinates for 19 landmarks at wing veins’ intersections (Fig. 2). The whole dataset, including the wing images, landmark coordinates, geographic coordinates of sampling locations, and other data, is available on the Zenodo website [1] under a public domain license.

**Table 1:** Sample size of honey bee wings used in this study

Country	Country abbreviation	Number of wings	Number of samples
Austria	AT	198	10
Croatia	HR	6,103	160
Greece	GR	1,444	244
Hungary	HU	426	22
Moldova	MD	263	10
Montenegro	ME	300	20
Poland	PL	5,955	253
Portugal	PT	960	192
Romania	RO	6,498	197
Serbia	RS	299	20
Slovenia	SI	835	21
Spain	ES	2,563	516
Turkey	TR	637	60

## Introduction

Honey bees (*Apis mellifera*, NCBI:txid7460) are ecologically and economically important. Their value as pollinators of wild plants and crops is much greater than the economical return from honey or other products of the beekeeping industry [2]. At the same time, in the United States [3] and some European countries [4, 5] there has been a decline in the number of managed honey bee colonies. In addition to pathogens, pesticides [6], and socioeconomic factors [7], loss of genetic variability is considered one of the possible causes of such decline [8–10].

The honey bee native distribution covers Europe, up to a latitude of about 60 degrees north, Africa, the Middle East [11], and Central Asia [12, 13]. Within this wide range, the environment varies markedly. Such diverse environmental conditions, as well as the history of range expansion and isolation of populations, have resulted in notable variation of morphologic and behavioral traits, as represented by more than 24 subspecies (geographical races) [11–18]. The subspecies were initially grouped into 4 evolutionary lineages (A, C, M, and O [11]) from morphologic data, but recently, 3 additional lineages (Y, L, and U) were identified from molecular data [19, 20].

The biodiversity of the honey bee is becoming increasingly endangered by the mass introduction of queens produced by breeding. This process began in the 19th century [21] and has intensified recently [22, 23]. The honey bee queens used by many beekeepers are the daughters of relatively few selected breeder queens [24], which are often hybrids [25]. In some European countries, more than 15% of all colonies are re-queened every year [26]. In consequence, some populations, which are well adapted to the local environment [27], are threatened by introgressive hybridization [22]. In this context, it is important to distinguish native honey bees, which occurred in a particular area before the intensification of beekeeping, from nonnative ones, introduced into the area by human intervention.

The conservation of honey bee subspecies requires their identification, which may be based on molecular markers. Recently, identification methods have been developed from single-nucleotide polymorphisms (SNPs) [28–30], which are more accurate than microsatellites [31]. The problem is that, despite the decreasing costs of SNP genotyping [29], molecular identification is still expensive and not easily and quickly accessible to beekeepers. A cheaper alternative is the identification of subspecies based on wing venation measurements [11, 18, 32]. The wings can be measured using various methodologies based on distances and angles [33], landmark coordinates [34–36], outlines [37], or image pixels [38]. While it has been demonstrated that morphologic and molecular markers might provide similar results [39, 40, but see also 41], identification always requires a reference dataset, which is often inadequate.

Public data suitable for the identification of honey bee subspecies or evolutionary lineages using wing morphometry are scarce. Early studies based on multiple measurements of wings provided averages and standard deviations for all measured distances and angles, as well as details related to linear discriminant analysis (LDA) [42, 43]. Unfortunately, in later studies, including those covering global honey bee diversity, averages were not provided [33] or were provided only for a few selected variables [11]. For example, in a study of *Apis mellifera mellifera*, only 9 out of 36 characteristics were reported [11: Table 13.1]; among them, there are no data for the venation angles used for discrimination of this subspecies [11: p. 229]. More important, the details of LDA were often not presented in those studies, preventing readers from using them to identify unknown samples. Later, the LDA details were provided in a few studies [34, 36, 44–46]. However, despite this progress, it would be even more useful to give readers access to all of the raw data used in the analysis instead of providing LDA details, as has been done in some studies related to wing measurements in Diptera [47–49]. In the case of honey bees, only 1 study is known to us in which the landmark data were made available [50]. Providing landmark coordinates solves the problem of data availability only partially because different studies may use different configurations of landmarks [18]. Not only can the order of landmarks differ between studies, but their positions may also vary. This makes comparisons between studies difficult or even impossible. On the other hand, if a wing image is available, it can be reanalyzed, and missing or incompatible landmarks can be determined. In addition, the wing images can be used to determine the landmarks automatically [35, 51, 52].

Currently, while wing measurements are usually based on images, they are rarely made available after publication. This applies not only to honey bees but also to other insects. Usually, only 1 image is presented with information about the position of the landmarks [47]. There are only a few examples in which wing images have been made publicly available. This is the case of 1 study on Vespidae [53] and 1 study on *Drosophila melanogaster*, for which a large repository of wing images was recently provided [54]. Honey bee wing images have never been made available in such significant numbers.

The lack of reference data on the morphologic variation of honey bees can be alleviated by data sharing, which is a common practice in some scientific fields, including genomics [55] and neurosciences [56]. It is one of the factors that has facilitated the rapid growth of those fields in recent years. In contrast to molecular biology, data sharing is relatively rare in ecological studies [57]. The benefits of data sharing within the scientific community are well known. However, individual authors often resist making their data available [58–60]. Even if some data are provided, they are often incomplete [61]. Large-scale investigations require large datasets [62], which are difficult for a single researcher to obtain. Individual studies on honey bee biogeography often focus on a relatively small area of 1 or a few countries, and large-scale comparisons are rare [32, 39, 45]. Data sharing would allow the combining of datasets from multiple studies to obtain better knowledge about large-scale geographic variation and the conservation status of honey bee subspecies.

In an attempt to begin building a global reference dataset for the honey bee, here we provide an extensive repository of wing images representative of its diversity in a large tract of Europe. The wing images are accompanied by the coordinates of 19 landmarks, which can easily be used in future comparisons. We also present an R script in which we analyze the coordinates and show how they can be used to identify the origin of an unknown sample. Among other applications, the repository can be used to identify native honey bees, which is essential for their conservation.

## Methods

### Material

In this study, we used 26,481 forewing images of honey bee workers (nonreproductive diploid females). They represent 1,725 samples from 13 European countries (Table 1, Fig. 1). A sample consists of workers that were collected either from 1 colony or from flowers in 1 location. In the case of samples collected from colonies, the workers were swept with a brush from a middle comb. In the case of samples collected from flowers, the workers were captured using an entomological net. After collection, the workers were stored in alcohol until their preparation. The number of workers per sample ranged from 5 to 20. In some cases (Poland and Hungary), when only 1 or 2 workers were collected from 1 location, neighboring locations were treated together to obtain at least 10 wings per sample. The samples from Austria, Montenegro, and Serbia were obtained from queen breeders who use artificial selection. While breeding lines in Austria were maintained through instrumental insemination of queens, queen bees in Montenegro and Serbia were open-mated. Country names are abbreviated according to ISO 3166–1 (Table 1). The geographic coordinates of the samples, the year of their collection, and other information are provided in CSV files for each country separately.

Some of the samples were analyzed in earlier studies addressing other goals: Croatia and Slovenia [63], Greece [64, 65], Hungary and Poland in part [66, 67], Portugal and Spain [39, 41], Romania [68], Serbia and Montenegro [69], and Turkey [70]. Those studies used various methods, and their results could not be directly compared. Therefore, most of the wings were remeasured for the analyses performed herein. In none of the earlier studies were the wing images made publicly available. The data from Austria and Moldova had never been analyzed or published before. Wing preparation and image acquisition differed between the studies. In most cases, the wings were detached from the bees’ bodies and then mounted between 2 microscopic slides. For image acquisition, different types of cameras combined with stereo microscopes [39, 64, 65, 69, 70] or macro lenses [63, 66–68, 68] were used.

Each wing from the dataset was saved as a separate PNG file. The wing image file name begins with the 2-letter country code (Table 1), followed by a hyphen (identical to the minus sign), a 4-digit sample code, another hyphen, and finally the original file name. The original file names vary as they originate from various studies. They usually consist of strings separated by hyphens. In some cases, the name ends with a letter L or R indicating left or right wing. The samples were independently numbered in each country; for this reason, the unique sample name has to include the 2-letter country code. The wing images were sorted by country and compressed into 13 ZIP files.

In each wing image, the coordinates of 19 landmarks were determined (Fig. 2) [45]. The landmarks are compatible with the “standard honey bee morphometry” approach used in earlier studies [33], and the landmark coordinates can be converted to distances and angles [as in 63]. The landmarks were saved within each wing image file and can be viewed and edited in the IdentiFly software application [45]. The raw coordinates of the landmarks were saved in CSV files for each country separately. The whole dataset, including the 26,481 forewing images, landmark coordinates, geographic coordinates of sampling locations, and other data, is available on the Zenodo website [1] under a public domain license.

### Statistical analysis

The statistical analysis was performed in R (v. 4.0.3) [71] using RStudio (v. 2022.12.0, RRID:SCR_000432). All details of the statistical analysis are available at the WorkflowHub website [72]. Landmark coordinates from all wings were superimposed using generalized Procrustes analysis in the geomorph package (v. 4.0.4) [73]. The aligned coordinates were averaged within samples, and the averages were used in the subsequent analysis. Principal component analysis was used to extract the first 2 principal components, which were used to describe how the wing shape varied geographically. The association between the principal components and geographic coordinates (latitude and longitude) was analyzed using generalized additive model (GAM) regression in the mgcv package (v. 1.8–33) [74]. Canonical variate analysis (CVA) and the differences between countries and regions were calculated using the Morpho package (v. 2.9) [75]. The wing shape was clustered using the unweighted pair group method with arithmetic mean (UPGMA) in the phangorn package (v. 2.5.5) [76]. The differences in wing shape between countries or regions were described using Mahalanobis distance. The correlation between the Mahalanobis distances and geographical distances was analyzed using the Mantel test. The coordinates of the unknown samples obtained from Nawrocka et al. [45, 77] were aligned using generalized Procrustes analysis and averaged within samples. Next, the samples were aligned with a consensus of the reference sample using ordinary Procrustes analysis in the shapes package (v. 1.2.6) [78]. Finally, the CVA scores of unknown samples were obtained and compared with the CVA scores of reference samples to calculate their probabilities of belonging to each country or region.

## Results

### Exploratory data analysis

**Figure 3:**
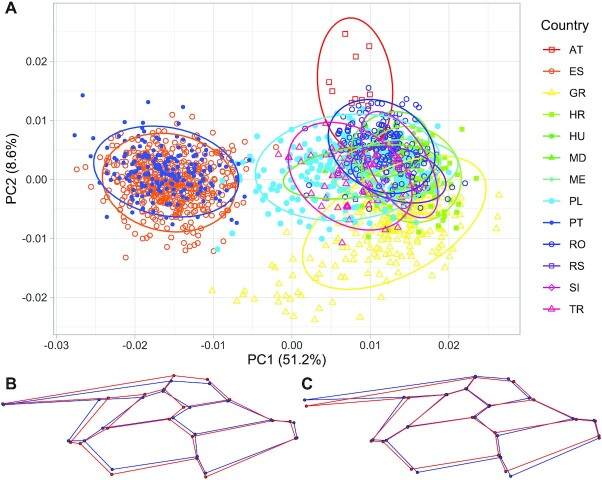
The first 2 principal components of wing shape (A). Ellipses indicate 95% confidence regions assuming multivariate *t*-distribution. The 2 bottom wireframe graphs illustrate change of wing shape along the first (B) and the second (C) principal components. The blue and red lines and dots indicate samples with minimum and maximum values, respectively.

**Figure 4:**
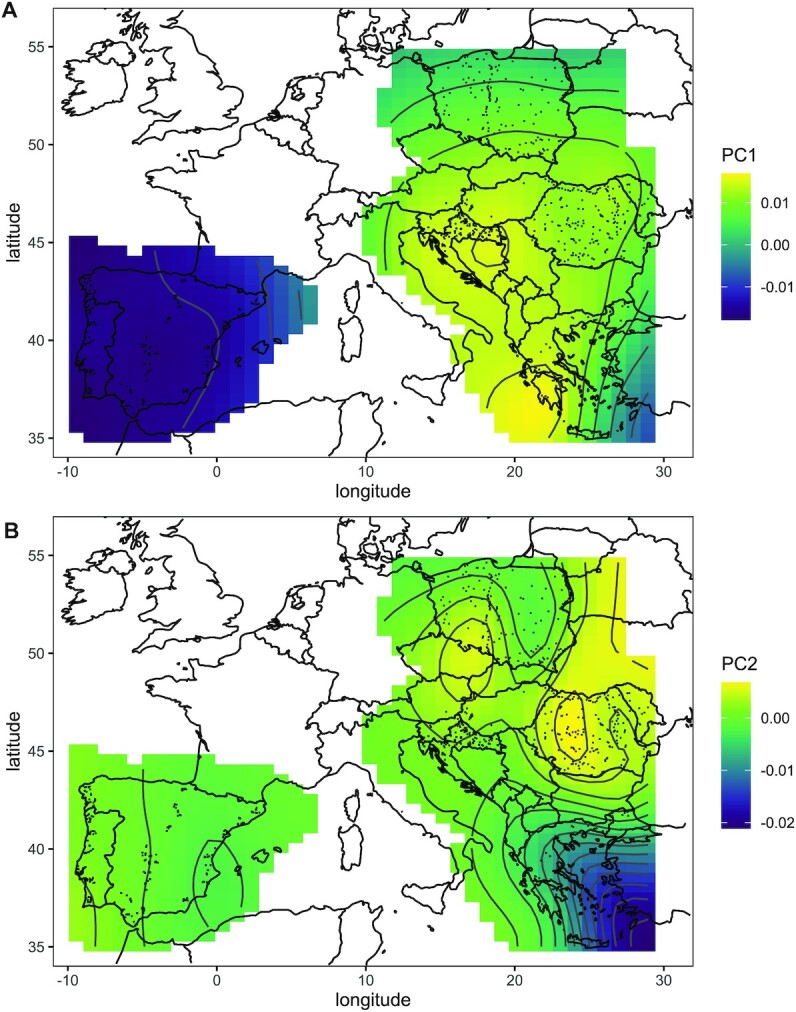
First (A) and second (B) principal components interpolated over sampling locations using a generalized additive model.

The wing shape varied significantly according to principal component analysis. In the graph of the first 2 principal components (PCs), which account for 51.2% and 8.6% of the variance, respectively, at least 2 clear clusters of points are visible (Fig. 3). One of the clusters represents the Iberian Peninsula, and the other represents central and southeastern Europe. The second principal component differentiated Greece from the countries of central Europe, particularly Austria. The wing shape variation was strongly correlated with geographic location. Latitude and longitude correlated significantly with both the first and second principal components (GAM regression, PC1: the effective degrees of freedom (EDF) = 26.18, *F* = 720.7, *P* < 10^−15^; PC2: EDF = 27.86, F = 58.47, *P* < 10^−15^). The first principal component was much lower in the Iberian Peninsula than in the Balkans (Fig. 4A). Additionally, it decreased in Poland from south to north and in Greece from west to east (Fig. 4A). The second principal component was lowest in southeastern Greece, increasing toward the north and west, with some intricate patterns in Romania and Poland (Fig. 4B).

**Figure 5:**
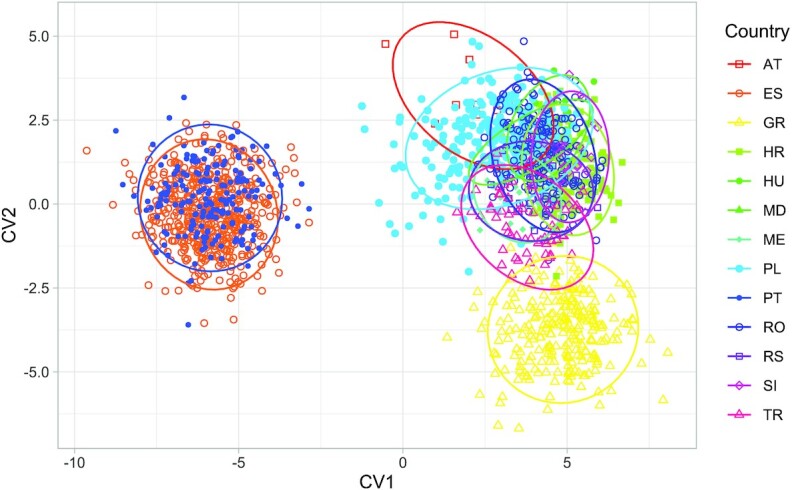
Discrimination between countries based on the first 2 canonical variates. Ellipses indicate 95% confidence regions assuming multivariate *t*-distribution.

**Figure 6:**
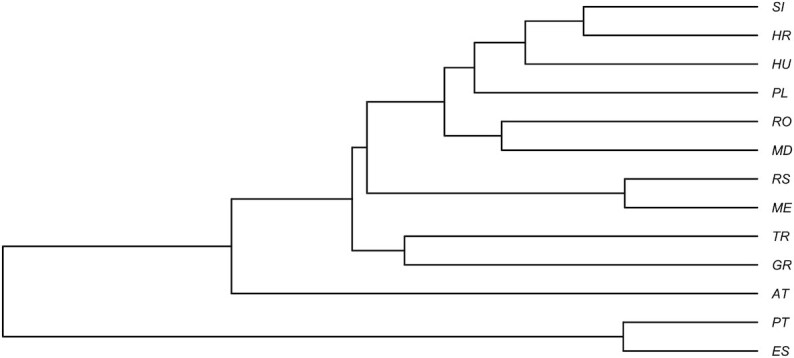
UPGMA tree illustrating similarities between the shape of the wings collected from different countries.

**Figure 7:**
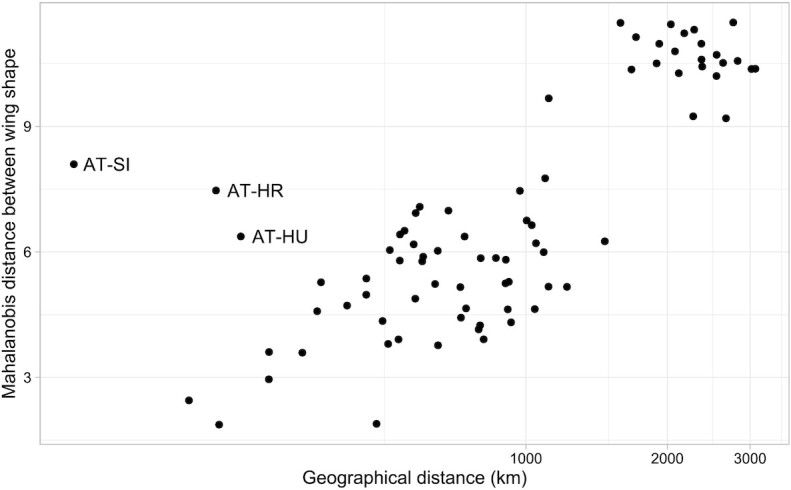
Relationship between geographical distance and Mahalanobis distance among countries.

As expected, canonical variate analysis revealed deeper differences between countries and showed a similar pattern to principal component analysis (Fig. 5). The shape of honey bee wings (represented by 34 principal components) differed significantly among countries (multivariate analysis of variance: *F* = 22.1, *P* < 10^−15^). In pairwise comparisons, most countries differed markedly from each other. Only Romania did not differ significantly from Moldova, Serbia from Montenegro, and Slovenia from Croatia and Hungary (Table 2). The largest Mahalanobis distance was found between samples from Portugal and Greece and the smallest between samples from Portugal and Spain. The UPGMA tree shows more details about the similarities between the wings collected from different countries (Fig. 6). Most neighboring countries cluster together: Portugal with Spain, Greece with Turkey, Moldova with Romania, and Slovenia with Croatia. Isolation by distance was confirmed by a significant positive correlation between geographic distances and Mahalanobis distances of wing shape between countries (Mantel test: *r* = 0.7046, *P* = 0.0015). The Austrian samples did not fit well into this relationship. Despite their close geographic proximity to Slovenia, Croatia, and Hungary, they had unexpectedly different wing shapes (Fig. 7).

**Table 2:** Differences between countries in wing shape (expressed as Mahalanobis distances, lower triangle) and significance of pairwise comparisons (upper triangle). For country abbreviations, see Table 1.

Country	AT	ES	GR	HR	HU	MD	ME	PL	PT	RO	RS	SI	TR
AT	—	0.0001	0.0001	0.0001	0.0026	0.0009	0.0002	0.0001	0.0001	0.0001	0.0005	0.0001	0.0001
ES	10.3602	—	0.0001	0.0001	0.0001	0.0001	0.0001	0.0001	0.0001	0.0001	0.0001	0.0001	0.0001
GR	9.6709	11.3111	—	0.0001	0.0001	0.0001	0.0001	0.0001	0.0001	0.0001	0.0001	0.0001	0.0001
HR	7.4676	11.1342	5.2491	—	0.0250	0.0083	0.0001	0.0001	0.0001	0.0001	0.0001	0.0933	0.0001
HU	6.3694	10.9745	6.6387	2.9550	—	0.0155	0.0003	0.0011	0.0001	0.0025	0.0017	0.0524	0.0002
MD	7.4600	10.5178	6.7506	4.6274	5.1587	—	0.0059	0.0072	0.0001	0.0669	0.0160	0.0404	0.0012
ME	6.9289	10.5056	6.4158	5.2746	5.7927	5.8540	—	0.0001	0.0001	0.0001	0.8670	0.0001	0.0001
PL	6.9864	9.2408	6.2533	3.9108	3.9095	4.6505	6.2075	—	0.0001	0.0001	0.0001	0.0004	0.0001
PT	10.2711	1.8918	11.4833	11.2249	10.9754	10.3770	10.4285	9.1929	—	0.0001	0.0001	0.0001	0.0001
RO	6.3672	10.5980	5.8505	3.7678	3.8011	3.6076	4.8826	4.4288	10.5648	—	0.0003	0.0003	0.0001
RS	7.0790	10.7933	6.1816	4.7181	5.3638	5.2334	1.8720	5.8124	10.7111	4.5827	—	0.0002	0.0001
SI	8.0962	11.4743	5.9944	2.4516	3.5920	4.6343	6.5067	4.1484	11.4397	4.2440	5.8863	—	0.0001
TR	7.7594	10.2036	4.9768	4.3165	5.2857	5.7750	6.0269	5.1654	10.3717	4.3490	6.0440	5.1704	—

### Classification of samples as lineages

**Figure 8:**
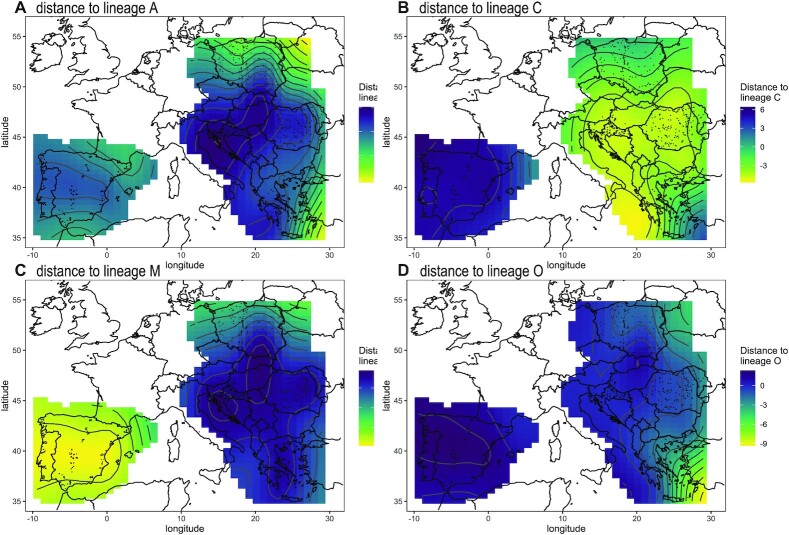
Mahalanobis distance to lineages A (A), C (B), M (C), and O (D) interpolated over sampling locations using a generalized additive model.

When the samples were classified as lineages (Supplementary Figs. S1, S2), using the data from Nawrocka et al. [45], many of them (*n* = 844, 48.9%) were classified as lineage C, which occurred in all samples from 6 countries: Austria, Croatia, Hungary, Montenegro, Serbia, and Slovenia. Lineage C was also the most frequent one in Greece, Moldova, Poland, Romania, and Turkey (Supplementary Fig. S2). As expected, the samples most similar to lineage C occurred in southeastern Europe, except in southeastern Greece (Fig. 8B).

The second most frequent was lineage M. It occurred in 652 samples (37.8%). It was dominant in Portugal and Spain but also occurred in Poland (Supplementary Fig. S2). A clear similarity to lineage M was observed in the Iberian Peninsula. Moreover, similarity to this lineage increased in Poland from south to north (Fig. 8C).

Unexpectedly, a relatively large fraction of the samples (*n* = 179, 10.4%) was classified as lineage A. It was detected mainly in Poland but also in the Iberian Peninsula, Greece, Turkey, and Moldova at lower proportions (Supplementary Fig. S2). Similarity to lineage A increased in Poland from south to north and in Greece from northwest to southeast (Fig. 8A).

Lineage O was the least frequent (*n* = 50, 2.9%). It occurred in Greece and sporadically in Moldova, Poland, Romania, and the European part of Turkey (Supplementary Fig. S2). Similarity to lineage O increased in Greece from the northwest to the southeast (Fig. 8D).

### Identification of unknown samples

The data provided here can be used for the identification of an unknown sample of honey bee workers. To test the accuracy of such identification, the leave-one-out cross-validation approach was used. This was based on samples and not on single wing measurements, in order to increase accuracy (for more information, see the Discussion). One sample was temporally removed from the dataset, which was subject to canonical variate analysis, and the obtained data were used to classify the newly removed sample. In this procedure, the removed sample was treated as unknown and the remaining data as reference. The cross-validation was repeated for all samples, and the percentage of correctly classified samples was calculated. When the samples were classified (with cross-validation) according to their country of origin, 86.26% of them were assigned to the correct group. Misclassifications most often occurred between neighboring countries. For example, the correct classification rate for Portugal was only 79.69%, with all the cases of misclassification occurring with the neighboring Spain. Many misclassifications can be attributed to the small sample size for some countries; therefore, the second classification was based on regions. Samples from some smaller countries were combined with those from their large neighbors (Portugal with Spain, Moldova with Romania, and Slovenia with Croatia), and other countries with sample sizes below 25 were excluded (Austria, Hungary, Montenegro, and Serbia). In the case of classification to regions, the correct classification rate (with cross-validation) increased to 98.31%.

Additionally, we classified to regions an independent dataset of historical data from Nawrocka et al. [45, 77]. In the analysis, we used a subset consisting of 53 colonies originating from Europe (lineages C and M). Within this subset, there was 18 colonies that originated from countries represented in the regions’ reference data. Among them, a single sample from Croatia was correctly classified as belonging to region HR-SI; out of 11 samples from Greece, 3 were correctly classified, and 8 were classified as belonging to the neighboring Turkey and the region HR-SI; 2 samples from Romania were incorrectly classified as belonging to region HR-SI; 1 sample from Slovenia was correctly classified as belonging to region HR-SI, and the other was incorrectly classified as belonging to Poland; and finally, 2 samples from Spain were correctly classified.

The reference samples provided here cover only part of Europe; therefore, identification of samples from other parts of Europe and the world can give unexpected results. Often, such samples can be detected as outliers, which have low identification probabilities for all groups. Here, we used an arbitrary threshold value of 0.001; if the maximum probability of identification for a sample is lower, we assume it is an outlier. Unfortunately, many of the identified samples, which originated from countries not covered by reference data, were classified as coming from one of the regions with a relatively high probability. For example, most samples from Italy (representing *Apis mellifera ligustica*) were classified as belonging to either Poland or HR-SI with a probability above 0.001.

## Discussion

### Comparison with earlier studies

The data presented here show that the geographic variation of honey bee wing shape in Europe is still large. This variation is most likely an effect of natural selection and not of beekeepers’ mass introduction of nonnative bees. When compared with historical reference samples from the Morphometric Bee Data Bank in Oberursel [11], the bees analyzed here fit well into the pattern, which is believed to have been shaped by natural processes. In particular, there is a high similarity to lineage M in the Iberian Peninsula and in the north of Poland, and there is a high similarity to lineage C in most of central and southeastern Europe, as expected from the seminal work on honey bee taxonomy and biogeography of Ruttner [11]. In the eastern part of the Aegean Sea, there is a high similarity to lineage O, which occurred naturally in Turkey and the Middle East. In general, the distribution of the lineages presented in this study is in line with earlier studies based on morphometry [33], mitochondrial DNA [79, 80], microsatellites [80–82], and SNPs [30, 83]. On the other hand, there are some discrepancies, which are discussed below.

It can be expected that the introduction of nonnative bees will reduce geographic variation because beekeepers prefer certain honey bee subspecies (*Apis mellifera carnica, A. m. ligustica, Apis melliferacaucasia*) or their hybrids [22]. Beekeepers’ preference for a limited number of breeding lines [24] may lead to a significant homogenization of the population structure across Europe. It is worth noting, however, that despite the increased beekeeper-mediated gene flow in the past decades [22], our results indicate that native honey bee diversity is still not lost, at least in eastern and southern Europe. The population size of honey bees is very large (approximately 19 million colonies) [84]; hence, it may take many generations to change the genetic structure and thus the phenotype of the European population. Free trade in breeding material has intensified only in the past several decades; hence, homogenization of population structure may occur in the future, unless remedial measures are taken to protect local genetic variability.

Some of the results from our study do not agree with the patterns reported by Ruttner [11]. Many colonies in northern Poland and some other countries were classified as belonging to lineage A. This lineage is expected on the African continent and does not occur naturally in central and northern Europe [11]. One possible explanation for the presence of this lineage in Europe is human-induced introgression. A recent mitochondrial DNA survey in central Europe detected haplotypes of African ancestry, although with a frequency of only 1.64% [66], which is much lower than that reported here for the forewing samples from Poland classified according to Nawrocka et al. [45] as lineage A (38.3%). The high proportion of samples assigned to lineage A may also be related to the fact that hybrids between lineages are more likely to be classified as lineage A. Those hybrids have an intermediate phenotype [85], similar to the mean shape of all lineages.

While hybridization may be caused by the introduction of nonnative bees by beekeepers, hybrids between lineages can also occur naturally. Aside from the Alps, there is no physical barrier separating lineages M and C. In such a situation, a wide hybrid zone can be expected. Earlier studies reported that in Poland, there is a wide transition zone with a clinal change in both morphologic [86, 87] and molecular [66] markers. This spatial pattern was most likely due to a natural phenomenon because it was already present in the 1960s [86], when the importation and rearing of nonnative bees were less common.

Hybrids between lineages can be identified to some degree using wing measurements [41, 85]. However, this requires adequate reference samples that are not currently available. The reference sample from the Morphometric Bee Data Bank [11] for lineage M consists of only 16 colonies, whereas those for lineages A, C, and O are larger, consisting of 85, 37, and 49 colonies, respectively [45]. The M reference sample is clearly small, especially when considering the very large native distribution of this lineage, which extends from Iberia to western China [12]. Thus, a large portion of M lineage variation is inevitably underrepresented in the reference dataset. The intriguing detection of lineage A in Europe needs further investigation to determine whether hybrids between lineages C and M are being incorrectly classified as lineage A or the African wing phenotypes are present in Europe more often than was previously expected.

### Identification of unknown samples

When using the data provided here for the identification of an unknown sample, it is important to understand the limitations of the described methodology. If the unknown samples originate from one of the regions covered in the reference dataset, the results should be relatively accurate (i.e., the fraction of samples assigned to the correct region should be 98%). However, if the unknown samples originate from a region not covered in the reference dataset (e.g., Italy), they might be incorrectly classified as belonging to one of the regions or countries included in the identification model. In order to detect such misidentifications, the user should examine the probability of identification. If the probability is low, the unknown samples can be classified as outliers, as they will not match any of the geographical regions covered in the reference data. The threshold probability below which the samples are classified as outliers is arbitrary (here, we established a value of 0.001). Unfortunately, detection of outliers failed in many cases (e.g., in the case of samples from Italy). The lower identification rate of historic samples can be related to introgressive hybridization, which occurred in recent years in some parts of Europe [68]. The problem of false positives can be alleviated to some degree by adding contemporary samples from a wider geographic range to the identification model. The geographical coverage of Europe presented in this study is far from complete, as there are many countries without any data or with incomplete data. Among the countries included in this study, a better geographical coverage is warranted for Austria, Serbia, and Montenegro. The Austrian samples, which were obtained from a queen breeder, do not agree with the samples collected from the neighboring countries. This is particularly evident in the isolation-by-distance plot, where they are clear outliers (Fig. 7). This discrepancy may be related to artificial selection and drift, with the colonies being kept in genetic isolation from the surrounding population as a result of instrumental insemination. Ideally, reference samples should be collected from colonies in which queens are not sourced from breeding programs and that are widespread in the study area to ensure coverage of the genetic variation in the population. Another possibility is collecting bees from flowers, in which case they represent multiple nearby colonies.

Despite the problems related to introgression and the presence of false positives that were mentioned above, the wing data presented in this study can be used as a reference for future studies aimed at the identification and monitoring of nonnative honey bees. The wing shape of an unknown sample can be compared with a reference sample from a particular country or region. The identification results can be sorted by similarity to the reference, and colonies with the smallest similarity can be classified as nonnative. These outlier colonies can be re-queened or removed from the population. In this context, the presence of false positives is not a big problem because this procedure is focused on the detection of true negatives. In fact, beekeepers should re-queen only a fraction of their colonies, and the procedure developed here is focused on the detection of the most extreme outliers. Moreover, it is not essential that the reference sample perfectly represents the native phenotype for a particular geographical region. The full range of original variation, present in Europe before large-scale movements of nonnative honey bees, may be irreversibly lost. Fortunately, there is still considerable variation, and this variation deserves to be protected from further genetic erosion.

A large sample size of reference data should not be confused with the sample size of an unknown sample to be classified. In the latter case, one sample consisting of 10 to 20 workers from 1 colony or location should be sufficient. The identification may be based on only a single wing. However, in such cases, the results are inaccurate [36, 88]. By averaging multiple wings within a sample, the measurement error and influence of environmental (and not genetic) factors are minimized.

The conservation of honey bee biodiversity is often focused on the protection of certain subspecies. For example, there are efforts to preserve *A. m. mellifera* [89]. These efforts are justified because in some parts of western and northern Europe, native honey bees are threatened with extinction due to introgressive hybridization with nonnative bees [90, 91]. However, this approach can overlook some intraspecific variation. The subspecies concept oversimplifies the problem and attempts to classify a continuous variation into a categorical one. For example, different populations of *A. m. mellifera* within its wide range differ from each other much more than some subspecies do (e.g., *A. m. carnica* and *A. m. ligustica*) [30]. Relatively large intra-subspecific variation has been observed in *A. m. carnica, Apis mellifera macedonica* [92], *Apis mellifera iberiensis* [79, 83, 93], and *A. m. mellifera* [94]. Also, in Africa, there is clinal variation, which makes the discrimination of subspecies difficult [95, 96]. Continuous spatial variation is present even on the American continent, where the honey bee has been introduced [97]. In this study, we observed variation not only between countries but also within them. In general, there is isolation by distance, with smaller differences between bees from neighboring regions than between bees that are far apart [80, 94]. We do not advocate abandoning the well-established concept of subspecies but stress the importance of continuous variation, especially in the protection of honey bee diversity.

## Conclusion

The problem of a lack of reference samples can be solved by data sharing. Here, we provide for the first time a large collection of honey bee forewing images, accompanied by geographic coordinates as well as measurements and some additional data [1]. The collection and metadata are easy to review, reuse, and update. Moreover, we show how the data can be analyzed, for example, to predict the origin of an unknown sample. The dataset can be used, among other things, as a reference for future studies on biogeography and conservation of honey bees. We hope that future studies will also make wing images freely available in order to expand the dataset and improve our knowledge of honey bee geographic variation.

## Supplementary Material

giad019_GIGA-D-22-00297_Original_Submission

giad019_GIGA-D-22-00297_Revision_1

giad019_Response_to_Reviewer_Comments_Original_Submission

giad019_Reviewer_1_Report_Original_Submission Chris Armit -- 11/17/2022 Reviewed

giad019_Reviewer_1_Report_Revision_1 Chris Armit -- 2/10/2023 Reviewed

giad019_Reviewer_2_Report_Original_Submission Alexis Matamoro Vidal -- 11/29/2022 Reviewed

giad019_Reviewer_2_Report_Revision_1 Alexis Matamoro Vidal -- 2/10/2023 Reviewed

giad019_Supplemental_Figures

## Data Availability

The whole dataset, including the wing images, landmark coordinates, geographic coordinates of sampling locations, and other data, is available on the Zenodo website [1] under a public domain license. All details of the statistical analysis, including the identification of an unknown sample, are available at the WorkflowHub website [72]. All supporting data and materials are available in the *GigaScience* GigaDB database [98].

## Availability of Source Code and Requirements

- Project name: *Apis mellifera* wing images from Europe
- Project homepage: https://doi.org/10.5281/zenodo.7244070
- Operating system(s): Platform independent
- Programming language: R
- Other requirements: R (v. 4.0.3), numerous R packages described in the Methods section
- License: Public domain
- BiotoolsID: Apis-wings-EU
- WorkflowHub: https://doi.org/10.48546/WORKFLOWHUB.WORKFLOW.422.1

## Additional Files

**Supplementary Fig. S1**. Projection of the samples from different countries to canonical variate space of honey bee lineages published in Nawrocka et al. [45]. Ellipses indicate 95% confidence regions assuming multivariate *t*-distribution. Black ellipses indicated lineages; other ellipses indicate countries.

**Supplementary Fig. S2**. Samples from different countries classified as honey bee lineages. Jitter was used to show multiple samples from the same or similar location.

## Abbreviations

CVA: canonical variates analysis; GAM: generalized additive model; LDA: linear discriminant analysis; SNP: single-nucleotide polymorphism; UPGMA: unweighted pair group method with arithmetic mean.
