# Supplementary material for: Honey bee (Apis mellifera) wing images: a tool for identification and conservation
Source: Gigascience. 2023 Mar 27;12:giad019. doi: 10.1093/gigascience/giad019 (PMC10041535; doi:10.1093/gigascience/giad019)
Supplement: giad019_GIGA-D-22-00297_Original_Submission [file giad019_giga-d-22-00297_original_submission.pdf]

GigaScience

Honey bee (Apis mellifera) wing images: a tool for identification and conservation

--Manuscript Draft--

Manuscript Number:	GIGA-D-22-00297
Full Title:	Honey bee (Apis mellifera) wing images: a tool for identification and conservation
Article Type:	Data Note
Funding Information:	Fundação para a Ciência e a Tecnologia (UIDB/00690/2020) — Prof Maria Alice Pinto<br>Narodowe Centrum Nauki (2021/41/B/NZ9/03153) — Prof Adam Tofilski<br>Narodowe Centrum Nauki (2015/19/B/NZ9/03718) — Dr Andrzej Oleksa<br>Fundação para a Ciência e a Tecnologia (UIDP/00690/2020) — Prof Maria Alice Pinto

Abstract:

Background

The honey bee (Apis mellifera) is an ecologically and economically important species that provides pollination services to natural and agricultural systems. The biodiversity of the honey bee in parts of its native range is endangered by migratory beekeeping and commercial breeding. In consequence, some honey bee populations which are well adapted to the local environment are threatened by extinction. A crucial step for the protection of honey bee biodiversity is reliable differentiation between native and non-native bees. One of the methods which can be used for this is the geometric morphometrics of wings. It is fast, low-cost, and does not require expensive equipment. Therefore, it can be effectively used by both scientists and beekeepers. However, wing morphometrics is challenging due to the lack of reference data that can be reliably used for comparisons between different geographic regions.

Findings

Here, we provide an unprecedented collection of 26,481 honey bee wing images representing 1,725 samples from 13 European countries. The wing images are accompanied by coordinates of 19 landmarks and geographic coordinates of the sampling locations. We present an R script that describes the workflow for analysing the data and identifying an unknown sample. We compared the data with available reference samples for lineage and found general agreement with them.

Conclusions

The extensive collection of wing images available on Zenodo can be used to identify the geographic origin of unknown samples, and therefore assist in the monitoring and conservation of honey bee biodiversity in Europe.

Corresponding Author:	Adam Tofilski<br>Uniwersytet Rolniczy im. Hugona Kollataja w Krakowie<br>Krakow, POLAND
Corresponding Author Secondary Information:	
Corresponding Author's Institution:	Uniwersytet Rolniczy im. Hugona Kollataja w Krakowie
Corresponding Author's Secondary Institution:	
First Author:	Andrzej Oleksa
First Author Secondary Information:	
Order of Authors:	Andrzej Oleksa
	Eliza Căuia

Powered by Editorial Manager® and ProduXion Manager® from Aries Systems Corporation

	Adrian Siceanu
	Zlatko Puškadija
	Marin Kovačić
	Maria Alice Pinto
	Pedro João Rodrigues
	Fani Hatjina
	Leonidas Charistos
	Maria Bouga
	Janez Prešern
	Irfan Kandemir
	Slađan Rašić
	Szilvia Kusza
	Adam Tofilski
**Order of Authors Secondary Information:**	
**Additional Information:**	
**Question**	**Response**
Are you submitting this manuscript to a special series or article collection?	No
**Experimental design and statistics**<br><br>Full details of the experimental design and statistical methods used should be given in the Methods section, as detailed in our Minimum Standards Reporting Checklist. Information essential to interpreting the data presented should be made available in the figure legends.<br><br>Have you included all the information requested in your manuscript?	Yes
**Resources**<br><br>A description of all resources used, including antibodies, cell lines, animals and software tools, with enough information to allow them to be uniquely identified, should be included in the Methods section. Authors are strongly encouraged to cite Research Resource Identifiers (RRIDs) for antibodies, model organisms and tools, where possible.	Yes

Have you included the information requested as detailed in our Minimum Standards Reporting Checklist?	
**Availability of data and materials**<br><br>All datasets and code on which the conclusions of the paper rely must be either included in your submission or deposited in publicly available repositories (where available and ethically appropriate), referencing such data using a unique identifier in the references and in the "Availability of Data and Materials" section of your manuscript.<br><br>Have you have met the above requirement as detailed in our Minimum Standards Reporting Checklist?	Yes

Powered by Editorial Manager® and ProduXion Manager® from Aries Systems Corporation

Manuscript

Click here to access/download;Manuscript;Apis-mellifera-wings-GigaScience.docx

Honey bee (*Apis mellifera*) wing images: a tool for identification and conservation

Andrzej Oleksa, Department of Genetics, Faculty of Biological Sciences, Kazimierz Wielki University, Powstańców Wielkopolskich 10, 85-090 Bydgoszcz, Poland, olek@ukw.edu.pl

Eliza Căuia, Adrian Siceanu, Honeybee Genetics and Breeding Laboratory, Institute for Beekeeping Research and Development, Blv Ficusului, no. 42, Sector 1, 013975 Bucharest, Romania, eliza.cauia@yahoo.com, siceanu.adrian@gmail.com

Zlatko Puškadija, Marin Kovačić, Faculty of Agrobiotechnical Sciences, Josip Juraj Strossmayer University of Osijek, Osijek, Croatia, zlatko.puskadija@fazos.hr, Marin.Kovacic@fazos.hr

M. Alice Pinto, Centro de Investigação de Montanha, Instituto Politécnico de Bragança, Campus de Santa Apolónia, 5300-253, Bragança, Portugal, apinto@ipb.pt

Laboratório Associado para a Sustentabilidade e Tecnologia em Regiões de Montanha (SusTEC), Instituto Politécnico de Bragança, Campus de Santa Apolónia, 5300-253 Bragança, Portugal

Pedro João Rodrigues, Centre in Digitalization and Intelligent Robotics, Instituto Politécnico de Bragança, Campus de Santa Apolónia, 5300-253, Bragança, Portugal, pjsr@ipb.pt

Laboratório Associado para a Sustentabilidade e Tecnologia em Regiões de Montanha (SusTEC), Instituto Politécnico de Bragança, Campus de Santa Apolónia, 5300-253 Bragança, Portugal

Fani Hatjina, Leonidas Charistos, Department of Apiculture, Institute of Animal Science - Ellinikos Georgikos Organismos 'DIMITRA', 63200 Nea Moudania, Greece, fhatjina@elgo.gr, leocharistos@elgo.gr

Maria Bouga, Lab of Agricultural Zoology and Entomology, Agricultural University of Athens, 11855 Athens, Greece, mbouga@aua.gr

Janez Prešern, Agricultural Institute of Slovenia, Ljubljana, Slovenia, janez.presern@kis.si

Irfan Kandemir, Ankara University, Department of Biology, Faculty of Science, Ankara University, Beşevler-Ankara, Turkey, ikandemir@gmail.com

Slađan Rašić, Faculty of Ecological Agriculture, EDUCONS University, Vojvode Putnika 87, 21208 Sremska Kamenica, Serbia, rasic.sladjan@gmail.com

Szilvia Kusza, Centre for Agricultural Genomics and Biotechnology, University of Debrecen, 4032 Debrecen, Hungary, kusza@agr.unideb.hu

Adam Tofilski, Department of Zoology and Animal Welfare, University of Agriculture in Krakow, Krakow, Poland, rotofils@cyf-kr.edu.pl

Corresponding author: Adam Tofilski

Abstract

Background:

The honey bee (*Apis mellifera*) is an ecologically and economically important species that provides pollination services to natural and agricultural systems. The biodiversity of the honey bee in parts of its native range is endangered by migratory beekeeping and commercial breeding. In consequence, some honey bee populations which are well adapted to the local environment are threatened by extinction. A crucial step for the protection of honey bee biodiversity is reliable differentiation between native and non-native bees. One of the methods which can be used for this is the geometric morphometrics of wings. It is fast, low-cost, and does not require expensive equipment. Therefore, it can be effectively used by both scientists and beekeepers. However, wing morphometrics is challenging due to the lack of reference data that can be reliably used for comparisons between different geographic regions.

Findings:

Here, we provide an unprecedented collection of 26,481 honey bee wing images representing 1,725 samples from 13 European countries. The wing images are accompanied by coordinates of 19 landmarks and geographic coordinates of the sampling locations. We present an R script that describes the workflow for analysing the data and identifying an unknown sample. We compared the data with available reference samples for lineage and found general agreement with them.

Conclusions:

The extensive collection of wing images available on Zenodo can be used to identify the geographic origin of unknown samples, and therefore assist in the monitoring and conservation of honey bee biodiversity in Europe.

Keywords: honey bee, *Apis mellifera*, biodiversity, conservation, wing, geometric morphometrics,

Data description

Here we present 26,481 forewing images of honey bee workers. They represent 1,725 samples from 13 European countries (Table 1, Fig. 1). The shape of the wings was described using coordinates of 19 landmarks. The whole dataset, including the wing images, landmark coordinates, geographic coordinates of sampling locations and other data, is available on the Zenodo website [1] under a Creative Commons Attribution 4.0 licence.

Introduction

Honey bees (*Apis mellifera*) are ecologically and economically important. Their economic value as pollinators is much greater than that of honey or other products of the beekeeping industry [2]. At the same time, in the USA [3] and some European countries [4,5] there has been a decline in the number of honey bee colonies. Apart from pathogens, pesticides [6] and socioeconomic factors [7], loss of genetic variability is considered one of the possible causes of the declining population of managed honey bees [8–10].

Honey bees' native distribution range covers Europe up to a latitude of about 60 degrees north, Africa, the Middle East [11] and Central Asia [12,13]. Within this wide range, the environment varies markedly. Such diverse conditions and the history of spread and isolation of populations have resulted in notable variation of morphological and behavioural traits. There are more than 24 subspecies (geographical races) [11–18]. The subspecies were grouped into four major evolutionary lineages: A, C, M and O [11], which have recently been further subdivided [19].

The biodiversity of the honey bee is becoming endangered by the mass introduction of queens produced by breeding. This process began in the 19th century [20] and has intensified recently [21,22]. The honey bee queens used by many beekeepers are the daughters of relatively few selected breeder queens [23], which are often geographic hybrids [24]. In some European countries, more than 15% of all colonies are re-queened every year [25]. In consequence, some populations which are well adapted to the local environment [26] are threatened by extinction [21]. In this context it is important to distinguish native honey bees, which occurred in a particular area before the intensification of beekeeping, from non-native ones introduced into the area by human intervention.

The conservation of honey bee subspecies requires their identification, which may be based on molecular markers [27–29]. Recently, methods have been developed based on single nucleotide polymorphisms, which are more precise than microsatellites [30]. Their cost has recently been decreasing [28]; however, they are still relatively expensive and are not easily and quickly accessible to beekeepers. An alternative to molecular markers may be the identification of subspecies based on wing venation measurements [11,18,31]. The wings can be measured using various methodologies based on distances and angles [32], landmark coordinates [33–35], outlines [36] or image pixels [37]. While it has been demonstrated that morphological and molecular markers provide similar results [38,39], identification always requires a reference dataset, which is often inadequate.

The availability of data suitable for the identification of honey bee subspecies or evolutionary lineages using morphometry is relatively low. Early studies based on multiple measurements of wings provided averages and standard deviations for all measured distances and angles, as well as details related to linear discriminant analysis (LDA) [40,41]. Unfortunately, in later studies, including those covering global honey bee diversity, averages were not provided [32] or were provided only for a few selected variables [11]. For example, in the case of *A. m. mellifera* only 9 out of 36 characteristics were reported [11: Table 13.1]; among them there are no data for venation angles used for discrimination of this subspecies [11: page 229]. More importantly, the details of LDA were not presented in those studies. Consequently, readers could not use them to identify unknown samples. Later, the LDA details were provided only in a few studies of the honey bee [33,35,42–44]. Instead of providing LDA details, it would be even more useful to give readers access to all of the raw data used in the analysis, as has been done in some studies related to wing measurements in Diptera [45–47]. In the case of honey bees, only one study is known to us in which the landmark data were made available [48]. Providing landmark coordinates solves the problem of data availability only partially, because different studies may use different configurations of landmarks [18]. Not only can the order of landmarks differ between studies, but their position may also vary. This makes comparison between studies difficult or impossible. On the other hand, if a wing image is available, it can be re-analysed, and missing or incompatible landmarks can be determined. In addition, when available, the wing images can be used to determine the landmarks automatically [34,49,50].

Currently, while wing measurements are usually based on images, they are rarely made available after publication. This applies not only to honey bees, but also to other insects. Usually, only one image is presented with information about the position of the landmarks [45]. There are only a few examples in which wing images have been made publicly available. This is the case with one study of Vespidae [51], and *Drosophila*, for which a large repository of wing images was recently made available [52]. As far as we are aware, honey bee wing images have never been made available in more significant numbers.

The lack of reference data on the morphological variation of honey bees can be alleviated by data sharing, which is common practice in some scientific fields, including genomics [53] and neurosciences [54]. It is one of the factors that have facilitated the rapid

growth of those fields in recent years. In contrast to molecular biology, data sharing is relatively rare in ecological studies [55]. The benefits of data sharing within the scientific community are well known. However, individual authors often resist making their data available [56–58]. Even if some data are provided, they are often incomplete [59]. Large-scale investigations require large datasets [60], which are difficult for a single researcher to obtain. Individual studies on honey bee biogeography often focus on a relatively small area of one or a few countries, and large-scale comparisons are relatively rare [31,38,43]. Data sharing would allow the combining of datasets from multiple studies to obtain better knowledge about large-scale geographic variation and the conservation status of honey bee subspecies.

In an attempt to begin building a global reference dataset for the honey bee, here we provide an extensive repository of wing images representative of its diversity in a large part of Europe. The wing images are accompanied by the coordinates of 19 landmarks which can easily be used in future comparisons. We also present an R script in which we analyse the coordinates and show how they can be used to identify the origin of an unknown sample. Among other applications, the repository can be used to identify native honey bees, which is essential for their conservation.

Methods

Material

In this study we used 26,481 forewing images of honey bee workers (non-reproductive diploid females). They represent 1,725 samples from 13 European countries (Table 1, Fig. 1). A sample consists of workers that were collected either from one colony or from flowers in one location. The number of workers per sample ranged from 5 to 20. In some cases (in Poland and Hungary), when only one or two workers were collected from one location, neighbouring locations were treated together to obtain at least ten wings per sample. The samples from Austria, Montenegro and Serbia were obtained from queen breeders who use artificial selection. However, only in Austria were the breeding lines maintained by instrumental insemination of queens; in Montenegro and Serbia the bees were open-mated. Country names are abbreviated according to ISO 3166-1 (Table 1). The two-letter abbreviations were added at the beginning of file names to indicate the country of origin. The geographic coordinates of the samples, the year of their collection, and other information are provided in CSV files for each country separately.

Table 1. Sample size of honey bee wings used in this study.

Country	Country abbreviation	Number of wings	Number of samples
Austria	AT	198	10
Croatia	HR	6103	160
Greece	GR	1444	244
Hungary	HU	426	22
Moldova	MD	263	10
Montenegro	ME	300	20
Poland	PL	5955	253
Portugal	PT	960	192
Romania	RO	6498	197
Serbia	RS	299	20
Slovenia	SI	835	21
Spain	ES	2563	516
Turkey	TR	637	60

Some of the samples were already analysed in earlier studies addressing other goals: Croatia and Slovenia [61], Greece [62,63], Hungary and Poland in part [64,65], Portugal and Spain [38], Romania [66], Serbia and Montenegro [67], and Turkey [68]. Those studies used various methods, and their results could not be directly compared. Therefore, most of the wings were re-measured for this analysis. In none of the earlier studies were the wing images made publicly available. The data from Austria and Moldova have previously been neither analysed nor published.

Each wing from the dataset was saved in a separate PNG file. The wing image file name begins with the two-letter country code (Table 1), followed by a hyphen (identical to the minus sign), a four-digit sample code, another hyphen, and finally the original file name. The original file names vary as they originate from various studies. They usually consist of strings separated by hyphens. In some cases, the name ends with a letter L or R indicating left or right wing. The samples were numbered in each country separately; for this reason, the unique sample name has to include the two-letter country code. The wing images were sorted by country and compressed into 13 ZIP files.

In each wing image, the coordinates of 19 landmarks were determined. For a description of the landmarks, see Nawrocka et al. [43]. The landmarks are compatible with the "standard honey bee morphometry" used in earlier studies [32], and the landmark coordinates can be converted to distances and angles [as in 61]. The landmarks are saved within each wing image file and can be viewed and edited in the IdentiFly software application [43]. Raw coordinates of the landmarks were saved in CSV files for each country separately. The whole dataset, including the 26,481 forewing images, landmark coordinates, geographic coordinates of sampling locations and other data, is available on the Zenodo website [1] under a Creative Commons Attribution 4.0 licence.

Statistical analysis

The statistical analysis was performed in R (v. 4.0.3) [69]. All details of the statistical analysis are available as supplementary data (Supplementary Document 1). Landmark coordinates from all wings were superimposed using generalised Procrustes analysis in the geomorph package (v. 4.0.4) [70]. The aligned coordinates were averaged within samples, and the averages were used in the subsequent analysis. Principal component analysis was used to extract the first two principal components, which were used to describe how the wing shape varied geographically. The association between the principal components and geographic coordinates (latitude and longitude) was analysed using generalised additive model (GAM) regression in the mgcv package (v. 1.8-33) [71]. The differences between countries and regions were calculated by canonical variate analysis using the Morpho package (v. 2.9) [72]. The wing shape was clustered using UPGMA in the phangorn package (v. 2.5.5) [73]. The differences in wing shape between countries or regions were described using Mahalanobis distance. The correlation between those Mahalanobis distances and geographical distances was analysed using the Mantel test. The samples were classified under honey bee evolutionary lineages using IdentiFly software [43]. The reference samples used in the classification were obtained from the Morphometric Bee Data Bank in Oberursel, Germany.

Identification of an unknown wing sample

The data provided here can be used for the identification of an unknown sample of honey bee workers. The identification may be based on only a single wing; however, in such cases, the results are imprecise [35,74]. Use of a sample of at least ten wings from one colony or location is recommended. Here we used ten wing images from Spain (Supplementary Table 1). The landmarks were determined on all wings [43] and their coordinates were saved in a

CSV file. The coordinates were aligned using generalised Procrustes analysis to obtain consensus configuration of the unknown sample, which in turn was aligned with a consensus of the reference sample using ordinary Procrustes analysis in the shapes package (v. 1.2.6) [75]. Finally, after appropriate transformation, the CVA scores of an unknown sample were compared with CVA scores of reference samples to calculate the probabilities of its belonging to each group. The identification of an unknown sample can be achieved using an R script as provided in the supplementary materials (Supplementary Document 1). The user need only provide a CSV file with the coordinates of the unknown sample.

Results

Principal component analysis revealed relatively large variation in the wing shape. In the graph of the first two principal components, which account for 38.7% and 9.8% of the variance, respectively, at least two clear clusters of points are visible. One of the clusters represents the Iberian Peninsula, and the other Central and South-Eastern Europe. On the other hand, the second principal component differentiated Greece from the countries of Central Europe, particularly Austria (Fig. 2). The wing shape variation was strongly correlated with geographic location. Latitude and longitude correlated significantly with both the first and second principal components (GAM regression, PC1: EDF = 26.47, F = 512.9, $P < 10^{-16}$; PC2: EDF = 28.12, F = 62.07, $P < 10^{-16}$). The first principal component was much lower in the Iberian Peninsula than in the Balkans area (Fig. 3A). Additionally, it decreased in Poland from south to north and in Greece from west to east (Fig. 3A). The second principal component was highest in south-eastern Greece, decreasing towards north and west with some intricate patterns in Romania and Poland (Fig. 3B).

As expected, canonical variate analysis revealed deeper differences between countries and showed a similar pattern to principal component analysis (Fig. 4). The shape of honey bee wings (represented by 34 principal components) differed significantly among countries (MANOVA: F = 12.1, $P < 10^{-16}$). In pair-wise comparisons, most countries differed markedly from each other. Only Romania did not differ significantly from Moldova, Serbia from Montenegro, and Slovenia from Croatia and Hungary (Table 2). The largest Mahalanobis distance was found between samples from Portugal and Greece, and the smallest between samples from Portugal and Spain. The UPGMA tree shows more details about similarities between the wings collected from different countries (Fig. 5). Most neighbouring countries cluster together: Portugal with Spain, Greece with Turkey, Moldova with Romania, and Slovenia with Croatia. Isolation by distance was confirmed by a significant positive correlation between geographic distances and Mahalanobis distances of wing shape between countries (Mantel test: r = 0.7047, P = 0.0013). The Austrian samples did not fit well into this relationship. Despite their close geographic proximity to Slovenia, Croatia and Hungary, they had unexpectedly different wing shapes (Fig. 6).

Table 2. Differences between countries in wing shape (expressed as Mahalanobis distances, lower triangle) and significance of pair-wise comparisons (upper triangle). For country abbreviations see Table 1.

country	AT	ES	GR	HR	HU	MD	ME	PL	PT	RO	RS	SI	TR
AT	-	0.0001	0.0001	0.0001	0.0026	0.0009	0.0002	0.0001	0.0001	0.0001	0.0005	0.0001	0.0001
ES	10.3602	-	0.0001	0.0001	0.0001	0.0001	0.0001	0.0001	0.0001	0.0001	0.0001	0.0001	0.0001
GR	9.6709	11.3111	-	0.0001	0.0001	0.0001	0.0001	0.0001	0.0001	0.0001	0.0001	0.0001	0.0001
HR	7.4676	11.1342	5.2491	-	0.0250	0.0083	0.0001	0.0001	0.0001	0.0001	0.0001	0.0933	0.0001
HU	6.3694	10.9745	6.6387	2.9550	-	0.0155	0.0003	0.0011	0.0001	0.0025	0.0017	0.0524	0.0002
MD	7.4600	10.5178	6.7506	4.6274	5.1587	-	0.0059	0.0072	0.0001	0.0669	0.0160	0.0404	0.0012

ME	6.9289	10.5056	6.4158	5.2746	5.7927	5.8540	-	0.0001	0.0001	0.0001	0.8670	0.0001	0.0001
PL	6.9864	9.2408	6.2533	3.9108	3.9095	4.6505	6.2075	-	0.0001	0.0001	0.0001	0.0004	0.0001
PT	10.2711	1.8918	11.4833	11.2249	10.9754	10.3770	10.4285	9.1929	-	0.0001	0.0001	0.0001	0.0001
RO	6.3672	10.5980	5.8505	3.7678	3.8011	3.6076	4.8826	4.4288	10.5648	-	0.0003	0.0003	0.0001
RS	7.0790	10.7933	6.1816	4.7181	5.3638	5.2334	1.8720	5.8124	10.7111	4.5827	-	0.0002	0.0001
SI	8.0962	11.4743	5.9944	2.4516	3.5920	4.6343	6.5067	4.1484	11.4397	4.2440	5.8863	-	0.0001
TR	7.7594	10.2036	4.9768	4.3165	5.2857	5.7750	6.0269	5.1654	10.3717	4.3490	6.0440	5.1704	-

The samples were classified (with cross-validation) to their country of origin with a relatively high probability of success: 86.26%. Misclassifications most often occurred between neighbouring countries. For example, the correct classification rate for Portugal was only 79.69%, although all cases of misclassification occurred with neighbouring Spain. Many misclassifications can be attributed to the small sample size for some countries. When samples from some smaller countries were combined with those from their large neighbours (Portugal with Spain, Moldova with Romania, and Slovenia with Croatia), and other countries with sample sizes below 25 were excluded (Austria, Hungary, Montenegro, Serbia), the correct classification rate increased to 98.31%.

When the samples were classified as lineages, using the data from Nawrocka et al. [43], most of them (n = 850, 49.3%) were classified as lineage C, which occurred in all samples from six countries: Austria, Croatia, Hungary, Montenegro, Serbia and Slovenia. It was also most frequent in Greece, Moldova, Poland, Romania and Turkey. As expected, samples most similar to lineage C occurred in south-eastern Europe, except in south-eastern Greece (Fig. 7B).

The second most frequent was lineage M. It occurred in 638 samples (37.0%). It was dominant in Portugal and Spain, but also occurred in Poland. A clear similarity to lineage M was observed in the Iberian Peninsula. Moreover, similarity to this lineage increased in Poland from south to north (Fig. 7C).

Unexpectedly, a relatively large fraction of the samples (n = 188, 10.9%) was classified as lineage A. This was detected mainly in Poland, but also in the Iberian Peninsula, Greece, Turkey, and Moldova. Similarity to lineage A increased in Poland from south to north and in Greece from north-west to south-east; in the Iberian Peninsula, similarity to this lineage was moderate (Fig. 7A).

Lineage O was the least frequent (n = 49, 2.8%). It occurred in Greece and sporadically in Moldova, Poland, Romania, and the European part of Turkey. Similarity to lineage O increased in Greece from north-west to south-east (Fig. 7D).

Discussion

The data presented here show that the geographic variation of wing shape in Europe is still relatively large. The variation is most likely an effect of natural selection and not of beekeepers' mass introduction of non-native bees. When compared with historical reference samples from the Morphometric Bee Data Bank in Oberursel [11], the bees analysed here fit relatively well into the pattern, which is believed to be natural. In particular, there is a higher similarity to lineage M in the Iberian Peninsula and the north of Poland. Additionally, there is a higher similarity to lineage C in most of Central and South-Eastern Europe. In the eastern part of the Aegean Sea, there is a high similarity to lineage O, which occurred naturally in Turkey and Middle East. In general, the distribution of lineages presented in this study is in line with earlier studies based on morphometry [32], mitochondrial DNA [76,77], microsatellites [77–79] and single nucleotide polymorphisms [29,80]. On the other hand, there are some discrepancies, which will be discussed below.

It can be expected that the introduction of non-native bees will reduce geographic variation, because beekeepers prefer certain honey bee subspecies (*A. m. carnica, A. m. ligustica*, *A. m. caucasia*) or their hybrids. Beekeepers' preference for a limited number of breeding lines [23] may lead to a significant homogenisation of the population structure across the continent. It is worth noting, however, that despite the increased beekeeper-mediated gene flow in the last decades [21], our results indicate that honey bee diversity in Europe is still not lost, at least in eastern and southern Europe. The effective population size of honey bees is very large [the census population size is estimated at 11.6 million colonies in Europe; ,81]; hence, it may take many generations to change its genetic structure, and thus its phenotype. Free trade in breeding material has intensified only in the last several decades; hence, homogenisation of population structure may occur in the future unless remedial measures are taken to protect local genetic variability.

Some of the results from our study do not agree with the patterns that Ruttner [11] reported in his seminal work on honey bee taxonomy and biogeography. Many colonies in northern Poland and some other countries were classified as lineage A. This lineage is expected on the African continent and did not occur naturally in central and northern Europe [11]. One possible explanation for the presence of this lineage in Europe is human-induced introgression. A recent mitochondrial DNA survey in Central Europe detected haplotypes of African ancestry, although with a frequency of only 1.64% [64], which is much lower than that reported here for the forewing samples from Poland classified according to Nawrocka et al. [43] as lineage A (10.9%). The high proportion of samples assigned to lineage A may also be related to the fact that hybrids between lineages are more likely to be classified as lineage A. Those hybrids have an intermediate phenotype [82], similar to the mean shape of all lineages. In fact, the mean shape of all reference samples [Table S2 from 43] is classified by IdentiFly as lineage A, and its similarity to other lineages, particularly to lineage M, is much lower.

While hybridisation may be caused by the introduction of non-native bees by beekeepers, hybrids between lineages can also occur naturally. In the border between lineages M and C, apart from the Alps, there is no physical barrier. In such situation, a wide hybrid zone can be expected. Earlier studies reported that in Poland there is a wide transition zone with a clinal change in both morphological [83] and molecular [64] markers. This spatial change is most likely a natural phenomenon, because it was already present in the 1960s [83], when the importation and rearing of non-native bees was less common.

Hybrids between lineages can be identified to some degree using wing measurements [82]. However, this requires adequate reference samples that are not currently available. The reference sample from the Morphometric Bee Data Bank [11] for lineage M consists of only 16 colonies, whereas those for lineages A, C, and O are somewhat larger, consisting of 85, 37 and 49 colonies, respectively [43]. This reference sample is clearly small, especially when the native distribution of lineage M, which extends from Iberia to western China [12], is considered. Thus, a large portion of M lineage variation is inevitably underrepresented in the reference dataset. The intriguing detection of lineage A in Europe needs further investigation, to determine whether hybrids between lineages C and M are being incorrectly classified as lineage A, or the African wing phenotypes are present in Europe more often than was previously expected.

Even though the wing data presented in this study may be affected to some unknown degree by introgression, they can be used as a reference for future studies aimed at the identification and monitoring of non-native honey bees. An unknown sample can be compared in terms of wing shape with the reference sample of a particular country or region. If the similarity is smaller than some threshold, the sample can be classified as non-native, and the focal colony can be re-queened or removed from the population. In this context, it is

not essential for the reference sample to represent perfectly the native phenotype for a particular geographical region. The full range of original variation, present in Europe before large-scale movements of non-native honey bees, may be irreversibly lost. Fortunately, significant variation is still present and deserves to be protected from further genetic erosion.

The coverage of Europe presented in this study is far from complete. There are many countries without any data or with incomplete data. Among countries included in this study, better coverage is required for Austria, Serbia and Montenegro. The Austrian samples, which were obtained from a queen breeder, do not agree very well with the samples collected from neighbouring countries. This is particularly evident in the isolation-by-distance plot, where they are clear outliers (Fig. 6). This discrepancy may be related to artificial selection and drift, with the colonies being kept in genetic isolation from the surrounding population as a result of instrumental insemination. Ideally, reference samples should be collected from colonies in which queens are not sourced from breeding programmes and which are widespread in the study area, to ensure coverage of the genetic variation in the population. Another possibility is collecting bees from flowers, in which case they represent multiple nearby colonies. Apart from increasing the sample size and coverage, the data presented here can be improved by verification using molecular markers or by adding historical samples originating from times preceding the mass importation of non-native bees [66].

The conservation of honey bee biodiversity is often focused on the protection of certain subspecies. For example, there are efforts to preserve *A. m. mellifera* [84]. These efforts are justified, because in some parts of western and northern Europe native honey bees are threatened by extinction due to introgressive hybridisation with non-native bees [85,86]. However, this approach can overlook some intra-specific variation. The subspecies concept oversimplifies the problem and attempts to classify a continuous variation into a categorical one. For example, different populations of *A. m. mellifera* within its wide range differ from each other much more than some subspecies do (e.g., *A. m. carnica* and *A. m. ligustica*) [29]. Relatively large intra-subspecific variation has been observed in *A. m. carnica*, *A. m. macedonica* [87], *A. m. iberiensis* [76,80,88] and *A. m. mellifera* [89]. Also in Africa there is clinal variation which makes the discrimination of subspecies difficult [90,91]. Continuous spatial variation is present even on the American continent, where the honey bee has been introduced [92]. In this study, we have observed variation not only between countries, but also within them. In general, there is isolation by distance, with smaller differences between bees from neighbouring regions than those that are far apart [77,89]. We do not advocate abandoning the well-established concept of subspecies, but stress the importance of continuous variation, especially in the protection of honey bee diversity.

The problem of lack of reference samples can be solved by data sharing. Unfortunately, in ecology and conservation, data sharing is not a standard procedure [55]. The need for data sharing is widely accepted [93]. It is well known how to make the data available [94], and user-friendly repositories are freely available. Despite this, in most ecological studies, data are not shared, or they are incomplete and difficult to reuse [59]. It is sometimes suggested that ecologists do not share data because they are withholding them for their own future gain [55]. Apparently, they are not convinced by the promise of higher citation scores for publications accompanied by shared data [95].

We provide here for the first time a large collection of honey bee forewing images accompanied by geographic coordinates as well as measurements and some additional data [1], which is easy to review, reuse and update. Moreover, we show how the data can be analysed, for example, to predict the origin of an unknown sample. The dataset can be used, among other things, as a reference for future studies of the biogeography and conservation of honey bees. We hope that future studies will similarly make wing images freely available, in order to enlarge the dataset and improve our knowledge of honey bee geographic variation.

Availability of supporting data

The whole dataset, including the wing images, landmark coordinates, geographic coordinates of sampling locations and other data, is available on the Zenodo website [1] under a Creative Commons Attribution 4.0 licence. All details of the statistical analysis including identification of an unknown sample are available as supplementary data (Supplementary Document 1). The example of an unknown sample of honey bee wing measurements is available as supplementary data (Supplementary Table 1).

Abbreviations

AT: Austria, ES: Spain, GR: Greece, HR: Croatia, HU: Hungary, MD: Moldova, ME: Montenegro, PL: Poland, PT: Portugal, RO: Romania, RS: Serbia, SI: Slovenia, TR: Turkey, CVA: canonical variates analysis, LDA: linear discriminant analysis, GAM: generalised additive model, UPGMA: unweighted pair group method with arithmetic mean

Funding

This research was funded in part by the National Science Centre, Poland, grant numbers 2021/41/B/NZ9/03153 and 2015/19/B/NZ9/03718. M. Alice Pinto acknowledges Fundação para a Ciência e a Tecnologia for providing financial support from national funds (FCT/MCTES) to CIMO (UIDB/00690/2020 and UIDP/00690/2020) and SusTEC (LA/P/0007/2021).

Authors' contributions

AT and AO planned this study. All authors provided honey bee wing images from their countries. AT wrote the first version of the manuscript and all authors revised and contributed to its final version. All authors have read and approved the final manuscript.

Acknowledgements

Some samples from Poland were obtained within the framework of a project funded by the State Forests, "Pszczoły wracają do lasu" (Bees Return to the Forest), coordinated by Dr Kazimierz Szabla. Samples from Croatia were obtained during the project "Biodiversity of the honey bee (*Apis mellifera carnica*) population in the Republic of Croatia (BioBeeCro)" funded by the Paying Agency for Agriculture, Fisheries and Rural Development. Bernadeta Rzeźnicka assisted with the wing measurements.

References

1. Oleksa A, Căuia E, Siceanu A, Puškadija Z, Kovačić M, Pinto MA, et al.. Collection of wing images for conservation of honey bees (*Apis mellifera*) biodiversity in Europe. *Zenodo*. 2022; doi: 10.5281/zenodo.7244070.

2. Gallai N, Salles J-M, Settele J, Vaissière BE. Economic valuation of the vulnerability of world agriculture confronted with pollinator decline. *Ecological Economics*. 2009; doi: 10.1016/j.ecolecon.2008.06.014.

3. Ellis JD, Evans JD, Pettis J. Colony losses, managed colony population decline, and Colony Collapse Disorder in the United States. *Journal of Apicultural Research*. 2010; doi: 10.3896/IBRA.1.49.1.30.

4. Gray A, Brodschneider R, Adjlane N, Ballis A, Brusbardis V, Charrière J-D, et al.. Loss rates of honey bee colonies during winter 2017/18 in 36 countries participating in the

COLOSS survey, including effects of forage sources. *Journal of Apicultural Research*. 2019; doi: 10.1080/00218839.2019.1615661.

5. Potts SG, Roberts SPM, Dean R, Marris G, Brown MA, Jones R, et al.. Declines of managed honey bees and beekeepers in Europe. *Journal of Apicultural Research*. 2010; doi: 10.3896/IBRA.1.49.1.02.

6. Steinhauer N, Kulhanek K, Antúnez K, Human H, Chantawannakul P, Chauzat M-P, et al.. Drivers of colony losses. *Current Opinion in Insect Science*. 2018; doi: 10.1016/j.cois.2018.02.004.

7. Moritz RFA, Erler S. Lost colonies found in a data mine: Global honey trade but not pests or pesticides as a major cause of regional honeybee colony declines. *Agriculture, Ecosystems & Environment*. 2016; doi: 10.1016/j.agee.2015.09.027.

8. Panziera D, Requier F, Chantawannakul P, Pirk CWW, Blacquiere T. The diversity decline in wild and managed honey bee populations urges for an integrated conservation approach. *Frontiers in Ecology and Evolution*. 2022; doi: 10.3389/fevo.2022.767950.

9. Themudo GE, Rey-Iglesia A, Robles Tascón L, Bruun Jensen A, da Fonseca RR, Campos PF. Declining genetic diversity of European honeybees along the twentieth century. *Sci Rep*. 2020; doi: 10.1038/s41598-020-67370-2.

10. vanEngelsdorp D, Meixner MD. A historical review of managed honey bee populations in Europe and the United States and the factors that may affect them. *Journal of Invertebrate Pathology*. 2010; doi: 10.1016/j.jip.2009.06.011.

11. Ruttner F. Biogeography and taxonomy of honeybees. Berlin: Springer;

12. Chen C, Liu Z, Pan Q, Chen X, Wang H, Guo H, et al.. Genomic analyses reveal demographic history and temperate adaptation of the newly discovered honey bee subspecies *Apis mellifera sinisxinyuan* n. ssp. *Mol Biol Evol*. 2016; doi: 10.1093/molbev/msw017.

13. Sheppard WS, Meixner M. *Apis mellifera pomonella*, a new honey bee subspecies from Central Asia. *Apidologie*. 34:367–752003;

14. Bouga M, Alaux C, Bienkowska M, Büchler R, Carreck NL, Cauia E, et al.. A review of methods for discrimination of honey bee populations as applied to European beekeeping. *Journal of Apicultural Research*. 2011; doi: 10.3896/IBRA.1.50.1.06.

15. Engel MS. The taxonomy of recent and fossil honey bees (Hymenoptera : Apidae ; *Apis*). *Journal of Hymenoptera Research*. 8:165–961999;

16. Meixner MD, Costa C, Kryger P, Hatjina F, Bouga M, Ivanova E, et al.. Conserving diversity and vitality for honey bee breeding. *Journal of Apicultural Research*. 2010; doi: 10.3896/IBRA.1.49.1.12.

17. Meixner MD, Leta MA, Koeniger N, Fuchs S. The honey bees of Ethiopia represent a new subspecies of *Apis mellifera* — *Apis mellifera simensis* n. ssp. *Apidologie*. 42:425–372011;

18. Meixner MD, Pinto MA, Bouga M, Kryger P, Ivanova E, Fuchs S. Standard methods for characterising subspecies and ecotypes of *Apis mellifera*. *Journal of Apicultural Research*. 2013; doi: 10.3896/IBRA.1.52.4.05.

19. Dogantzis KA, Tiwari T, Conflitti IM, Dey A, Patch HM, Muli EM, et al.. Thrice out of Asia and the adaptive radiation of the western honey bee. *Science Advances*. American Association for the Advancement of Science; 2021; doi: 10.1126/sciadv.abj2151.

20. Crane E. The World History of Beekeeping and Honey Hunting. Routledge;

21. De la Rúa P, Jaffé R, Dall'Olio R, Muñoz I, Serrano J. Biodiversity, conservation and current threats to European honeybees. *Apidologie*. 2009; doi: 10.1051/apido/2009027.

22. Nielsdatter MG, Larsen M, Nielsen LG, Nielsen MM, Rasmussen C. History of the displacement of the European dark bee (Apis mellifera mellifera) in Denmark. *Journal of Apicultural Research*. Taylor & Francis; 2021; doi: 10.1080/00218839.2020.1826111.

23. Schiff NM, Sheppard WS. Genetic analysis of commercial honey bees (Hymenoptera: Apidae) from the southern United States. *J Econ Entomol*. 88:1216–201995;

24. Lodesani M, Costa C. Bee breeding and genetics in Europe. *Bee World*. 84:69–852003;

25. Chauzat M-P, Cauquil L, Roy L, Franco S, Hendrikx P, Ribière-Chabert M. Demographics of the European Apicultural Industry. *PLOS ONE*. Public Library of Science; 2013; doi: 10.1371/journal.pone.0079018.

26. Büchler R, Costa C, Hatjina F, Andonov S, Meixner MD, Conte YL, et al.. The influence of genetic origin and its interaction with environmental effects on the survival of *Apis mellifera* L. colonies in Europe. *Journal of Apicultural Research*. 2014; doi: 10.3896/IBRA.1.53.2.03.

27. Chen C, Parejo M, Momeni J, Langa J, Nielsen RO, Shi W, et al.. Population Structure and Diversity in European Honey Bees (Apis mellifera L.)—An Empirical Comparison of Pool and Individual Whole-Genome Sequencing. *Genes*. Multidisciplinary Digital Publishing Institute; 2022; doi: 10.3390/genes13020182.

28. Henriques D, Browne KA, Barnett MW, Parejo M, Kryger P, Freeman TC, et al.. High sample throughput genotyping for estimating C-lineage introgression in the dark honeybee: an accurate and cost-effective SNP-based tool. *Sci Rep*. Nature Publishing Group; 2018; doi: 10.1038/s41598-018-26932-1.

29. Momeni J, Parejo M, Nielsen RO, Langa J, Montes I, Papoutsis L, et al.. Authoritative subspecies diagnosis tool for European honey bees based on ancestry informative SNPs. *BMC Genomics*. 2021; doi: 10.1186/s12864-021-07379-7.

30. Muñoz I, Henriques D, Jara L, Johnston JS, Chávez-Galarza J, De La Rúa P, et al.. SNPs selected by information content outperform randomly selected microsatellite loci for delineating genetic identification and introgression in the endangered dark European honeybee (Apis mellifera mellifera). *Molecular Ecology Resources*. 2017; doi: 10.1111/1755-0998.12637.

31. Kandemir İ, Özkan A, Fuchs S. Reevaluation of honeybee (*Apis mellifera*) microtaxonomy: a geometric morphometric approach. *Apidologie*. 2011; doi: 10.1007/s13592-011-0063-3.

32. Ruttner F, Tassencourt L, Louveaux J. Biometrical-statistical analysis of the geographic variability of *Apis mellifera* L. *Apidologie*. 1978; doi: 10.1051/apido:19780408.

33. Francoy TM, Wittmann D, Drauschke M, Müller S, Steinhage V, Bezerra-Laure MA, et al.. Identification of Africanized honey bees through wing morphometrics: two fast and efficient procedures. *Apidologie*. 2008; doi: 10.1051/apido:2008028.

34. Rodrigues PJ, Gomes W, Pinto MA. DeepWings©: Automatic Wing Geometric Morphometrics Classification of Honey Bee (Apis mellifera) Subspecies Using Deep Learning for Detecting Landmarks. *Big Data and Cognitive Computing*. Multidisciplinary Digital Publishing Institute; 2022; doi: 10.3390/bdcc6030070.

35. Tofilski A. Using geometric morphometrics and standard morphometry to discriminate three honeybee subspecies. *Apidologie*. 2008; doi: 10.1051/apido:2008037.

36. Francoy TM, de Faria Franco F, Roubik DW. Integrated landmark and outline-based morphometric methods efficiently distinguish species of Euglossa (Hymenoptera, Apidae, Euglossini). *Apidologie*. 2012; doi: 10.1007/s13592-012-0132-2.

37. De Nart D, Costa C, Di Prisco G, Carpana E. Image recognition using convolutional neural networks for classification of honey bee subspecies. *Apidologie*. 2022; doi: 10.1007/s13592-022-00918-5.

38. Henriques D, Chávez-Galarza J, S. G. Teixeira J, Ferreira H, J. Neves C, Francoy TM, et al.. Wing Geometric Morphometrics of Workers and Drones and Single Nucleotide Polymorphisms Provide Similar Genetic Structure in the Iberian Honey Bee (<i>Apis mellifera iberiensis<\i>). *Insects*. 2020; doi: 10.3390/insects11020089.

39. Oleksa A, Tofilski A. Wing geometric morphometrics and microsatellite analysis provide similar discrimination of honey bee subspecies. *Apidologie*. 2015; doi: 10.1007/s13592-014-0300-7.

40. DuPraw EJ. The recognition and handling of honeybee specimens in non-Linnean taxonomy. *Journal of Apicultural Research*. 4:71–841965;

41. DuPraw EJ. Non-Linnean taxonomy and the systematics of honeybees. *Systematic Zoology*. 14:1–241965;

42. Bustamante T, Fuchs S, Grünewald B, Ellis JD. A geometric morphometric method and web application for identifying honey bee species (Apis spp.) using only forewings. *Apidologie*. 2021; doi: 10.1007/s13592-021-00857-7.

43. Nawrocka A, Kandemir İ, Fuchs S, Tofilski A. Computer software for identification of honey bee subspecies and evolutionary lineages. *Apidologie*. 2018; doi: 10.1007/s13592-017-0538-y.

44. Rinderer TE, Buco SM, Rubink WL, Daly HV, Stelzer JA, Riggio RM, et al.. Morphometric identification of Africanized and European honey bees using large reference populations. *Apidologie*. EDP Sciences; 1993; doi: 10.1051/apido:19930605.

45. López-García J, Angell C, Martín-Vega D. Wing morphometrics for the identification of Nearctic and Palaearctic Piophilidae (Diptera) of forensic relevance. *Forensic Science International*. 2020; doi: 10.1016/j.forsciint.2020.110192.

46. Szpila K, Żmuda A, Akbarzadeh K, Tofilski A. Wing measurement can be used to identify European blow flies (Diptera: Calliphoridae) of forensic importance. *Forensic Science International*. 2019; doi: 10.1016/j.forsciint.2019.01.001.

47. Szpila K, Johnston NP, Akbarzadeh K, Richet R, Tofilski A. Wing measurements are a possible tool for the identification of European forensically important Sarcophagidae. *Forensic Science International*. 2022; doi: 10.1016/j.forsciint.2022.111451.

48. Węgrzynowicz P, Łoś A. Dataset of wing venation measurements for Apis mellifera caucasica, A. mellifera carnica and A. mellifera mellifera (Hymenoptera: Apidae), their hybrids and backcrosses. *Biodivers Data J*. 2020; doi: 10.3897/BDJ.8.e53724.

49. Ángel-Beamonte E, Martín-Ramos P, Santolaria P, Sales E, Abizanda J, Yániz JL. Automatic determination of landmark coordinates for honey bee forewing venation using a new MATLAB-based tool. *Journal of Apicultural Research*. Taylor & Francis; 2018; doi: 10.1080/00218839.2018.1501856.

50. Batra SWT. Automatic image analysis for rapid identification of Africanized honey bees. In: Needham GR, editor. *Africanized honey bees and bee mites*. Ellis Horwood; p. 260–3.

51. Baracchi D, Dapporto L, Turillazzi S. Relevance of wing morphology in distinguishing and classifying genera and species of Stenogastrinae wasps. *Contributions to Zoology*. Brill; 2011; doi: 10.1163/18759866-08003003.

52. Sonnenschein A, VanderZee D, Pitchers WR, Chari S, Dworkin I. An image database of Drosophila melanogaster wings for phenomic and biometric analysis. *GigaScience*. 2015; doi: 10.1186/s13742-015-0065-6.

53. Kaye J, Heeney C, Hawkins N, de Vries J, Boddington P. Data sharing in genomics — re-shaping scientific practice. *Nat Rev Genet*. Nature Publishing Group; 2009; doi: 10.1038/nrg2573.

54. Eglen SJ, Marwick B, Halchenko YO, Hanke M, Sufi S, Gleeson P, et al.. Toward standard practices for sharing computer code and programs in neuroscience. *Nat Neurosci*. Nature Publishing Group; 2017; doi: 10.1038/nn.4550.

55. Parr CS, Cummings MP. Data sharing in ecology and evolution. *Trends in ecology & evolution*. 20:362–32005;

56. Huang X, Hawkins BA, Lei F, Miller GL, Favret C, Zhang R, et al.. Willing or unwilling to share primary biodiversity data: results and implications of an international survey. *Conservation Letters*. 2012; doi: 10.1111/j.1755-263X.2012.00259.x.

57. Moles A, Dickie JB, Flores-Moreno H. A response to Poisot et al.: Publishing your dataset is not always virtuous. *Ideas in Ecology and Evolution*. 62013;

58. Soranno PA, Cheruvelil KS, Elliott KC, Montgomery GM. It's Good to Share: Why Environmental Scientists' Ethics Are Out of Date. *BioScience*. 2014; doi: 10.1093/biosci/biu169.

59. Roche DG, Kruuk LEB, Lanfear R, Binning SA. Public Data Archiving in Ecology and Evolution: How Well Are We Doing? *PLOS Biology*. Public Library of Science; 2015; doi: 10.1371/journal.pbio.1002295.

60. Hampton SE, Strasser CA, Tewksbury JJ, Gram WK, Budden AE, Batcheller AL, et al.. Big data and the future of ecology. *Frontiers in Ecology and the Environment*. 11:156–622013;

61. Puškadija Z, Kovačić M, Raguž N, Lukić B, Prešern J, Tofilski A. Morphological diversity of Carniolan honey bee (*Apis mellifera carnica*) in Croatia and Slovenia. *Journal of Apicultural Research*. Taylor & Francis; 2020; doi: 10.1080/00218839.2020.1843847.

62. Bouga M, Hatjina F. Genetic variability in greek honey bee (A. mellifera L.) populations using geometric morphometrics analysis. *Proceedings of the Balkan scientific conference of biology in Plovdiv (Bulgaria)*. :598–602 2005;

63. Charistos L, Hatjina F, Bouga M, Mladenovic M, Maistros AD. Morphological Discrimination of Greek Honey Bee Populations Based on Geometric Morphometrics Analysis of Wing Shape. *Journal of Apicultural Science*. 2014; doi: 10.2478/jas-2014-0007.

64. Oleksa A, Kusza S, Tofilski A. Mitochondrial DNA Suggests the Introduction of Honeybees of African Ancestry to East-Central Europe. *Insects*. Multidisciplinary Digital Publishing Institute; 2021; doi: 10.3390/insects12050410.

65. Oleksa A, Tofilski A. Podgatunki pszczoły miodnej i rola lasów w ochronie ich różnorodności. *Ochrona owadów zapylających w ekosystemach leśnych (Eds Czekońska K, Szabla K)*. Krakow: Wydawnictwo Uniwersytetu Rolniczego w Krakowie;

66. Tofilski A, Căuia E, Siceanu A, Vișan GO, Căuia D. Historical Changes in Honey Bee Wing Venation in Romania. *Insects*. Multidisciplinary Digital Publishing Institute; 2021; doi: 10.3390/insects12060542.

67. Rašić S, Mladenović M, Stanisavljević L. Use of geometric morphometrics to differentiate selected lines of Carniolan honeybees (Apis mellifera carnica) in Serbia and Montenegro. *Archives of Biological Sciences*. 67:929–342015;

68. Çakmak İ, Fuchs S, Çakmak SS, Özkan Koca A, Nentchev P, Kandemir İ. Morphometric Analysis of Honeybees Distributed in Northern Turkey Along the Black Sea Coast. *Uludag Bee Journal*. Uludag University, Beekeeping Development Application & Research Center; 14:59–682014;

69. R Core Team. R: A Language and Environment for Statistical Computing. Vienna, Austria: R Foundation for Statistical Computing;

70. Baken EK, Collyer ML, Kaliontzopoulou A, Adams DC. geomorph v4.0 and gmShiny: Enhanced analytics and a new graphical interface for a comprehensive morphometric experience. *Methods in Ecology and Evolution*. 2021; doi: 10.1111/2041-210X.13723.

71. Wood SN. Fast stable restricted maximum likelihood and marginal likelihood estimation of semiparametric generalized linear models. *Journal of the Royal Statistical Society: Series B (Statistical Methodology)*. 2011; doi: 10.1111/j.1467-9868.2010.00749.x.

72. Schlager S. Morpho and Rvcg – Shape Analysis in R: R-Packages for Geometric Morphometrics, Shape Analysis and Surface Manipulations. In: Zheng G, Li S, Székely G, editors. *Statistical Shape and Deformation Analysis*. Academic Press;

73. Schliep KP. phangorn: phylogenetic analysis in R. *Bioinformatics*. 2011; doi: 10.1093/bioinformatics/btq706.

74. Oleksa A, Wilde J, Tofilski A, Chybicki IJ. Partial reproductive isolation between European subspecies of honey bees. *Apidologie*. 2013; doi: 10.1007/s13592-013-0212-y.

75. Dryden IL. shapes package. Vienna, Austria: R Foundation for Statistical Computing;

76. Chávez-Galarza J, Garnery L, Henriques D, Neves CJ, Loucif-Ayad W, Jonhston J. . S, et al.. Mitochondrial DNA variation of Apis mellifera iberiensis: further insights from a large-scale study using sequence data of the tRNAleu-cox2 intergenic region. *Apidologie*. 2017; doi: 10.1007/s13592-017-0498-2.

77. Franck P, Garnery L, Solignac M, Cornuet J-M. The Origin of West European Subspecies of Honeybees (Apis mellifera): New Insights from Microsatellite and Mitochondrial Data. *Evolution*. 1998; doi: 10.1111/j.1558-5646.1998.tb01839.x.

78. Garnery L, Franck P, Baudry E, Vautrin D, Cornuet J-M, Solignac M. Genetic diversity of the west European honey bee (Apis mellifera mellifera and A. m. iberia). II. Microsatellite loci. *Genetics Selection Evolution*. 30:S491998;

79. Kandemir İ, Meixner MD, Ozkan A, Sheppard WS. Genetic characterization of honey bee (*Apis mellifera cypria*) populations in northern Cyprus. *Apidologie*. 2006; doi: 10.1051/apido:2006029.

80. Chávez-Galarza J, Henriques D, Johnston JS, Carneiro M, Rufino J, Patton JC, et al.. Revisiting the Iberian honey bee (Apis mellifera iberiensis) contact zone: maternal and genome-wide nuclear variations provide support for secondary contact from historical refugia. *Molecular Ecology*. 2015; doi: 10.1111/mec.13223.

81. Jones R. European beekeeping in the 21st century: strengths, weaknesses, opportunities, threats. *Bee World*. 2004; doi: 10.1080/0005772X.2004.11099637.

82. Węgrzynowicz P, Gerula D, Tofilski A, Panasiuk B, Bieńkowska M. Maternal inheritance in hybrids of three honey bee subspecies. *Journal of Apicultural Science*. 2019; doi: 10.2478/jas-2019-0010.

83. Bornus L, Demianowicz A, Gromisz M. Morfologiczne badania krajowej pszczoły miodnej. *Pszczelnicze Zeszyty Naukowe*. 10:1–461966;

84. Parejo M, Wragg D, Gauthier L, Vignal A, Neumann P, Neuditschko M. Using Whole-Genome Sequence Information to Foster Conservation Efforts for the European Dark Honey Bee, *Apis mellifera mellifera*. *Front Ecol Evol*. Frontiers; 2016; doi: 10.3389/fevo.2016.00140.

85. Groeneveld LF, Kirkerud LA, Dahle B, Sunding M, Flobakk M, Kjos M, et al.. Conservation of the dark bee (Apis mellifera mellifera): Estimating C-lineage introgression in Nordic breeding stocks. *Acta Agriculturae Scandinavica, Section A — Animal Science*. Taylor & Francis; 2020; doi: 10.1080/09064702.2020.1770327.

86. Jensen AB, Palmer KA, Boomsma JJ, Pedersen BV. Varying degrees of *Apis mellifera ligustica* introgression in protected populations of the black honeybee, *Apis mellifera mellifera*, in northwest Europe. *Molecular Ecology*. 2005; doi: 10.1111/j.1365-294X.2004.02399.x.

87. Muñoz I, De la Rúa P. Wide genetic diversity in Old World honey bees threaten by introgression. *Apidologie*. 2021; doi: 10.1007/s13592-020-00810-0.

88. Cánovas F, De la Rúa P, Serrano J, Galián J. Geographical patterns of mitochondrial DNA variation in Apis mellifera iberiensis (Hymenoptera: Apidae). *Journal of Zoological Systematics and Evolutionary Research*. 2008; doi: 10.1111/j.1439-0469.2007.00435.x.

89. Miguel I, Iriondo M, Garnery L, Sheppard WS, Estonba A. Gene flow within the M evolutionary lineage of Apis mellifera: role of the Pyrenees, isolation by distance and post-glacial re-colonization routes in the western Europe. *Apidologie*. 2007; doi: 10.1051/apido:2007007.

90. Diniz-Filho JA, Hepburn HR, Radloff S, Fuchs S. Spatial analysis of morphological variation in African honeybees (*Apis mellifera* L.) on a continental scale. *Apidologie*. 31:191–2042000;

91. Hepburn HR, Radloff SE. Honeybees of Africa. Springer Science & Business Media;

92. Daly HV, Hoelmer K, Gambino P. Clinal geographic variation in feral honey bees in California, USA. *Apidologie*. 22:591–6091991;

93. Whitlock MC. Data archiving in ecology and evolution: best practices. *Trends in Ecology & Evolution*. 26:61–52011;

94. White EP, Baldridge E, Brym ZT, Locey KJ, McGlinn DJ, Supp SR. Nine simple ways to make it easier to (re)use your data. *Ideas in Ecology and Evolution*. 62013;

95. Peters I, Kraker P, Lex E, Gumpenberger C, Gorraiz JI. Zenodo in the Spotlight of Traditional and New Metrics. *Frontiers in Research Metrics and Analytics*. 22017;

Figure legends

Fig. 1. Locations from which samples were collected. Jitter is used to show multiple samples from the same or similar location.

Fig. 2. The first two principal components of wing shape.

Fig. 3. First (A) and second (B) principal component interpolated over sampling locations using a generalised additive model.

Fig. 4. Discrimination between countries based on the first two canonical variates.

Fig. 5. UPGMA tree illustrating similarities between the shape of the wings collected from different countries.

Fig. 6. Relationship between geographical distance and Mahalanobis distance among countries.

Fig. 7. Mahalanobis distance to lineage A (A), C (B), M (C) and O (D) interpolated over sampling locations using a generalised additive model.

Figure 1

Click here to access/download;Figure;fig1.pdf

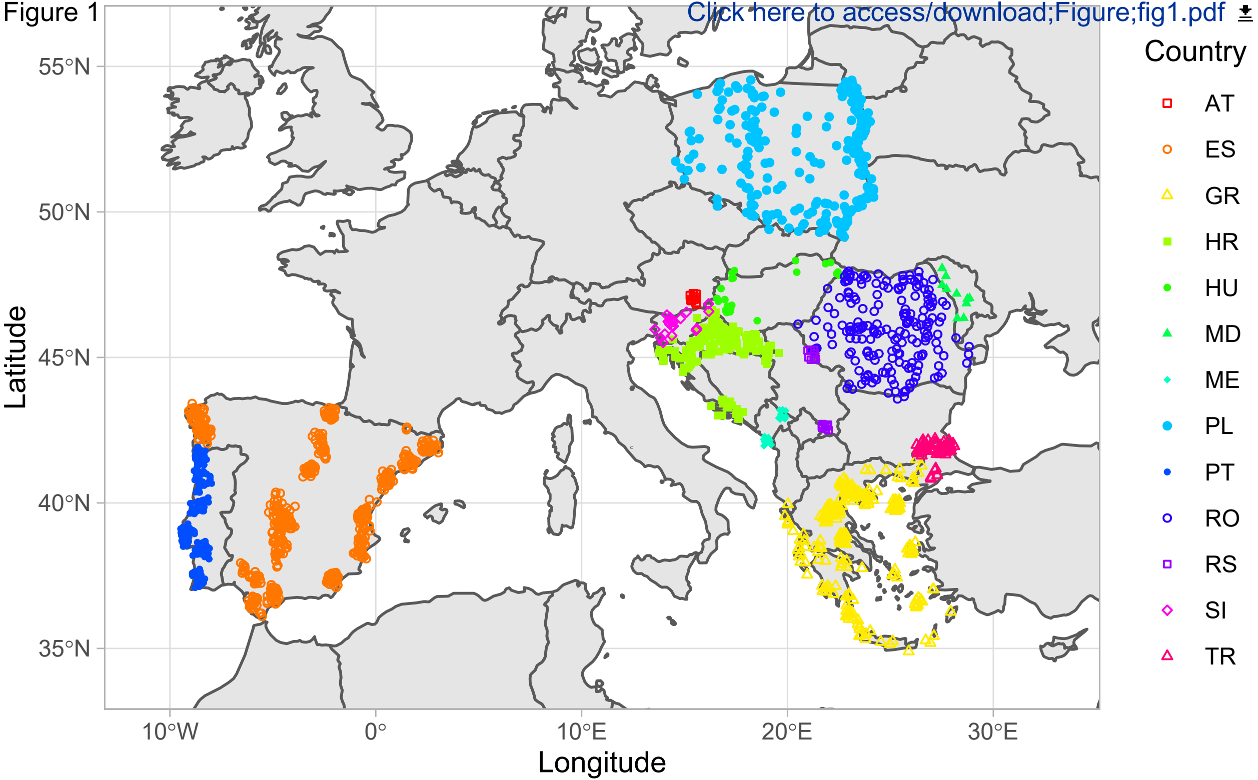

Figure 2

Click here to access/download;Figure;fig2.pdf

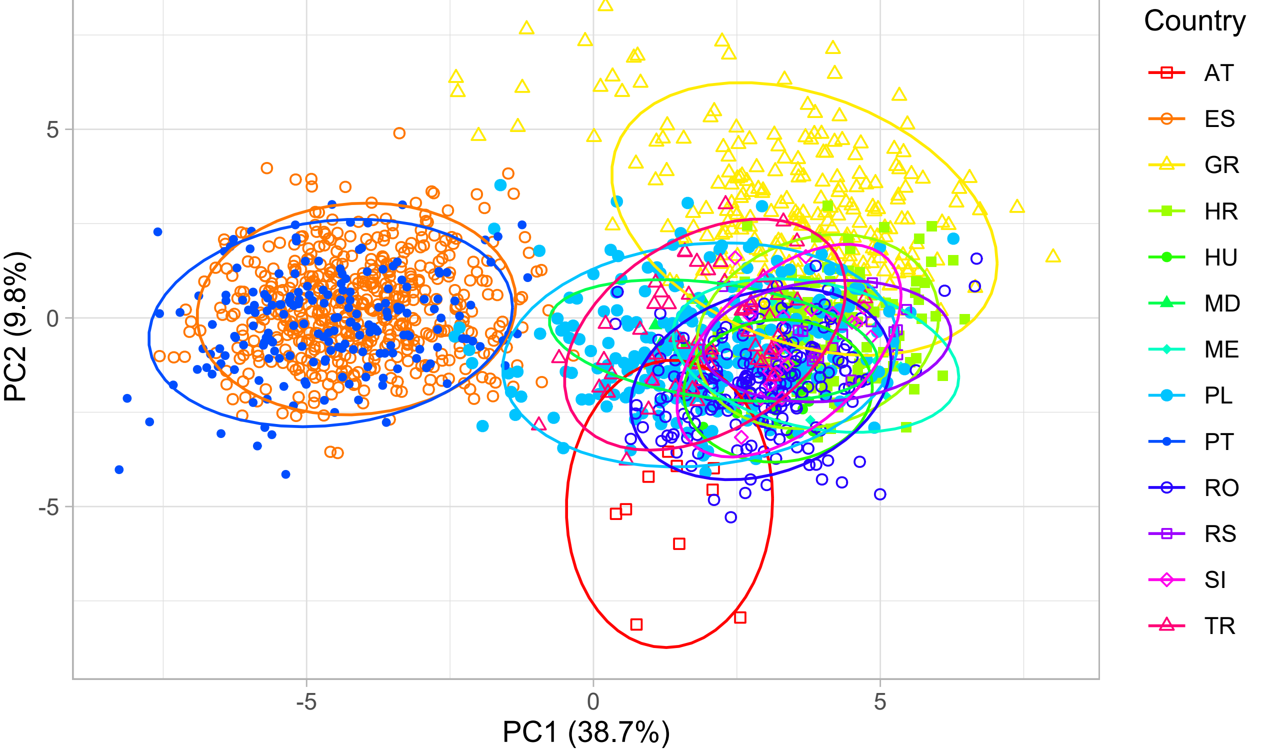

Figure 3

Click here to access/download;Figure;fig3.pdf

A

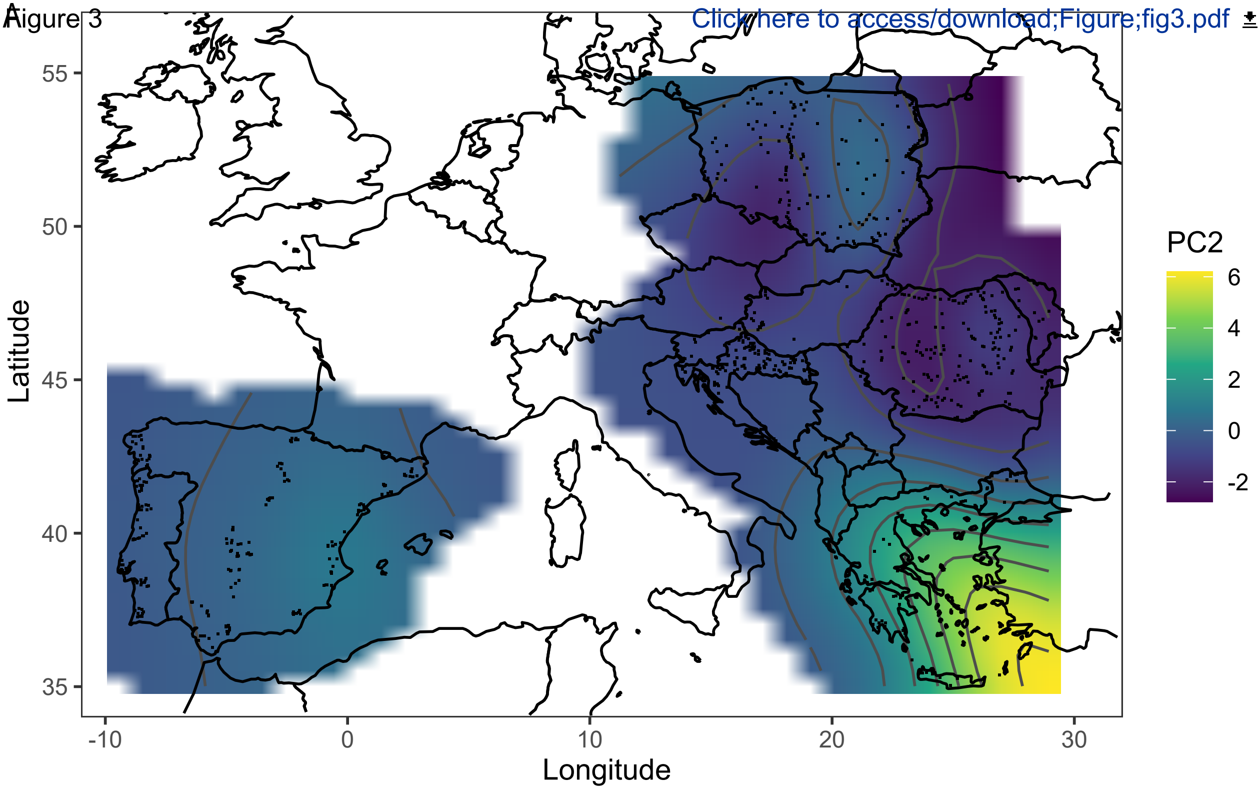

B

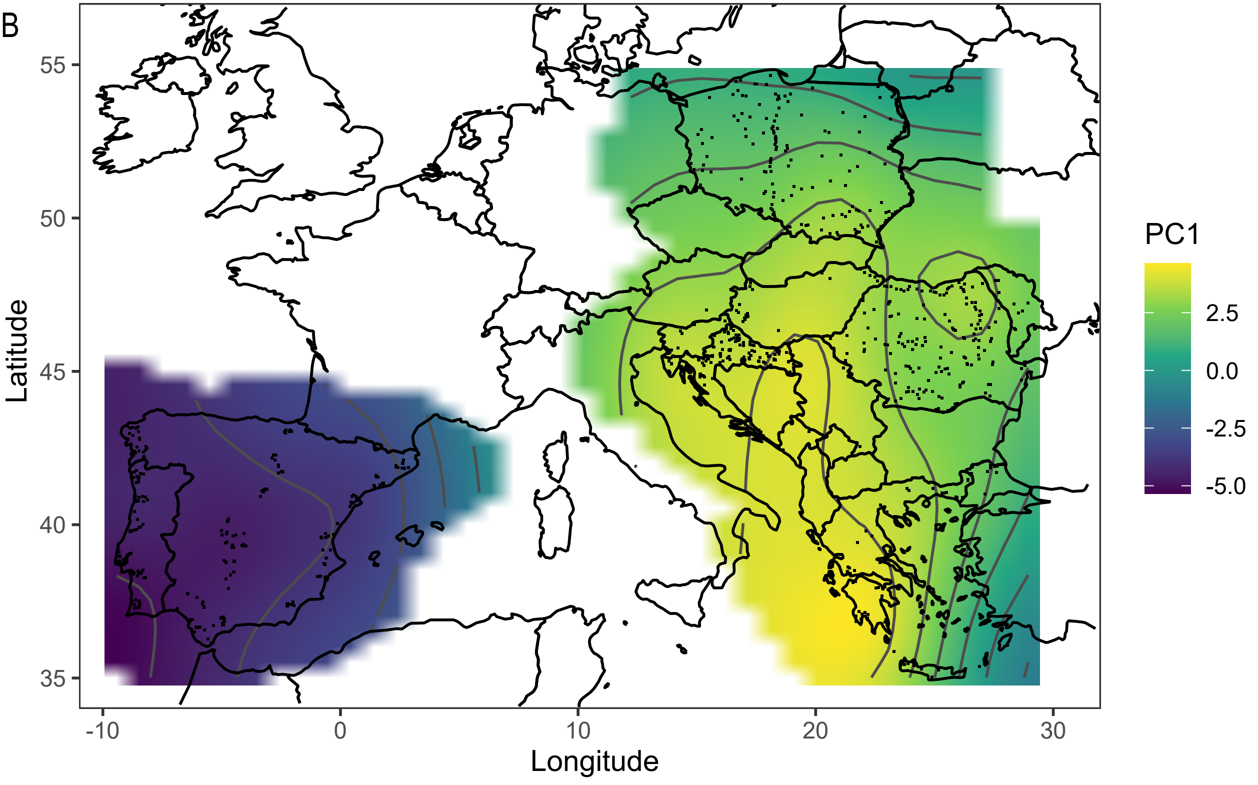

Figure 4

Click here to access/download;Figure;fig4.pdf

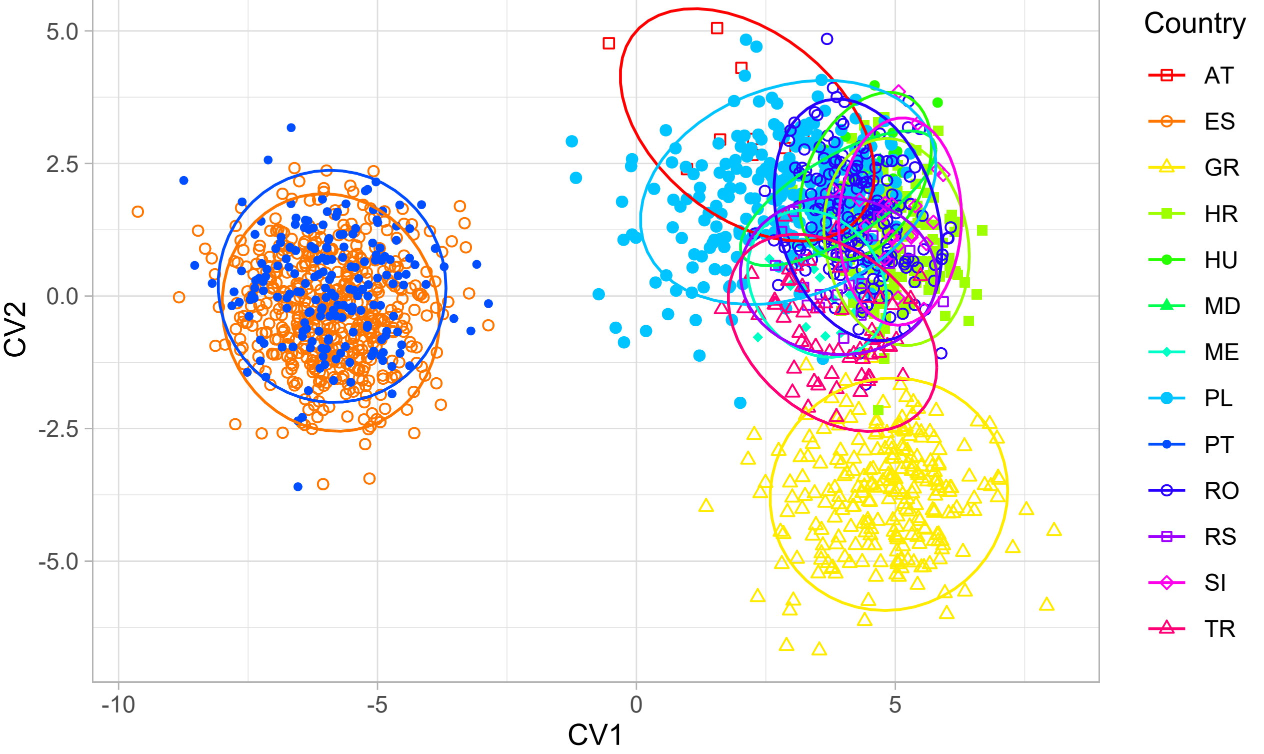

Figure 5

Click here to access/download;Figure;fig5.pdf

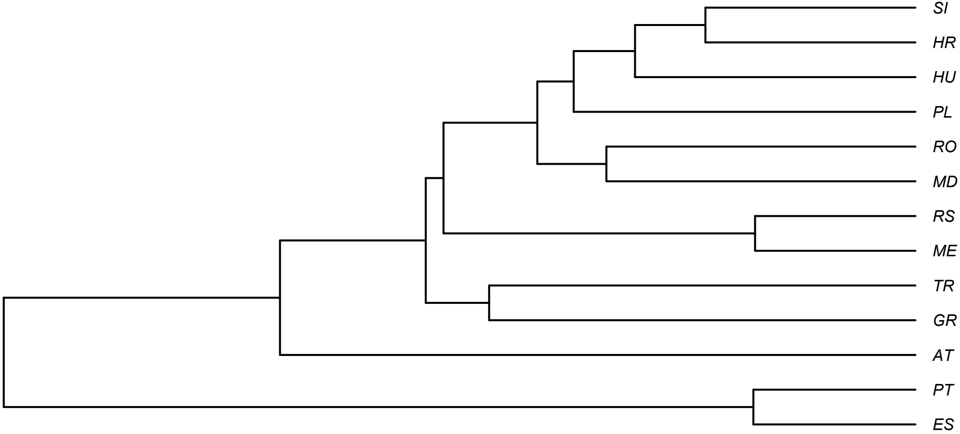

Figure 6

Click here to access/download;Figure;fig6.pdf

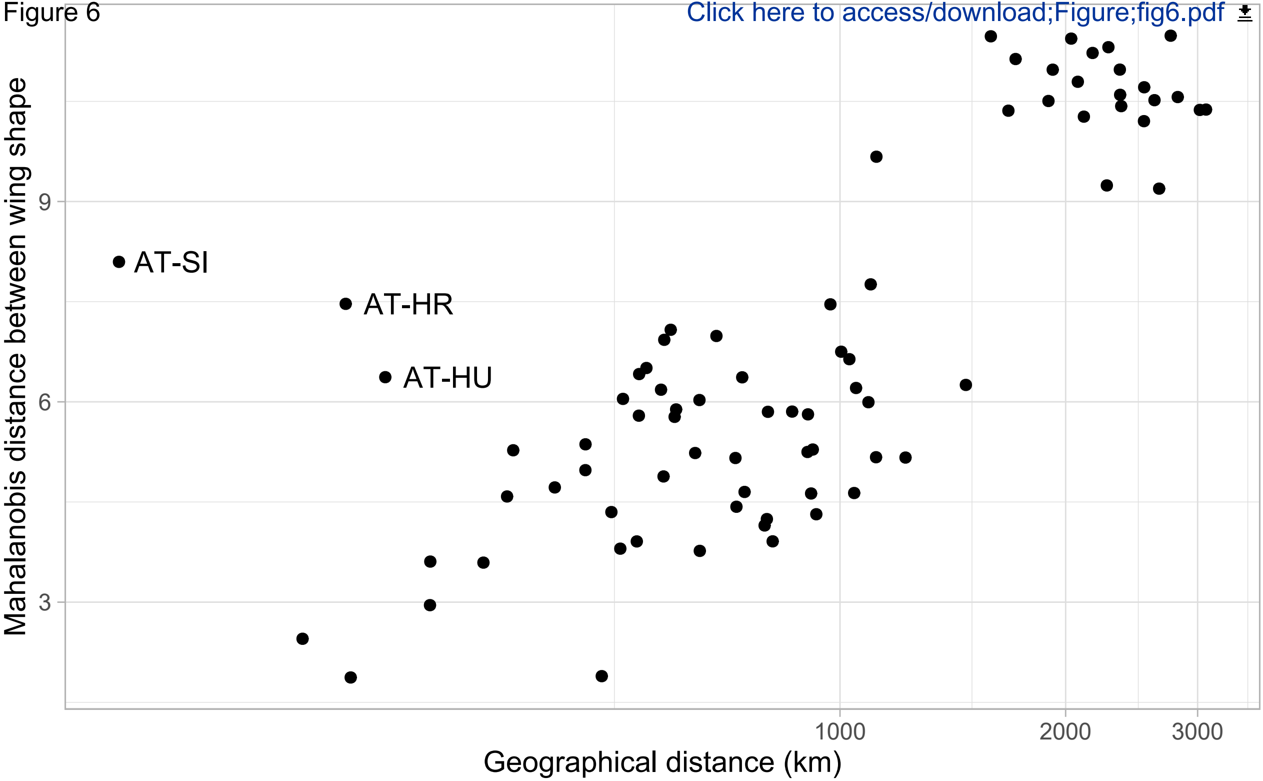

Figure 7

Click here to access/download;Figure;fig7.pdf

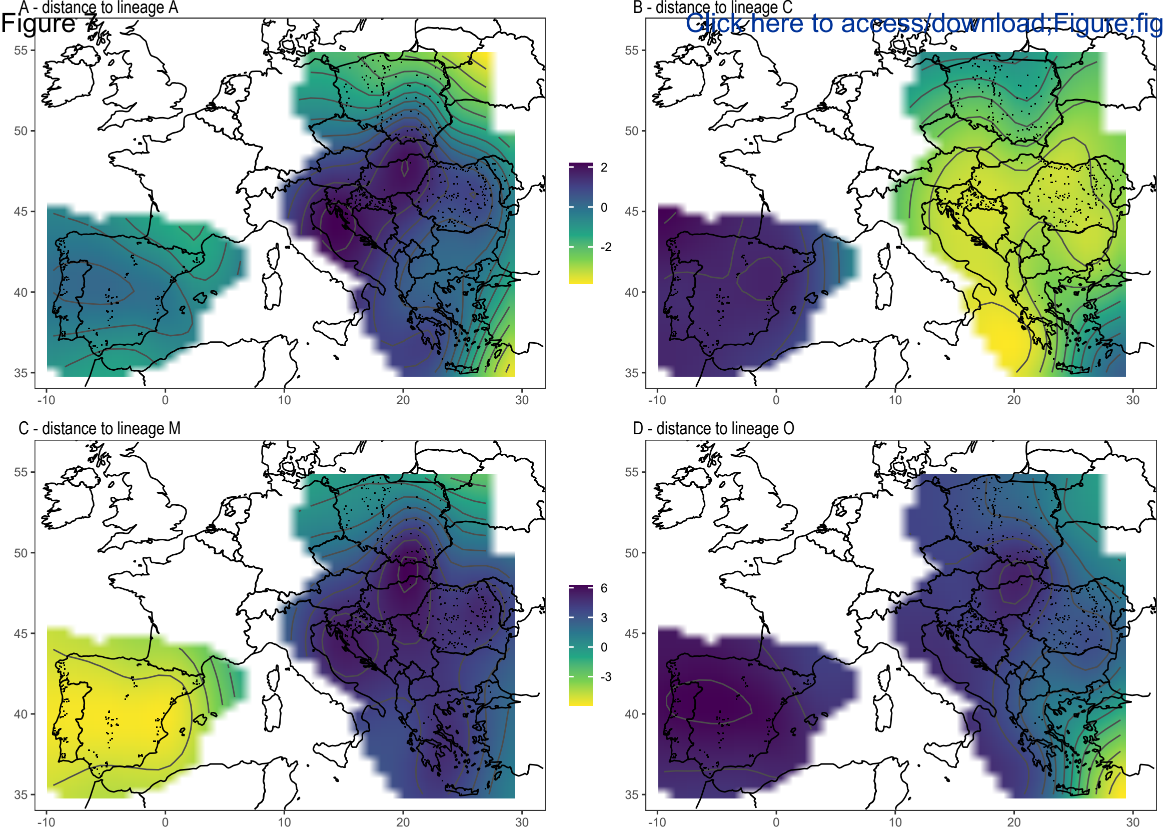

Supplementary Document 1

Click here to access/download
Supplementary Material
Document-1-supplementary-material.html

Supplementary Table S1

Click here to access/download
Supplementary Material
Table-S1-supplementary-material.csv
