# Supplementary material for: Honey bee (Apis mellifera) wing images: a tool for identification and conservation
Source: Gigascience. 2023 Mar 27;12:giad019. doi: 10.1093/gigascience/giad019 (PMC10041535; doi:10.1093/gigascience/giad019)
Supplement: giad019_GIGA-D-22-00297_Revision_1 [file giad019_giga-d-22-00297_revision_1.pdf]

GigaScience

Honey bee (Apis mellifera) wing images: a tool for identification and conservation

--Manuscript Draft--

**Manuscript Number:**	GIGA-D-22-00297R1
**Full Title:**	Honey bee (Apis mellifera) wing images: a tool for identification and conservation
**Article Type:**	Data Note
**Funding Information:**	Narodowe Centrum Nauki (2021/41/B/NZ9/03153) — Prof Adam Tofilski<br>Narodowe Centrum Nauki (2015/19/B/NZ9/03718) — Dr Andrzej Oleksa<br>Narodowe Centrum Badań i Rozwoju (TANGO-V-A/0042/2021) — Prof Adam Tofilski<br>Fundação para a Ciência e a Tecnologia (UIDB/00690/2020) — Prof Maria Alice Pinto<br>Fundação para a Ciência e a Tecnologia (UIDP/00690/2020) — Prof Maria Alice Pinto<br>SusTEC (LA/P/0007/2021) — Prof Maria Alice Pinto<br>Javna Agencija za Raziskovalno Dejavnost RS (P4-0431 NextGenAgri) — Dr Janez Prešern

Funder	Recipient
Narodowe Centrum Nauki (2021/41/B/NZ9/03153)	Prof Adam Tofilski
Narodowe Centrum Nauki (2015/19/B/NZ9/03718)	Dr Andrzej Oleksa
Narodowe Centrum Badań i Rozwoju (TANGO-V-A/0042/2021)	Prof Adam Tofilski
Fundação para a Ciência e a Tecnologia (UIDB/00690/2020)	Prof Maria Alice Pinto
Fundação para a Ciência e a Tecnologia (UIDP/00690/2020)	Prof Maria Alice Pinto
SusTEC (LA/P/0007/2021)	Prof Maria Alice Pinto
Javna Agencija za Raziskovalno Dejavnost RS (P4-0431 NextGenAgri)	Dr Janez Prešern

Abstract:

Background

The honey bee (Apis mellifera) is an ecologically and economically important species that provides pollination services to natural and agricultural systems. The biodiversity of the honey bee in parts of its native range is endangered by migratory beekeeping and commercial breeding. In consequence, some honey bee populations that are well adapted to the local environment are threatened with extinction. A crucial step for the protection of honey bee biodiversity is reliable differentiation between native and non-native bees. One of the methods that can be used for this is the geometric morphometrics of wings. This method is fast, low-cost, and does not require expensive equipment. Therefore, it can be easily used by both scientists and beekeepers. However, wing geometric morphometrics is challenging due to the lack of reference data that can be reliably used for comparisons between different geographic regions.

Findings

Here, we provide an unprecedented collection of 26,481 honey bee wing images representing 1,725 samples from 13 European countries. The wing images are accompanied by the coordinates of 19 landmarks and the geographic coordinates of the sampling locations. We present an R script that describes the workflow for analysing the data and identifying an unknown sample. We compared the data with available reference samples for lineage and found general agreement with them.

Conclusions

The extensive collection of wing images available on the Zenodo website (https://zenodo.org/record/7244070) can be used to identify the geographic origin of unknown samples and therefore assist in the monitoring and conservation of honey bee biodiversity in Europe.

**Corresponding Author:**	Adam Tofilski<br>Uniwersytet Rolniczy im. Hugona Kollataja w Krakowie<br>Krakow, POLAND
**Corresponding Author Secondary Information:**	
**Corresponding Author's Institution:**	Uniwersytet Rolniczy im. Hugona Kollataja w Krakowie
**Corresponding Author's Secondary**	

Powered by Editorial Manager® and ProduXion Manager® from Aries Systems Corporation

**Institution:**	
**First Author:**	Andrzej Oleksa
**First Author Secondary Information:**	
**Order of Authors:**	Andrzej Oleksa
	Eliza Căuia
	Adrian Siceanu
	Zlatko Puškadija
	Marin Kovačić
	Maria Alice Pinto
	Pedro João Rodrigues
	Fani Hatjina
	Leonidas Charistos
	Maria Bouga
	Janez Prešern
	Irfan Kandemir
	Slađan Rašić
	Szilvia Kusza
	Adam Tofilski
**Order of Authors Secondary Information:**	
**Response to Reviewers:**	Dear Hongling Zhou,<br>Please find attached the revised manuscript entitled “Honey bee (Apis mellifera) wing images: a tool for identification and conservation” (manuscript number: GIGA-D-22-00297), which we hope you will find suitable for publication in GigaScience as Data Note.<br>We are grateful to both Reviewers for their constructive comments and corrections. We have revised the manuscript according to most of them. In addition, we made corrections to the English across the manuscript to improve grammar and its readability. We believe that the changes have improved the manuscript substantially.<br>Please find below our point-by-point responses to the Reviewer, which we indicate with subheadings “Response:”.<br>Yours sincerely,<br>Adam Tofilski<br><br>Reviewer #1: This exceptional Data Note reports on honey bee (Apis mellifera) wing images, and showcases how analysis of wing venation patterns can be used to aid honey bee conservation. The authors highlight that "a crucial step for the protection of honey bee biodiversity is reliable differentiation between native and non-native bees" and towards this end provide 26,481 honey bee wing images representing 1,725 samples from 13 European countries. The supporting dataset is archived in Zenodo and the images are of exceptional quality and have very high contrast.<br><br>In their analysis, the authors segment the wing venation patterns and use image analysis to delineate branch points. These branch points are anatomical landmark points that are then used in a morphometric analysis to identify honey bee subspecies. The wing venation analysis approach utilised in this Data Note has been published previously [Nawrocka et al. Computer software for identification of honey bee subspecies and evolutionary lineages. Apidologie 49, 172-184 (2018). https://doi.org/10.1007/s13592-017-0538-y]. The position of all 19 landmarks in honey bee wing is is conveniently summarised in the following figure from the original Nawrocka et al (2018) paper [see https://link.springer.com/article/10.1007/s13592-017-0538-y/figures/1].

Powered by Editorial Manager® and ProduXion Manager® from Aries Systems Corporation

An important feature of this Data Note is that the authors provide details of geographic (latitude / longitude) coordinates for all honey bees included in the study. Principal Component Analysis of geographic coordinates and honey bee wing landmark coordinates is used to describe how the wing shape varies geographically. The R scripts used in this study are very nicely documented in a supporting HTML file [see Document-1-supplementary-material.html]. As a use case example, the authors showcase how this approach can be used to identify the origin of an unknown sample.

The supporting data, including all wing images, are archived in Zenodo where they have been ascribed a Public Domain Dedication (CC0). The Zenodo repository includes the following:

*26,481 honey bee wing images representing 1,725 samples from 13 European countries
*Landmark coordinates of wing venation patterns
*Geographic coordinates of honey bee samples
*Map visualisation of geographic coordinates of honey bee samples

For a subset of the image data, additional details of image resolution (pixels per metre) are provided. From a reuse perspective, this is a significant addition as it enables more traditional morphometric analysis (length, width, etc) to be performed on this subset of image data.
Response:
Thank you for summarising our manuscript with some positive notes which are very encouraging to us.

Minor point

I attempted to run the supporting R scripts on MacOS. Whereas many of the R scripts worked, some of the supporting libraries could not be installed and so, for some of the scripts, I was unable tor reproduce the results. This could be due to the version of R (version 3.5.2) that I was using. To illustrate the problem, I attach a screen capture of the issue I experienced when attempting to install the package "phangorn".
Response:
In order to reproduce our results, users need to use a recent version of R (v. 4.0.3). In particular, the package phangorn requires R in a version later than 3.5.2. The first two screenshots with information about problems were probably done before a R upgrade. The last screenshots show the R version 4.2.2; at this stage, package phangorn should work without problem, as long as all its dependencies listed at the bottom of the last screenshots are properly installed.

Towards this end, I invite the authors to detail any additional requirements (e.g. platform dependencies, version of R) that are required to reproduce the analysis showcased in the manuscript. These details should be included in a section of the manuscript entitled "Availability of source code and requirements" as described in the GigaScience Data Note instructions (see https://academic.oup.com/gigascience/pages/data_note), and should include the following:

*Project name: e.g. My bioinformatics project
*Project home page: e.g. http://sourceforge.net/projects/mged
*Operating system(s): e.g. Platform independent
*Programming language: e.g. Java
*Other requirements: e.g. Java 1.3.1 or higher, Tomcat 4.0 or higher
*License: e.g. GNU GPL, FreeBSD etc.
*RRID: if applicable, e.g. RRID: SCR_014986
Response:
In the lines 474-483, we added a section "Availability of source code and requirements" with the relevant information. More detailed information about R and packages can be found at the end of Supplementary Document 1 in section "Information about session".
We added information about this project to: bio.tools, and workflowhub.eu.
We attempted to add RRID but our submission to scicrunch.org was rejected because our dataset is not “biomedical” and is not related to “antibodies, organisms, chemicals, plasmids or cell lines”

Powered by Editorial Manager® and ProduXion Manager® from Aries Systems Corporation

Reviewer #2: information copied from the attached PDF
In this manuscript, Oleksa et al. present a dataset of > 20.000 honey bee wings images. Each image is accompanied with shape descriptors (19 landmarks) and geographic sampling location. In addition, the authors provide an R script aiming at retrieving the shape and geographic descriptors, allowing to perform multivariate analyses in order (mainly) to: 1/ test whether wing shape descriptors allow to discriminate specimens according to their geographical origin. 2/ assign an unknown sample to a geographical location / evolutionary lineage. The R script allows also to run some other analyses. I think this is a valuable dataset. The R pipeline provided by the authors works smoothly and their analysis pipeline is accurate. Below are some suggestions that could help in improving the manuscript.
Response:
Thank you for your positive comments.

General comment:
According to the abstract, a major objective of the study is to provide a reference dataset that can be used to classify an “unknown sample”. However, this aspect is not given enough importance in the analysis. The coordinates of an “unknown sample” are given as supplementary material but I have not found the analysis of this “unknown sample” in the main text of the manuscript (although such analysis is given in the R script). I recommend to the authors to dedicate a section to this in the “results” section of the manuscript, to show the reader how good is the approach to assign a location to an “unknown sample”.
To make this point stronger, I would appreciate if independent samples from different locations/evolutionary lineages are tested to further investigate how good is the approach to correctly assign a geographic location and/or evolutionary lineage to a given sample. By doing this, the authors could provide clear guidelines about the conditions for which the method “works” (i.e., correctly assign an unknown sample to a geographic location/evolutionary lineage). For example, from the data it seems clear that the method is good in discriminating Spain/Portugal, Greece and Turkey groups (Figure 2) but that an unknown sample coming from any other of the studied countries would be hardly identified. Is this actually the case? I suggest the authors to be clearer about the conditions under which the identification of an unknown sample is expected to work versus conditions under which the proposed method is likely to give erroneous predictions.
Response:
In lines 292-330, we added the new section "Identification of unknown samples". In this new section, there is information about accuracy of the identification. Apart from leave-one-out cross-validation, we used as unknown samples data from Nawrocka et al. (2018), which were recently uploaded to Zenodo:
https://doi.org/10.5281/zenodo.7567336.
In lines 390-432, we added discussion related to this problem.

Other comments:
1/ R script
I have run all the R script. It is tidy, well annotated, and easy to follow. It works very well except in three following cases that need to be corrected:
sample.error <- sample.class[sample.class$error == 'error',
should be
sample.error <- sample.class[sample.class$error == "err\nor"
geo.dist <- distm(country.geo.data[c('longitude','latitude')], fun = distGeo)
should be
geo.dist <- distm(country.geo.data[c('longitude\n','latitude')], fun = distGeo)
Response:
The corrections are not clear to us. The first one can be related to the use of single or double quote. The two ways to delimit characters can be used interchangeably. We have changed all single quotes to double quotes.
In both corrections, the character \n was added. This was probably by accident because when this character is added the script does not work anymore.

Suuplementary file “unknown wings”: Remove all the “” from the dataset as it creates errors upon file importation in R.

Response:
The supplementary file “unknown.csv” was removed from the revised version. In the current version all csv files are without quotation marks.

2/ ‘Introduction’ and ‘Discussion’ sections need some rewriting to focus on the points to which the dataset is designed for. Sometimes I felt the reader get lost in details which are not so relevant.
Response:
We have removed from the discussion the parts that we feel are not essential.
We have added subheadings.

3/ Methods :
*It will help if the authors provide more details about the sampling conditions:
- How were bees captured ?
- How were individuals conserved before their wing was imaged ?
- How were wings mounted and imaged ? Provide imaging conditions + mounting procedure.
Response:
In lines 167-170 and 189-192, we have added more information about sample collection, wing mounting, and image acquisition. The samples originated from different studies and differed to some degree in terms of the methodology used. This is one of the factors that reduces repeatability. However, in the future, the image acquisition methodology for unknown samples will also vary between studies.

*Could the authors provide an actual picture of a wing with the landmarks positions (ref 43 only provides a scheme) ?
Response:
We added Fig. 2 with landmarks marked on the wing image.

*Line 228 : define CVA
Response:
We defined CVA in line 220.

*Line 228 : clarify what is meant by “appropriate transformation”
Response:
We have removed the expression “appropriate transformation” as it is not necessary.

4/ Results :
* If wing shape changes are noticeable, could the authors provide a figure showing how is wing shape varying geographically and/or between evolutionary lineages ? For example, in figure 2, what is the change in landmarks positions from a wing having low values along PC1 (e.g., Spain/Portugal), compared to a wing having high values along PC1 ? (e.g., Greece). Same for PC2. Alternatively, if relevant, this information could be provided for CV1 and CV2 in Figure 4. Practically speaking, this can be obtained with the function plotRefToTarget in R.
Response:
In Fig. 3, we added wireframes graphs (Fig. 3B, 3C), which indicate the shape change along PC1 and PC2. The wireframes graphs in case of CV1 and CV2 would be very similar to those representing PC1 and PC2, therefore, they were not added.

* Visualisation of lineages : I believe the plots obtained from the R code (at section “# Projection of the samples to canonical variate space from Nawrocka et al. 2018” and at the section “Classification of the samples to lineages according to Nawrocka et al. 2018”) are complementary to what is shown in figure 7 of the manuscript to visualise the relationship between the geographical variation and the evolutionary lineages. I suggest these plots to be included as main or supplementary figure.
Response:
We added Figs. S1 and S2. Because there is already 8 figures in the main text, we added them to the supplementary materials.

*Figures 2 and 4. Do ellipse represent 95% confidence interval of the mean ? If so specify in figure legend.
Response:
We added the information about the ellipses in figure captions (lines 818 and 828).

Powered by Editorial Manager® and ProduXion Manager® from Aries Systems Corporation

	*Line 276 : Since using less than 25 wings/sample leads to misclassifications, should it be a recommendation to use at least 25 wings/sample ? Would "unknown samples "containing less than 25 wings be misclassified ? This technical aspect might be worth mentioning in the discussion.<br>Response:<br>We explained that the identification can be based on one sample, as long as it consists of at least 10 wings (lines 433-438).
**Additional Information:**	
**Question**	**Response**
Are you submitting this manuscript to a special series or article collection?	No
**Experimental design and statistics**<br><br>Full details of the experimental design and statistical methods used should be given in the Methods section, as detailed in our Minimum Standards Reporting Checklist. Information essential to interpreting the data presented should be made available in the figure legends.<br><br>Have you included all the information requested in your manuscript?	Yes
**Resources**<br><br>A description of all resources used, including antibodies, cell lines, animals and software tools, with enough information to allow them to be uniquely identified, should be included in the Methods section. Authors are strongly encouraged to cite Research Resource Identifiers (RRIDs) for antibodies, model organisms and tools, where possible.<br><br>Have you included the information requested as detailed in our Minimum Standards Reporting Checklist?	Yes
**Availability of data and materials**<br><br>All datasets and code on which the conclusions of the paper rely must be either included in your submission or	Yes

deposited in publicly available repositories (where available and ethically appropriate), referencing such data using a unique identifier in the references and in the "Availability of Data and Materials" section of your manuscript.<br><br>Have you have met the above requirement as detailed in our Minimum Standards Reporting Checklist?	

Manuscript
Click here to access/download;Manuscript;Apis-mellifera-wings-ed R1-AT-SCE.DOCX

Honey bee (*Apis mellifera*) wing images: a tool for identification and conservation

Andrzej Oleksa, Department of Genetics, Faculty of Biological Sciences, Kazimierz Wielki University, Powstańców Wielkopolskich 10, 85-090 Bydgoszcz, Poland, olek@ukw.edu.pl

Eliza Căuia, Adrian Siceanu, Honeybee Genetics and Breeding Laboratory, Institute for Beekeeping Research and Development, Blv Ficusului, no. 42, Sector 1, 013975 Bucharest, Romania, eliza.cauia@yahoo.com, siceanu.adrian@gmail.com

Zlatko Puškadija, Marin Kovačić, Faculty of Agrobiotechnical Sciences, Josip Juraj Strossmayer University of Osijek, Osijek, Croatia, zlatko.puskadija@fazos.hr, Marin.Kovacic@fazos.hr

M. Alice Pinto, Centro de Investigação de Montanha, Instituto Politécnico de Bragança, Campus de Santa Apolónia, 5300-253, Bragança, Portugal, apinto@ipb.pt

Laboratório Associado para a Sustentabilidade e Tecnologia em Regiões de Montanha (SusTEC), Instituto Politécnico de Bragança, Campus de Santa Apolónia, 5300-253 Bragança, Portugal

Pedro João Rodrigues, Centre in Digitalization and Intelligent Robotics, Instituto Politécnico de Bragança, Campus de Santa Apolónia, 5300-253, Bragança, Portugal, pjsr@ipb.pt

Laboratório Associado para a Sustentabilidade e Tecnologia em Regiões de Montanha (SusTEC), Instituto Politécnico de Bragança, Campus de Santa Apolónia, 5300-253 Bragança, Portugal

Fani Hatjina, Leonidas Charistos, Department of Apiculture, Institute of Animal Science - Ellinikos Georgikos Organismos 'DIMITRA', 63200 Nea Moudania, Greece, fhatjina@elgo.gr, leocharistos@elgo.gr

Maria Bouga, Lab of Agricultural Zoology and Entomology, Agricultural University of Athens, 11855 Athens, Greece, mbouga@aua.gr

Janez Prešern, Agricultural Institute of Slovenia, Ljubljana, Slovenia, janez.presern@kis.si

İrfan Kandemir, Ankara University, Department of Biology, Faculty of Science, Ankara University, Beşevler-Ankara, Türkiye, ikandemir@gmail.com

Slađan Rašić, Faculty of Ecological Agriculture, EDUCONS University, Vojvode Putnika 87, 21208 Sremska Kamenica, Serbia, rasic.sladjan@gmail.com

Szilvia Kusza, Centre for Agricultural Genomics and Biotechnology, University of Debrecen, 4032 Debrecen, Hungary, kusza@agr.unideb.hu

*Adam Tofilski, Department of Zoology and Animal Welfare, University of Agriculture in Krakow, Krakow, Poland, rotofils@cyf-kr.edu.pl

*Corresponding author: Adam Tofilski

ORCID iDs: Andrzej Oleksa [0000-0002-0414-8075]; Eliza Căuia [0000-0002-4476-4411]; Adrian Siceanu [0000-0003-1421-4141]; Zlatko Puškadija [0000-0001-9613-3251]; Marin Kovačić [0000-0002-3782-6733]; Maria Alice Pinto [0000-0001-9663-8399]; Pedro João Rodrigues [0000-0002-0555-2029]; Fani Hatjina [0000-0001-6506-5874]; Leonidas Charistos [0000-0002-1279-1300]; Maria Bouga [0000-0001-7493-9158]; Janez Prešern [0000-0003-2479-6106]; Irfan Kandemir [0000-0002-2888-1044]; Slađan Rašić [0000-0001-6859-2784]; Szilvia Kusza [0000-0002-5441-5303]; Adam Tofilski [0000-0002-3898-7029];

Abstract

Background:

The honey bee (*Apis mellifera*) is an ecologically and economically important species that provides pollination services to natural and agricultural systems. The biodiversity of the honey bee in parts of its native range is endangered by migratory beekeeping and commercial breeding. In consequence, some honey bee populations that are well adapted to the local environment are threatened with extinction. A crucial step for the protection of honey bee biodiversity is reliable differentiation between native and non-native bees. One of the methods that can be used for this is the geometric morphometrics of wings. This method is fast, low-cost, and does not require expensive equipment. Therefore, it can be easily used by both scientists and beekeepers. However, wing geometric morphometrics is challenging due to the lack of reference data that can be reliably used for comparisons between different geographic regions.

Findings:

Here, we provide an unprecedented collection of 26,481 honey bee wing images representing 1,725 samples from 13 European countries. The wing images are accompanied by the coordinates of 19 landmarks and the geographic coordinates of the sampling locations. We present an R script that describes the workflow for analysing the data and identifying an unknown sample. We compared the data with available reference samples for lineage and found general agreement with them.

Conclusions:

The extensive collection of wing images available on the Zenodo website can be used to identify the geographic origin of unknown samples and therefore assist in the monitoring and conservation of honey bee biodiversity in Europe.

Keywords: honey bee, *Apis mellifera*, biodiversity, conservation, wing, geometric morphometrics

Data description

We provide 26,481 forewing images of honey bee workers. They represent 1,725 samples from 13 European countries (Table 1, Fig. 1). The shape of the wings was described using the coordinates for 19 landmarks at wing veins' intersections (Fig. 2). The whole dataset, including the wing images, landmark coordinates, geographic coordinates of sampling locations, and other data, is available on the Zenodo website [1] under a Public Domain licence.

Introduction

Honey bees (*Apis mellifera*, NCBI:txid7460) are ecologically and economically important. Their value as pollinators of wild plants and crops is much greater than the economical return from honey or other products of the beekeeping industry [2]. At the same time, in the USA [3] and some European countries [4,5] there has been a decline in the number of managed honey bee colonies. In addition to pathogens, pesticides [6] and socioeconomic factors [7], loss of genetic variability is considered one of the possible causes of such decline [8–10].

The honey bee native distribution covers Europe, up to a latitude of about 60 degrees north, Africa, the Middle East [11], and Central Asia [12,13]. Within this wide range, the environment varies markedly. Such diverse environmental conditions, as well as the history of range expansion and isolation of populations, have resulted in notable variation of morphological and behavioural traits, as represented by more than 24 subspecies

(geographical races) [11–18]. The subspecies were initially grouped into four evolutionary lineages (A, C, M and O [11]) from morphological data, but recently three additional lineages (Y, L, and U) were identified from molecular data [19,20].

The biodiversity of the honey bee is becoming increasingly endangered by the mass introduction of queens produced by breeding. This process began in the 19th century [21] and has intensified recently [22,23]. The honey bee queens used by many beekeepers are the daughters of relatively few selected breeder queens [24], which are often hybrids [25]. In some European countries, more than 15% of all colonies are re-queened every year [26]. In consequence, some populations, which are well adapted to the local environment [27], are threatened by introgressive hybridization [22]. In this context, it is important to distinguish native honey bees, which occurred in a particular area before the intensification of beekeeping, from non-native ones, introduced into the area by human intervention.

The conservation of honey bee subspecies requires their identification, which may be based on molecular markers. Recently, identification methods have been developed from single nucleotide polymorphisms (SNPs) [28–30], which are more accurate than microsatellites [31]. The problem is that, in spite of the decreasing costs of SNP genotyping [29], molecular identification is still expensive and is not easily and quickly accessible to beekeepers. A cheaper alternative is the identification of subspecies based on wing venation measurements [11,18,32]. The wings can be measured using various methodologies based on distances and angles [33], landmark coordinates [34–36], outlines [37], or image pixels [38]. While it has been demonstrated that morphological and molecular markers might provide similar results [39,40, but see also 41], identification always requires a reference dataset, which is often inadequate.

Public data suitable for the identification of honey bee subspecies or evolutionary lineages using wing morphometry is scarce. Early studies based on multiple measurements of wings provided averages and standard deviations for all measured distances and angles, as well as details related to linear discriminant analysis (LDA) [42,43]. Unfortunately, in later studies, including those covering global honey bee diversity, averages were not provided [33] or were provided only for a few selected variables [11]. For example, in a study of *Apis mellifera mellifera* only 9 out of 36 characteristics were reported [11: Table 13.1]; among them, there are no data for the venation angles used for discrimination of this subspecies [11: page 229]. More importantly, the details of LDA were often not presented in those studies, preventing readers from using them to identify unknown samples. Later, the LDA details were provided in a few studies [34,36,44–46]. However, despite this progress, it would be even more useful to give readers access to all of the raw data used in the analysis instead of providing LDA details, as has been done in some studies related to wing measurements in Diptera [47–49]. In the case of honey bees, only one study is known to us in which the landmark data were made available [50]. Providing landmark coordinates solves the problem of data availability only partially because different studies may use different configurations of landmarks [18]. Not only can the order of landmarks differ between studies, but their positions may also vary. This makes comparisons between studies difficult or even impossible. On the other hand, if a wing image is available, it can be re-analysed, and missing or incompatible landmarks can be determined. In addition, the wing images can be used to determine the landmarks automatically [35,51,52].

Currently, while wing measurements are usually based on images, they are rarely made available after publication. This applies not only to honey bees but also to other insects. Usually, only one image is presented with information about the position of the landmarks [47]. There are only a few examples in which wing images have been made publicly available. This is the case of one study on Vespidae [53] and one study on *Drosophila*

melanogaster, for which a large repository of wing images was recently provided [54]. Honey bee wing images have never been made available in such significant numbers.

The lack of reference data on the morphological variation of honey bees can be alleviated by data sharing, which is common practice in some scientific fields, including genomics [55] and neurosciences [56]. It is one of the factors that have facilitated the rapid growth of those fields in recent years. In contrast to molecular biology, data sharing is relatively rare in ecological studies [57]. The benefits of data sharing within the scientific community are well known. However, individual authors often resist making their data available [58–60]. Even if some data are provided, they are often incomplete [61]. Large-scale investigations require large datasets [62], which are difficult for a single researcher to obtain. Individual studies on honey bee biogeography often focus on a relatively small area of one or a few countries, and large-scale comparisons are rare [32,39,45]. Data sharing would allow the combining of datasets from multiple studies to obtain better knowledge about large-scale geographic variation and the conservation status of honey bee subspecies.

In an attempt to begin building a global reference dataset for the honey bee, here we provide an extensive repository of wing images representative of its diversity in a large tract of Europe. The wing images are accompanied by the coordinates of 19 landmarks, which can easily be used in future comparisons. We also present an R script in which we analyse the coordinates and show how they can be used to identify the origin of an unknown sample. Among other applications, the repository can be used to identify native honey bees, which is essential for their conservation.

Methods

Material

In this study, we used 26,481 forewing images of honey bee workers (non-reproductive diploid females). They represent 1,725 samples from 13 European countries (Table 1, Fig. 1). A sample consists of workers that were collected either from one colony or from flowers in one location. In the case of samples collected from colonies, the workers were swept with a brush from a middle comb. In the case of samples collected from flowers, the workers were captured using an entomological net. After collection, the workers were stored in alcohol until their preparation. The number of workers per sample ranged from 5 to 20. In some cases (Poland and Hungary), when only one or two workers were collected from one location, neighbouring locations were treated together to obtain at least ten wings per sample. The samples from Austria, Montenegro, and Serbia were obtained from queen breeders who use artificial selection. While breeding lines in Austria were maintained through instrumental insemination of queens, queen bees in Montenegro and Serbia were open-mated. Country names are abbreviated according to ISO 3166-1 (Table 1). The geographic coordinates of the samples, the year of their collection, and other information are provided in CSV files for each country separately.

Table 1. Sample size of honey bee wings used in this study.

Country	Country abbreviation	Number of wings	Number of samples
Austria	AT	198	10
Croatia	HR	6103	160
Greece	GR	1444	244
Hungary	HU	426	22
Moldova	MD	263	10
Montenegro	ME	300	20

Poland	PL	5955	253
Portugal	PT	960	192
Romania	RO	6498	197
Serbia	RS	299	20
Slovenia	SI	835	21
Spain	ES	2563	516
Turkey	TR	637	60

Some of the samples were analysed in earlier studies addressing other goals: Croatia and Slovenia [63], Greece [64,65], Hungary and Poland in part [66,67], Portugal and Spain [39,41], Romania [68], Serbia and Montenegro [69], and Turkey [70]. Those studies used various methods, and their results could not be directly compared. Therefore, most of the wings were re-measured for the analyses performed herein. In none of the earlier studies were the wing images made publicly available. The data from Austria and Moldova had never been analysed or published before. Wing preparation and image acquisition differed between the studies. In most cases, the wings were detached from the bees' bodies and then mounted between two microscopic slides. For image acquisition, different types of cameras combined with stereo microscopes [39,64,65,69,70] or macro lenses [63,66–68,68] were used.

Each wing from the dataset was saved as a separate PNG file. The wing image file name begins with the two-letter country code (Table 1), followed by a hyphen (identical to the minus sign), a four-digit sample code, another hyphen, and finally the original file name. The original file names vary as they originate from various studies. They usually consist of strings separated by hyphens. In some cases, the name ends with a letter L or R indicating left or right wing. The samples were independently numbered in each country; for this reason, the unique sample name has to include the two-letter country code. The wing images were sorted by country and compressed into 13 ZIP files.

In each wing image, the coordinates of 19 landmarks were determined (Fig. 2) [45]. The landmarks are compatible with the "standard honey bee morphometry" approach used in earlier studies [33], and the landmark coordinates can be converted to distances and angles [as in 63]. The landmarks were saved within each wing image file and can be viewed and edited in the IdentiFly software application [45]. The raw coordinates of the landmarks were saved in CSV files for each country separately. The whole dataset, including the 26,481 forewing images, landmark coordinates, geographic coordinates of sampling locations, and other data, is available on the Zenodo website [1] under a Public Domain licence.

Statistical analysis

The statistical analysis was performed in R (v. 4.0.3) [71] using RStudio (v. 2022.12.0, RRID:SCR_000432). All details of the statistical analysis are available as supplementary data (Supplementary Document 1). Landmark coordinates from all wings were superimposed using generalised Procrustes analysis in the geomorph package (v. 4.0.4) [72]. The aligned coordinates were averaged within samples, and the averages were used in the subsequent analysis. Principal component analysis was used to extract the first two principal components, which were used to describe how the wing shape varied geographically. The association between the principal components and geographic coordinates (latitude and longitude) was analysed using generalised additive model (GAM) regression in the mgcv package (v. 1.8-33) [73]. Canonical variate analysis (CVA) and the differences between countries and regions were calculated using the Morpho package (v. 2.9) [74]. The wing shape was clustered using the unweighted pair group method with arithmetic mean (UPGMA) in the phangorn package (v. 2.5.5) [75]. The differences in wing shape between countries or regions were described using Mahalanobis distance. The correlation between the Mahalanobis distances and

geographical distances was analysed using the Mantel test. The coordinates of the unknown samples obtained from Nawrocka et al. [45,76] were aligned using generalised Procrustes analysis and averaged within samples. Next, the samples were aligned with a consensus of the reference sample using ordinary Procrustes analysis in the shapes package (v. 1.2.6) [77]. Finally, the CVA scores of unknown samples were obtained and compared with the CVA scores of reference samples to calculate their probabilities of belonging to each country or region. The workflow of the identification procedure is described in the supplementary materials (Supplementary Document 1), also available at the WorkflowHub website [78].

Results

Exploratory data analysis

The wing shape varied significantly according to principal component analysis. In the graph of the first two principal components, which account for 51.2% and 8.6% of the variance, respectively, at least two clear clusters of points are visible (Fig. 3). One of the clusters represents the Iberian Peninsula, and the other represents Central and South-Eastern Europe. The second principal component differentiated Greece from the countries of Central Europe, particularly Austria. The wing shape variation was strongly correlated with geographic location. Latitude and longitude correlated significantly with both the first and second principal components (GAM regression, PC1: EDF = 26.18, F = 720.7, $P < 10^{-15}$; PC2: EDF = 27.86, F = 58.47, $P < 10^{-15}$). The first principal component was much lower in the Iberian Peninsula than in the Balkans (Fig. 4A). Additionally, it decreased in Poland from south to north and in Greece from west to east (Fig. 4A). The second principal component was lowest in south-eastern Greece, increasing towards the north and west, with some intricate patterns in Romania and Poland (Fig. 4B).

As expected, canonical variate analysis revealed deeper differences between countries and showed a similar pattern to principal component analysis (Fig. 5). The shape of honey bee wings (represented by 34 principal components) differed significantly among countries (MANOVA: F = 22.1, $P < 10^{-15}$). In pair-wise comparisons, most countries differed markedly from each other. Only Romania did not differ significantly from Moldova, Serbia from Montenegro, and Slovenia from Croatia and Hungary (Table 2). The largest Mahalanobis distance was found between samples from Portugal and Greece, and the smallest between samples from Portugal and Spain. The UPGMA tree shows more details about the similarities between the wings collected from different countries (Fig. 6). Most neighbouring countries cluster together: Portugal with Spain, Greece with Turkey, Moldova with Romania, and Slovenia with Croatia. Isolation by distance was confirmed by a significant positive correlation between geographic distances and Mahalanobis distances of wing shape between countries (Mantel test: r = 0.7046, P = 0.0015). The Austrian samples did not fit well into this relationship. Despite their close geographic proximity to Slovenia, Croatia, and Hungary, they had unexpectedly different wing shapes (Fig. 7).

Table 2. Differences between countries in wing shape (expressed as Mahalanobis distances, lower triangle) and significance of pair-wise comparisons (upper triangle). For country abbreviations, see Table 1.

country	AT	ES	GR	HR	HU	MD	ME	PL	PT	RO	RS	SI	TR
AT	-	0.0001	0.0001	0.0001	0.0026	0.0009	0.0002	0.0001	0.0001	0.0001	0.0005	0.0001	0.0001
ES	10.3602	-	0.0001	0.0001	0.0001	0.0001	0.0001	0.0001	0.0001	0.0001	0.0001	0.0001	0.0001
GR	9.6709	11.3111	-	0.0001	0.0001	0.0001	0.0001	0.0001	0.0001	0.0001	0.0001	0.0001	0.0001
HR	7.4676	11.1342	5.2491	-	0.0250	0.0083	0.0001	0.0001	0.0001	0.0001	0.0001	0.0933	0.0001

HU	6.3694	10.9745	6.6387	2.9550	-	0.0155	0.0003	0.0011	0.0001	0.0025	0.0017	0.0524	0.0002
MD	7.4600	10.5178	6.7506	4.6274	5.1587	-	0.0059	0.0072	0.0001	0.0669	0.0160	0.0404	0.0012
ME	6.9289	10.5056	6.4158	5.2746	5.7927	5.8540	-	0.0001	0.0001	0.0001	0.8670	0.0001	0.0001
PL	6.9864	9.2408	6.2533	3.9108	3.9095	4.6505	6.2075	-	0.0001	0.0001	0.0001	0.0004	0.0001
PT	10.2711	1.8918	11.4833	11.2249	10.9754	10.3770	10.4285	9.1929	-	0.0001	0.0001	0.0001	0.0001
RO	6.3672	10.5980	5.8505	3.7678	3.8011	3.6076	4.8826	4.4288	10.5648	-	0.0003	0.0003	0.0001
RS	7.0790	10.7933	6.1816	4.7181	5.3638	5.2334	1.8720	5.8124	10.7111	4.5827	-	0.0002	0.0001
SI	8.0962	11.4743	5.9944	2.4516	3.5920	4.6343	6.5067	4.1484	11.4397	4.2440	5.8863	-	0.0001
TR	7.7594	10.2036	4.9768	4.3165	5.2857	5.7750	6.0269	5.1654	10.3717	4.3490	6.0440	5.1704	-

Classification of samples as lineages

When the samples were classified as lineages (Fig. S1, S2), using the data from Nawrocka et al. [45], many of them (n = 844, 48.9%) were classified as lineage C, which occurred in all samples from six countries: Austria, Croatia, Hungary, Montenegro, Serbia, and Slovenia. Lineage C was also the most frequent one in Greece, Moldova, Poland, Romania, and Turkey (Fig. S2). As expected, the samples most similar to lineage C occurred in south-eastern Europe, except in south-eastern Greece (Fig. 8B).

The second most frequent was lineage M. It occurred in 652 samples (37.8%). It was dominant in Portugal and Spain but also occurred in Poland (Fig. S2). A clear similarity to lineage M was observed in the Iberian Peninsula. Moreover, similarity to this lineage increased in Poland from south to north (Fig. 8C).

Unexpectedly, a relatively large fraction of the samples (n = 179, 10.4%) was classified as lineage A. It was detected mainly in Poland, but also in the Iberian Peninsula, Greece, Turkey, and Moldova at lower proportions (Fig. S2). Similarity to lineage A increased in Poland from south to north and in Greece from north-west to south-east (Fig. 8A).

Lineage O was the least frequent (n = 50, 2.9%). It occurred in Greece and sporadically in Moldova, Poland, Romania, and the European part of Turkey (Fig. S2). Similarity to lineage O increased in Greece from the north-west to the south-east (Fig. 8D).

Identification of unknown samples

The data provided here can be used for the identification of an unknown sample of honey bee workers. To test the accuracy of such identification, the leave-one-out cross-validation approach was used. This was based on samples and not on single wing measurements, in order to increase accuracy (for more information, see the discussion). One sample was temporally removed from the dataset, which was subject to canonical variate analysis, and the obtained data were used to classify the newly removed sample. In this procedure, the removed sample was treated as unknown and the remaining data as reference. The cross-validation was repeated for all samples, and the percentage of correctly classified samples was calculated. When the samples were classified (with cross-validation) according to their country of origin, 86.26% of them were assigned to the correct group. Misclassifications most often occurred between neighbouring countries. For example, the correct classification rate for Portugal was only 79.69%, with all the cases of misclassification occurring with the neighbouring Spain. Many misclassifications can be attributed to the small sample size for some countries; therefore, the second classification was based on regions. Samples from some smaller countries were combined with those from their large neighbours (Portugal with Spain, Moldova with Romania, and Slovenia with Croatia), and other countries with sample sizes below 25 were excluded (Austria, Hungary, Montenegro, and Serbia). In the case of classification to regions, the correct classification rate (with cross-validation) increased to 98.31%.

Additionally, we classified to regions an independent dataset of historical data from Nawrocka et al. [45,76]. In the analysis we used a subset consisting of 53 colonies originating from Europe (lineage C and M). Within this subset there was 18 colonies which originated from countries represented in the regions reference data. Among them, a single sample from Croatia was correctly classified as belonging to region HR-SI; out of 11 samples from Greece three were correctly classified, and eight were classified as belonging to the neighbouring Turkey and the region HR-SI; two samples from Romania were incorrectly classified as belonging to region HR-SI; one sample from Slovenia was correctly classified as belonging to region HR-SI and the other was incorrectly classified as belonging to Poland; finally, two samples from Spain were correctly classified.

The reference samples provided here cover only part of Europe; therefore, identification of samples from other parts of Europe and the world can give unexpected results. Often, such samples can be detected as outliers, which have low identification probabilities for all groups. Here, we used an arbitrary threshold value of 0.001; if the maximum probability of identification for a sample is lower, we assume it is an outlier. Unfortunately, many of the identified samples, which originated from countries not covered by reference data, were classified as coming from one of the regions with a relatively high probability. For example, most samples from Italy (representing *A. m. ligustica*) were classified as belonging to either Poland or HR-SI with a probability above 0.001.

Discussion

Comparison with earlier studies

The data presented here show that the geographic variation of honey bee wing shape in Europe is still large. This variation is most likely an effect of natural selection and not of beekeepers' mass introduction of non-native bees. When compared with historical reference samples from the Morphometric Bee Data Bank in Oberursel [11], the bees analysed here fit well into the pattern, which is believed to have been shaped by natural processes. In particular, there is a high similarity to lineage M in the Iberian Peninsula and in the north of Poland, and there is a high similarity to lineage C in most of Central and South-Eastern Europe, as expected from the seminal work on honey bee taxonomy and biogeography of F. Ruttner [11]. In the eastern part of the Aegean Sea, there is a high similarity to lineage O, which occurred naturally in Turkey and Middle East. In general, the distribution of the lineages presented in this study is in line with earlier studies based on morphometry [33], mitochondrial DNA [79,80], microsatellites [80–82], and single nucleotide polymorphisms [30,83]. On the other hand, there are some discrepancies, which are discussed below.

It can be expected that the introduction of non-native bees will reduce geographic variation because beekeepers prefer certain honey bee subspecies (*A. m. carnica*, *A. m. ligustica*, *A. m. caucasia*) or their hybrids [22]. Beekeepers' preference for a limited number of breeding lines [24] may lead to a significant homogenisation of the population structure across Europe. It is worth noting, however, that despite the increased beekeeper-mediated gene flow in the last decades [22], our results indicate that native honey bee diversity is still not lost, at least in eastern and southern Europe. The population size of honey bees is very large [approximately 19 million colonies - 84]; hence, it may take many generations to change the genetic structure and thus the phenotype of the European population. Free trade in breeding material has intensified only in the last several decades; hence, homogenisation of population structure may occur in the future, unless remedial measures are taken to protect local genetic variability.

Some of the results from our study do not agree with the patterns reported by F. Ruttner [11]. Many colonies in northern Poland and some other countries were classified as

belonging to lineage A. This lineage is expected on the African continent and does not occur naturally in central and northern Europe [11]. One possible explanation for the presence of this lineage in Europe is human-induced introgression. A recent mitochondrial DNA survey in Central Europe detected haplotypes of African ancestry, although with a frequency of only 1.64% [66], which is much lower than that reported here for the forewing samples from Poland classified according to Nawrocka et al. [45] as lineage A (38.3%). The high proportion of samples assigned to lineage A may also be related to the fact that hybrids between lineages are more likely to be classified as lineage A. Those hybrids have an intermediate phenotype [85], similar to the mean shape of all lineages.

While hybridisation may be caused by the introduction of non-native bees by beekeepers, hybrids between lineages can also occur naturally. Aside from the Alps, there is no physical barrier separating lineages M and C. In such a situation, a wide hybrid zone can be expected. Earlier studies reported that in Poland there is a wide transition zone with a clinal change in both morphological [86,87] and molecular [66] markers. This spatial pattern was most likely due to a natural phenomenon because it was already present in the 1960s [86], when the importation and rearing of non-native bees were less common.

Hybrids between lineages can be identified to some degree using wing measurements [41,85]. However, this requires adequate reference samples that are not currently available. The reference sample from the Morphometric Bee Data Bank [11] for lineage M consists of only 16 colonies, whereas those for lineages A, C, and O are larger, consisting of 85, 37, and 49 colonies, respectively [45]. The M reference sample is clearly small, especially when considering the very large native distribution of this lineage, which extends from Iberia to western China [12]. Thus, a large portion of M lineage variation is inevitably underrepresented in the reference dataset. The intriguing detection of lineage A in Europe needs further investigation to determine whether hybrids between lineages C and M are being incorrectly classified as lineage A or the African wing phenotypes are present in Europe more often than was previously expected.

Identification of unknown samples

When using the data provided here for the identification of an unknown sample, it is important to understand the limitations of the described methodology. If the unknown samples originate from one of the regions covered in the reference dataset, the results should be relatively accurate, i.e., the fraction of samples assigned to the correct region should be 98%. However, if the unknown samples originate from a region not covered in the reference dataset (e.g., Italy), they might be incorrectly classified as belonging to one of the regions or countries included in the identification model. In order to detect such misidentifications, the user should examine the probability of identification. If the probability is low, the unknown samples can be classified as outliers, as they will not match any of the geographical regions covered in the reference data. The threshold probability below which the samples are classified as outliers is arbitrary (here, we established a value of 0.001). Unfortunately, detection of outliers failed in many cases (e.g., in the case of samples from Italy). The lower identification rate of historic samples can be related to introgressive hybridization, which occurred in recent years in some parts of Europe [68]. The problem of false positives can be alleviated to some degree by adding contemporary samples from a wider geographic range to the identification model. The geographical coverage of Europe presented in this study is far from complete, as there are many countries without any data or with incomplete data. Among the countries included in this study, a better geographical coverage is warranted for Austria, Serbia, and Montenegro. The Austrian samples, which were obtained from a queen breeder, do not agree with the samples collected from the neighbouring countries. This is particularly evident in the isolation-by-distance plot, where they are clear outliers (Fig. 7). This

discrepancy may be related to artificial selection and drift, with the colonies being kept in genetic isolation from the surrounding population as a result of instrumental insemination. Ideally, reference samples should be collected from colonies in which queens are not sourced from breeding programmes and which are widespread in the study area to ensure coverage of the genetic variation in the population. Another possibility is collecting bees from flowers, in which case they represent multiple nearby colonies.

Despite the problems related to introgression and the presence of false positives that were mentioned above, the wing data presented in this study can be used as a reference for future studies aimed at the identification and monitoring of non-native honey bees. The wing shape of an unknown sample can be compared with a reference sample from a particular country or region. The identification results can be sorted by similarity to the reference, and colonies with the smallest similarity can be classified as non-native. These outlier colonies can be re-queened or removed from the population. In this context, the presence of false positives is not a big problem because this procedure is focused on the detection of true negatives. In fact, beekeepers should re-queen only a fraction of their colonies, and the procedure developed here is focused on the detection of the most extreme outliers. Moreover, it is not essential that the reference sample perfectly represents the native phenotype for a particular geographical region. The full range of original variation, present in Europe before large-scale movements of non-native honey bees, may be irreversibly lost. Fortunately, there is still considerable variation, and this variation deserves to be protected from further genetic erosion.

A large sample size of reference data should not be confused with the sample size of an unknown sample to be classified. In the latter case, one sample consisting of 10-20 workers from one colony or location should be sufficient. The identification may be based on only a single wing. However, in such cases, the results are inaccurate [36,88]. By averaging multiple wings within a sample, the measurement error and influence of environmental (and not genetic) factors are minimized.

The conservation of honey bee biodiversity is often focused on the protection of certain subspecies. For example, there are efforts to preserve *A. m. mellifera* [89]. These efforts are justified because in some parts of western and northern Europe, native honey bees are threatened with extinction due to introgressive hybridisation with non-native bees [90,91]. However, this approach can overlook some intra-specific variation. The subspecies concept oversimplifies the problem and attempts to classify a continuous variation into a categorical one. For example, different populations of *A. m. mellifera* within its wide range differ from each other much more than some subspecies do (e.g., *A. m. carnica* and *A. m. ligustica*) [30]. Relatively large intra-subspecific variation has been observed in *A. m. carnica*, *A. m. macedonica* [92], *A. m. iberiensis* [79,83,93], and *A. m. mellifera* [94]. Also, in Africa there is clinal variation, which makes the discrimination of subspecies difficult [95,96]. Continuous spatial variation is present even on the American continent, where the honey bee has been introduced [97]. In this study, we observed variation not only between countries, but also within them. In general, there is isolation by distance, with smaller differences between bees from neighbouring regions than between bees that are far apart [80,94]. We do not advocate abandoning the well-established concept of subspecies, but stress the importance of continuous variation, especially in the protection of honey bee diversity.

Conclusion

The problem of a lack of reference samples can be solved by data sharing. Here, we provide for the first time a large collection of honey bee forewing images, accompanied by geographic coordinates as well as measurements and some additional data [1]. The collection and metadata are easy to review, reuse and update. Moreover, we show how the data can be

analysed, for example, to predict the origin of an unknown sample. The dataset can be used, among other things, as a reference for future studies on biogeography and conservation of honey bees. We hope that future studies will also make wing images freely available in order to expand the dataset and improve our knowledge of honey bee geographic variation.

Data Availability

The whole dataset, including the wing images, landmark coordinates, geographic coordinates of sampling locations, and other data, is available on the Zenodo website [1] under a Public Domain licence. All details of the statistical analysis, including the identification of an unknown sample, are available as supplementary data (Supplementary Document 1), also available at the WorkflowHub website [78]. All supporting data and materials are available in the *GigaScience* GigaDB database [98].

Availability of source code and requirements

- Project name: *Apis mellifera* wing images from Europe
- Project home page: https://doi.org/10.5281/zenodo.7244070
- Operating system(s): Platform independent
- Programming language: R
- Other requirements: R (v. 4.0.3), numerous R packages described in the methods section and at the end of Supplementary Document 1
- License: Public Domain
- BiotoolsID: Apis-wings-EU
- WorkflowHub: https://doi.org/10.48546/WORKFLOWHUB.WORKFLOW.422.1

List of Abbreviations

AT: Austria, ES: Spain, GR: Greece, HR: Croatia, HU: Hungary, MD: Moldova, ME: Montenegro, PL: Poland, PT: Portugal, RO: Romania, RS: Serbia, SI: Slovenia, TR: Turkey, CVA: canonical variates analysis, LDA: linear discriminant analysis, GAM: generalised additive model, UPGMA: unweighted pair group method with arithmetic mean

Funding

This research was funded in part by the National Science Centre, Poland, grant numbers 2021/41/B/NZ9/03153 and 2015/19/B/NZ9/03718 and by the National Centre for Research and Development, grant number TANGO-V-A/0042/2021. M. Alice Pinto acknowledges Fundação para a Ciência e a Tecnologia for providing financial support from national funds (FCT/MCTES) to CIMO (UIDB/00690/2020 and UIDP/00690/2020) and SusTEC (LA/P/0007/2021). J Prešern acknowledges Slovenian Research Agency for providing funding from ARRS Research Program P4-0431 NextGenAgri.

Authors' contributions

AT and AO planned this study. All authors provided honey bee wing images and metadata from their countries. AT wrote the first version of the manuscript and all authors revised and contributed to its final version. All authors have read and approved the final manuscript.

Acknowledgements

Some samples from Poland were obtained within the framework of a project funded by the State Forests, "Pszczoły wracają do lasu" (Bees Return to the Forest), coordinated by Dr Kazimierz Szabla. Samples from Croatia were obtained during the project "Biodiversity of

the honey bee (*Apis mellifera carnica*) population in the Republic of Croatia (BioBeeCro)", funded by the Paying Agency for Agriculture, Fisheries and Rural Development. Samples from Portugal were obtained in the framework of the research project PTDC/BIA-BEC/099640/2008, funded FCT and COMPETE/QREN/EU. Samples from Moldova were obtained thanks to Dr Valentina Cebotari from Institute of Zoology, Academy of Sciences of Moldova, during Smartbees project (grant 613960 funded by Program EC FP7-KBBE). Bernadeta Rzeźnicka assisted with the wing measurements.

References

1. Oleksa A, Căuia E, Siceanu A, Puškadija Z, Kovačić M, Pinto MA, et al.. Collection of wing images for conservation of honey bees (*Apis mellifera*) biodiversity in Europe. *Zenodo*. 2022; doi: 10.5281/zenodo.7244070.

2. Gallai N, Salles J-M, Settele J, Vaissière BE. Economic valuation of the vulnerability of world agriculture confronted with pollinator decline. *Ecological Economics*. 2009;68:810–21. doi: 10.1016/j.ecolecon.2008.06.014.

3. Ellis JD, Evans JD, Pettis J. Colony losses, managed colony population decline, and Colony Collapse Disorder in the United States. *Journal of Apicultural Research*. 2010;49:134–6. doi: 10.3896/IBRA.1.49.1.30.

4. Gray A, Brodschneider R, Adjlane N, Ballis A, Brusbardis V, Charrière J-D, et al.. Loss rates of honey bee colonies during winter 2017/18 in 36 countries participating in the COLOSS survey, including effects of forage sources. *Journal of Apicultural Research*. 2019;58:479–85. doi: 10.1080/00218839.2019.1615661.

5. Potts SG, Roberts SPM, Dean R, Marris G, Brown MA, Jones R, et al.. Declines of managed honey bees and beekeepers in Europe. *Journal of Apicultural Research*. 2010;49:15–22. doi: 10.3896/IBRA.1.49.1.02.

6. Steinhauer N, Kulhanek K, Antúnez K, Human H, Chantawannakul P, Chauzat M-P, et al.. Drivers of colony losses. *Current Opinion in Insect Science*. 2018;26:142–8. doi: 10.1016/j.cois.2018.02.004.

7. Moritz RFA, Erler S. Lost colonies found in a data mine: Global honey trade but not pests or pesticides as a major cause of regional honeybee colony declines. *Agriculture, Ecosystems & Environment*. 2016;216:44–50. doi: 10.1016/j.agee.2015.09.027.

8. Panziera D, Requier F, Chantawannakul P, Pirk CWW, Blacquiere T. The diversity decline in wild and managed honey bee populations urges for an integrated conservation approach. *Frontiers in Ecology and Evolution*. 2022; doi: 10.3389/fevo.2022.767950.

9. Themudo GE, Rey-Iglesia A, Robles Tascón L, Bruun Jensen A, da Fonseca RR, Campos PF. Declining genetic diversity of European honeybees along the twentieth century. *Sci Rep*. 2020;10:10520. doi: 10.1038/s41598-020-67370-2.

10. vanEngelsdorp D, Meixner MD. A historical review of managed honey bee populations in Europe and the United States and the factors that may affect them. *Journal of Invertebrate Pathology*. 2010;103:S80–95. doi: 10.1016/j.jip.2009.06.011.

11. Ruttner F. Biogeography and taxonomy of honeybees. Berlin: Springer, 1988. https://doi.org/10.1007/978-3-642-72649-1

12. Chen C, Liu Z, Pan Q, Chen X, Wang H, Guo H, et al.. Genomic analyses reveal demographic history and temperate adaptation of the newly discovered honey bee subspecies *Apis mellifera sinisxinyuan* n. ssp. *Mol Biol Evol*. 2016;33:1337–48. doi: 10.1093/molbev/msw017.

13. Sheppard WS, Meixner M. *Apis mellifera pomonella*, a new honey bee subspecies from Central Asia. *Apidologie*. 2003;34:367–75.

14. Bouga M, Alaux C, Bienkowska M, Büchler R, Carreck NL, Cauia E, et al.. A review of methods for discrimination of honey bee populations as applied to European beekeeping. *Journal of Apicultural Research*. 2011;50:51–84. doi: 10.3896/IBRA.1.50.1.06.

15. Engel MS. The taxonomy of recent and fossil honey bees (Hymenoptera : Apidae ; *Apis*). *Journal of Hymenoptera Research*. 1999;8:165–96.

16. Meixner MD, Costa C, Kryger P, Hatjina F, Bouga M, Ivanova E, et al.. Conserving diversity and vitality for honey bee breeding. *Journal of Apicultural Research*. 2010;49:85–92. doi: 10.3896/IBRA.1.49.1.12.

17. Meixner MD, Leta MA, Koeniger N, Fuchs S. The honey bees of Ethiopia represent a new subspecies of *Apis mellifera* — *Apis mellifera simensis* n. ssp. *Apidologie*. 2011;42:425–37. doi: 10.1007/s13592-011-0007-y.

18. Meixner MD, Pinto MA, Bouga M, Kryger P, Ivanova E, Fuchs S. Standard methods for characterising subspecies and ecotypes of *Apis mellifera*. *Journal of Apicultural Research*. 2013;52:1–28. doi: 10.3896/IBRA.1.52.4.05.

19. Dogantzis KA, Tiwari T, Conflitti IM, Dey A, Patch HM, Muli EM, et al.. Thrice out of Asia and the adaptive radiation of the western honey bee. *Science Advances*. American Association for the Advancement of Science, 2021;7:2151. doi: 10.1126/sciadv.abj2151.

20. Franck P, Garnery L, Loiseau A, Oldroyd BP, Hepburn HR, Solignac M, et al.. Genetic diversity of the honeybee in Africa: microsatellite and mitochondrial data. *Heredity*. 2001;86:420–30.

21. Crane E. The World History of Beekeeping and Honey Hunting. Routledge, 1999. ISBN: 9780415924672

22. De la Rúa P, Jaffé R, Dall'Olio R, Muñoz I, Serrano J. Biodiversity, conservation and current threats to European honeybees. *Apidologie*. 2009;40:263–84. doi: 10.1051/apido/2009027.

23. Nielsdatter MG, Larsen M, Nielsen LG, Nielsen MM, Rasmussen C. History of the displacement of the European dark bee (*Apis mellifera mellifera*) in Denmark. *Journal of Apicultural Research*. Taylor & Francis, 2021;60:13–8. doi: 10.1080/00218839.2020.1826111.

24. Schiff NM, Sheppard WS. Genetic analysis of commercial honey bees (Hymenoptera: Apidae) from the southern United States. *J Econ Entomol*. 1995;88:1216–20.

25. Lodesani M, Costa C. Bee breeding and genetics in Europe. *Bee World*. 2003;84:69–85.

26. Chauzat M-P, Cauquil L, Roy L, Franco S, Hendrikx P, Ribière-Chabert M. Demographics of the European Apicultural Industry. *PLOS ONE*. Public Library of Science, 2013;8:e79018. doi: 10.1371/journal.pone.0079018.

27. Büchler R, Costa C, Hatjina F, Andonov S, Meixner MD, Conte YL, et al.. The influence of genetic origin and its interaction with environmental effects on the survival of *Apis mellifera* L. colonies in Europe. *Journal of Apicultural Research*. 2014;53:205–14. doi: 10.3896/IBRA.1.53.2.03.

28. Chen C, Parejo M, Momeni J, Langa J, Nielsen RO, Shi W, et al.. Population Structure and Diversity in European Honey Bees (*Apis mellifera* L.) - An Empirical Comparison of Pool and Individual Whole-Genome Sequencing. *Genes*. Multidisciplinary Digital Publishing Institute, 2022;13:182. doi: 10.3390/genes13020182.

29. Henriques D, Browne KA, Barnett MW, Parejo M, Kryger P, Freeman TC, et al.. High sample throughput genotyping for estimating C-lineage introgression in the dark honeybee: an accurate and cost-effective SNP-based tool. *Sci Rep*. Nature Publishing Group, 2018;8:8552. doi: 10.1038/s41598-018-26932-1.

30. Momeni J, Parejo M, Nielsen RO, Langa J, Montes I, Papoutsis L, et al.. Authoritative subspecies diagnosis tool for European honey bees based on ancestry informative SNPs. *BMC Genomics*. 2021;22:101. doi: 10.1186/s12864-021-07379-7.

31. Muñoz I, Henriques D, Jara L, Johnston JS, Chávez-Galarza J, De La Rúa P, et al.. SNPs selected by information content outperform randomly selected microsatellite loci for delineating genetic identification and introgression in the endangered dark European honeybee (*Apis mellifera mellifera*). *Molecular Ecology Resources*. 2017;17:783–95. doi: 10.1111/1755-0998.12637.

32. Kandemir İ, Özkan A, Fuchs S. Reevaluation of honeybee (*Apis mellifera*) microtaxonomy: a geometric morphometric approach. *Apidologie*. 2011;42:618–27. doi: 10.1007/s13592-011-0063-3.

33. Ruttner F, Tassencourt L, Louveaux J. Biometrical-statistical analysis of the geographic variability of *Apis mellifera* L. *Apidologie*. 1978;9:363–81. doi: 10.1051/apido:19780408.

34. Francoy TM, Wittmann D, Drauschke M, Müller S, Steinhage V, Bezerra-Laure MA, et al.. Identification of Africanized honey bees through wing morphometrics: two fast and efficient procedures. *Apidologie*. 2008;39:488–94. doi: 10.1051/apido:2008028.

35. Rodrigues PJ, Gomes W, Pinto MA. DeepWings©: Automatic Wing Geometric Morphometrics Classification of Honey Bee (*Apis mellifera*) Subspecies Using Deep Learning for Detecting Landmarks. *Big Data and Cognitive Computing*. Multidisciplinary Digital Publishing Institute, 2022;6:70. doi: 10.3390/bdcc6030070.

36. Tofilski A. Using geometric morphometrics and standard morphometry to discriminate three honeybee subspecies. *Apidologie*. 2008;39:558–63. doi: 10.1051/apido:2008037.

37. Francoy TM, de Faria Franco F, Roubik DW. Integrated landmark and outline-based morphometric methods efficiently distinguish species of Euglossa (Hymenoptera, Apidae, Euglossini). *Apidologie*. 2012;43:609–17. doi: 10.1007/s13592-012-0132-2.

38. De Nart D, Costa C, Di Prisco G, Carpana E. Image recognition using convolutional neural networks for classification of honey bee subspecies. *Apidologie*. 2022;53:5. doi: 10.1007/s13592-022-00918-5.

39. Henriques D, Chávez-Galarza J, S. G. Teixeira J, Ferreira H, J. Neves C, Francoy TM, et al.. Wing Geometric Morphometrics of Workers and Drones and Single Nucleotide Polymorphisms Provide Similar Genetic Structure in the Iberian Honey Bee (*Apis mellifera iberiensis*). *Insects*. 2020;11:89. doi: 10.3390/insects11020089.

40. Oleksa A, Tofilski A. Wing geometric morphometrics and microsatellite analysis provide similar discrimination of honey bee subspecies. *Apidologie*. 2015;46:49–60. doi: 10.1007/s13592-014-0300-7.

41. García CAY, Rodrigues PJ, Tofilski A, Elen D, McCormak GP, Oleksa A, et al.. Using the Software DeepWings© to Classify Honey Bees across Europe through Wing Geometric Morphometrics. *Insects*. Multidisciplinary Digital Publishing Institute, 2022;13:1132. doi: 10.3390/insects13121132.

42. DuPraw EJ. The recognition and handling of honeybee specimens in non-Linnean taxonomy. *Journal of Apicultural Research*. 1965;4:71–84.

43. DuPraw EJ. Non-Linnean taxonomy and the systematics of honeybees. *Systematic Zoology*. 1965;14:1–24.

44. Bustamante T, Fuchs S, Grünewald B, Ellis JD. A geometric morphometric method and web application for identifying honey bee species (*Apis* spp.) using only forewings. *Apidologie*. 2021;52:697–706. doi: 10.1007/s13592-021-00857-7.

45. Nawrocka A, Kandemir İ, Fuchs S, Tofilski A. Computer software for identification of honey bee subspecies and evolutionary lineages. *Apidologie*. 2018;49:172–84. doi: 10.1007/s13592-017-0538-y.

46. Rinderer TE, Buco SM, Rubink WL, Daly HV, Stelzer JA, Riggio RM, et al.. Morphometric identification of Africanized and European honey bees using large reference populations. *Apidologie*. EDP Sciences, 1993;24:569–85. doi: 10.1051/apido:19930605.

47. López-García J, Angell C, Martín-Vega D. Wing morphometrics for the identification of Nearctic and Palaearctic Piophilidae (Diptera) of forensic relevance. *Forensic Science International*. 2020;309:110192. doi: 10.1016/j.forsciint.2020.110192.

48. Szpila K, Żmuda A, Akbarzadeh K, Tofilski A. Wing measurement can be used to identify European blow flies (Diptera: Calliphoridae) of forensic importance. *Forensic Science International*. 2019;296:1–8. doi: 10.1016/j.forsciint.2019.01.001.

49. Szpila K, Johnston NP, Akbarzadeh K, Richet R, Tofilski A. Wing measurements are a possible tool for the identification of European forensically important Sarcophagidae. *Forensic Science International*. 2022;340:111451. doi: 10.1016/j.forsciint.2022.111451.

50. Węgrzynowicz P, Łoś A. Dataset of wing venation measurements for *Apis mellifera caucasica*, *A. mellifera carnica* and *A. mellifera mellifera* (Hymenoptera: Apidae), their hybrids and backcrosses. *Biodivers Data J*. 2020;8:e53724. doi: 10.3897/BDJ.8.e53724.

51. Ángel-Beamonte E, Martín-Ramos P, Santolaria P, Sales E, Abizanda J, Yániz JL. Automatic determination of landmark coordinates for honey bee forewing venation using a new MATLAB-based tool. *Journal of Apicultural Research*. Taylor & Francis, 2018;57:605–10. doi: 10.1080/00218839.2018.1501856.

52. Batra SWT. Automatic image analysis for rapid identification of Africanized honey bees. In: Needham GR, editor. *Africanized honey bees and bee mites*. Ellis Horwood, p. 260–3.

53. Baracchi D, Dapporto L, Turillazzi S. Relevance of wing morphology in distinguishing and classifying genera and species of Stenogastrinae wasps. *Contributions to Zoology*. Brill, 2011;80:191–9. doi: 10.1163/18759866-08003003.

54. Sonnenschein A, VanderZee D, Pitchers WR, Chari S, Dworkin I. An image database of *Drosophila melanogaster* wings for phenomic and biometric analysis. *GigaScience*. 2015;4:s13742-015-0065–6. doi: 10.1186/s13742-015-0065-6.

55. Kaye J, Heeney C, Hawkins N, de Vries J, Boddington P. Data sharing in genomics — re-shaping scientific practice. *Nat Rev Genet*. Nature Publishing Group, 2009;10:331–5. doi: 10.1038/nrg2573.

56. Eglen SJ, Marwick B, Halchenko YO, Hanke M, Sufi S, Gleeson P, et al.. Toward standard practices for sharing computer code and programs in neuroscience. *Nat Neurosci*. Nature Publishing Group, 2017;20:770–3. doi: 10.1038/nn.4550.

57. Parr CS, Cummings MP. Data sharing in ecology and evolution. *Trends in ecology & evolution*. 2005;20:362–3.

58. Huang X, Hawkins BA, Lei F, Miller GL, Favret C, Zhang R, et al.. Willing or unwilling to share primary biodiversity data: results and implications of an international survey. *Conservation Letters*. 2012;5:399–406. doi: 10.1111/j.1755-263X.2012.00259.x.

59. Moles A, Dickie JB, Flores-Moreno H. A response to Poisot et al.: Publishing your dataset is not always virtuous. *Ideas in Ecology and Evolution*. 2013;6:20–2.

60. Soranno PA, Cheruvelil KS, Elliott KC, Montgomery GM. It's Good to Share: Why Environmental Scientists' Ethics Are Out of Date. *BioScience*. 2014;biu169. doi: 10.1093/biosci/biu169.

61. Roche DG, Kruuk LEB, Lanfear R, Binning SA. Public Data Archiving in Ecology and Evolution: How Well Are We Doing? *PLOS Biology*. Public Library of Science, 2015;13:e1002295. doi: 10.1371/journal.pbio.1002295.

62. Hampton SE, Strasser CA, Tewksbury JJ, Gram WK, Budden AE, Batcheller AL, et al.. Big data and the future of ecology. *Frontiers in Ecology and the Environment*. 2013;11:156–62.

63. Puškadija Z, Kovačić M, Raguž N, Lukić B, Prešern J, Tofilski A. Morphological diversity of Carniolan honey bee (*Apis mellifera carnica*) in Croatia and Slovenia. *Journal of Apicultural Research*. Taylor & Francis, 2020;60:326–36. doi: 10.1080/00218839.2020.1843847.

64. Bouga M, Hatjina F. Genetic variability in greek honey bee (*A. mellifera* L.) populations using geometric morphometrics analysis. *Proceedings of the Balkan scientific conference of biology in Plovdiv (Bulgaria)*. 2005;598–602.

65. Charistos L, Hatjina F, Bouga M, Mladenovic M, Maistros AD. Morphological Discrimination of Greek Honey Bee Populations Based on Geometric Morphometrics Analysis of Wing Shape. *Journal of Apicultural Science*. 2014;58:75–84. doi: 10.2478/jas-2014-0007.

66. Oleksa A, Kusza S, Tofilski A. Mitochondrial DNA Suggests the Introduction of Honeybees of African Ancestry to East-Central Europe. *Insects*. Multidisciplinary Digital Publishing Institute, 2021;12:410. doi: 10.3390/insects12050410.

67. Oleksa A, Tofilski A. Podgatunki pszczoły miodnej i rola lasów w ochronie ich różnorodności. *Ochrona owadów zapylających w ekosystemach leśnych (Eds Czekońska K, Szabla K)*. Krakow: Wydawnictwo Uniwersytetu Rolniczego w Krakowie,.

68. Tofilski A, Căuia E, Siceanu A, Vișan GO, Căuia D. Historical Changes in Honey Bee Wing Venation in Romania. *Insects*. Multidisciplinary Digital Publishing Institute, 2021;12:542. doi: 10.3390/insects12060542.

69. Rašić S, Mladenović M, Stanisavljević L. Use of geometric morphometrics to differentiate selected lines of Carniolan honeybees (*Apis mellifera carnica*) in Serbia and Montenegro. *Archives of Biological Sciences*. 2015;67:929–34.

70. Çakmak İ, Fuchs S, Çakmak SS, Özkan Koca A, Nentchev P, Kandemir İ. Morphometric Analysis of Honeybees Distributed in Northern Turkey Along the Black Sea Coast. *Uludag Bee Journal*. Uludag University, Beekeeping Development Application & Research Center, 2014;14:59–68.

71. R Core Team. R: A Language and Environment for Statistical Computing. Vienna, Austria: R Foundation for Statistical Computing, 2018.

72. Baken EK, Collyer ML, Kaliontzopoulou A, Adams DC. geomorph v4.0 and gmShiny: Enhanced analytics and a new graphical interface for a comprehensive morphometric experience. *Methods in Ecology and Evolution*. 2021;12:2355–63. doi: 10.1111/2041-210X.13723.

73. Wood SN. Fast stable restricted maximum likelihood and marginal likelihood estimation of semiparametric generalized linear models. *Journal of the Royal Statistical Society: Series B (Statistical Methodology)*. 2011;73:3–36. doi: 10.1111/j.1467-9868.2010.00749.x.

74. Schlager S. Morpho and Rvcg – Shape Analysis in R: R-Packages for Geometric Morphometrics, Shape Analysis and Surface Manipulations. In: Zheng G, Li S, Székely G, editors. *Statistical Shape and Deformation Analysis*. Academic Press, p. 217–56.

75. Schliep KP. phangorn: phylogenetic analysis in R. *Bioinformatics*. 2011;27:592–3. doi: 10.1093/bioinformatics/btq706.

76. Nawrocka A, Kandemir İ, Fuchs S, Tofilski A. Dataset: Computer software for identification of honey bee subspecies and evolutionary lineages. *Apidologie*. 2018;49:172–84. doi: 10.5281/zenodo.7498540.

77. Dryden IL. shapes package. Vienna, Austria: R Foundation for Statistical Computing, 2021.

78. Oleksa A, Căuia E, Siceanu A, Puškadija Z, Kovačić M, Pinto MA, et al.. Apis-wings-EU: A workflow for morphometric identification of honey bees from Europe. WorkflowHub, 2023; doi: 10.48546/WORKFLOWHUB.WORKFLOW.422.1.

79. Chávez-Galarza J, Garnery L, Henriques D, Neves CJ, Loucif-Ayad W, Jonhston J. . S, et al.. Mitochondrial DNA variation of *Apis mellifera iberiensis*: further insights from a large-scale study using sequence data of the tRNAleu-cox2 intergenic region. *Apidologie*. 2017;48:533–44. doi: 10.1007/s13592-017-0498-2.

80. Franck P, Garnery L, Solignac M, Cornuet J-M. The Origin of West European Subspecies of Honeybees (*Apis mellifera*): New Insights from Microsatellite and Mitochondrial Data. *Evolution*. 1998;52:1119–34. doi: 10.1111/j.1558-5646.1998.tb01839.x.

81. Garnery L, Franck P, Baudry E, Vautrin D, Cornuet J-M, Solignac M. Genetic diversity of the west European honey bee (*Apis mellifera mellifera* and *A. m. iberia*). II. Microsatellite loci. *Genetics Selection Evolution*. 1998;30:S49.

82. Kandemir İ, Meixner MD, Ozkan A, Sheppard WS. Genetic characterization of honey bee (*Apis mellifera cypria*) populations in northern Cyprus. *Apidologie*. 2006;37:547–55. doi: 10.1051/apido:2006029.

83. Chávez-Galarza J, Henriques D, Johnston JS, Carneiro M, Rufino J, Patton JC, et al.. Revisiting the Iberian honey bee (*Apis mellifera iberiensis*) contact zone: maternal and genome-wide nuclear variations provide support for secondary contact from historical refugia. *Molecular Ecology*. 2015;24:2973–92. doi: 10.1111/mec.13223.

84. : Beekeeping sector: results of the pilot study on honey bee selection. https://agriculture.ec.europa.eu/news/beekeeping-sector-results-pilot-study-honey-bee-selection-2022-03-15_en Accessed 2023 Feb 2.

85. Węgrzynowicz P, Gerula D, Tofilski A, Panasiuk B, Bieńkowska M. Maternal inheritance in hybrids of three honey bee subspecies. *Journal of Apicultural Science*. 2019;63:131–8. doi: 10.2478/jas-2019-0010.

86. Bornus L, Demianowicz A, Gromisz M. Morfologiczne badania krajowej pszczoły miodnej. *Pszczelnicze Zeszyty Naukowe*. 1966;10:1–46.

87. Meixner MD, Worobik M, Wilde J, Fuchs S, Koeniger N. *Apis mellifera mellifera* in eastern Europe – morphometric variation and determination of its range limits. *Apidologie*. 2007;38:191–7. doi: 10.1051/apido:2006068.

88. Oleksa A, Wilde J, Tofilski A, Chybicki IJ. Partial reproductive isolation between European subspecies of honey bees. *Apidologie*. 2013;44:611–9. doi: 10.1007/s13592-013-0212-y.

89. Parejo M, Wragg D, Gauthier L, Vignal A, Neumann P, Neuditschko M. Using Whole-Genome Sequence Information to Foster Conservation Efforts for the European Dark Honey Bee, *Apis mellifera mellifera*. *Front Ecol Evol*. Frontiers, 2016;4. doi: 10.3389/fevo.2016.00140.

90. Groeneveld LF, Kirkerud LA, Dahle B, Sunding M, Flobakk M, Kjos M, et al.. Conservation of the dark bee (*Apis mellifera mellifera*): Estimating C-lineage introgression in Nordic breeding stocks. *Acta Agriculturae Scandinavica, Section A — Animal Science*. Taylor & Francis, 2020;69:157–68. doi: 10.1080/09064702.2020.1770327.

91. Jensen AB, Palmer KA, Boomsma JJ, Pedersen BV. Varying degrees of *Apis mellifera ligustica* introgression in protected populations of the black honeybee, *Apis mellifera mellifera*, in northwest Europe. *Molecular Ecology*. 2005;14:93–106. doi: 10.1111/j.1365-294X.2004.02399.x.

92. Muñoz I, De la Rúa P. Wide genetic diversity in Old World honey bees threaten by introgression. *Apidologie*. 2021;52:200–17. doi: 10.1007/s13592-020-00810-0.

93. Cánovas F, De la Rúa P, Serrano J, Galián J. Geographical patterns of mitochondrial DNA variation in *Apis mellifera iberiensis* (Hymenoptera: Apidae). *Journal of Zoological Systematics and Evolutionary Research*. 2008;46:24–30. doi: 10.1111/j.1439-0469.2007.00435.x.

94. Miguel I, Iriondo M, Garnery L, Sheppard WS, Estonba A. Gene flow within the M evolutionary lineage of *Apis mellifera*: role of the Pyrenees, isolation by distance and post-glacial re-colonization routes in the western Europe. *Apidologie*. 2007;38:141–55. doi: 10.1051/apido:2007007.

95. Diniz-Filho JA, Hepburn HR, Radloff S, Fuchs S. Spatial analysis of morphological variation in African honeybees (*Apis mellifera* L.) on a continental scale. *Apidologie*. 2000;31:191–204.

96. Hepburn HR, Radloff SE. Honeybees of Africa. Springer Science & Business Media, 2013.

97. Daly HV, Hoelmer K, Gambino P. Clinal geographic variation in feral honey bees in California, USA. *Apidologie*. 1991;22:591–609.

98. Oleksa A, Căuia E, Siceanu A, Puškadija Z, Kovačić M, Pinto MA, et al.. Supporting data for “Honey bee (*Apis mellifera*) wing images: a tool for identification and conservation.” *GigaScience Database*. 2023; doi: 10.5524/102360.

Figure legends

Fig. 1. Locations from which honey bee samples were collected. Jitter was used to show multiple samples from the same or similar location.

Fig. 2. Position of landmarks on the forewing of a honey bee worker. The landmarks were indicated with red dots. The blue circles around them should be tangent to the venation outline in three points.

Fig. 3. The first two principal components of wing shape (A). Ellipses indicate 95% confidence regions assuming multivariate t-distribution. The two bottom wireframe graphs illustrate change of wing shape along the first (B) and the second (C) principal component. The blue and red lines and dots indicate samples with minimum and maximum values, respectively.

Fig. 4. First (A) and second (B) principal component interpolated over sampling locations using a generalised additive model.

Fig. 5. Discrimination between countries based on the first two canonical variates. Ellipses indicate 95% confidence regions assuming multivariate t-distribution.

Fig. 6. UPGMA tree illustrating similarities between the shape of the wings collected from different countries.

Fig. 7. Relationship between geographical distance and Mahalanobis distance among countries.

Fig. 8. Mahalanobis distance to lineage A (A), C (B), M (C), and O (D) interpolated over sampling locations using a generalised additive model.

Supplementary figure legends

Fig. S1. Projection of the samples from different countries to canonical variate space of honey bee lineages published in Nawrocka et al. [45]. Ellipses indicate 95% confidence regions assuming multivariate t-distribution. Black ellipses indicated lineages, other ellipses indicate countries.

Fig. S2. Samples from different countries classified as honey bee lineages. Jitter was used to show multiple samples from the same or similar location.

Figure 1

Click here to access/download;Figure;fig1.pdf

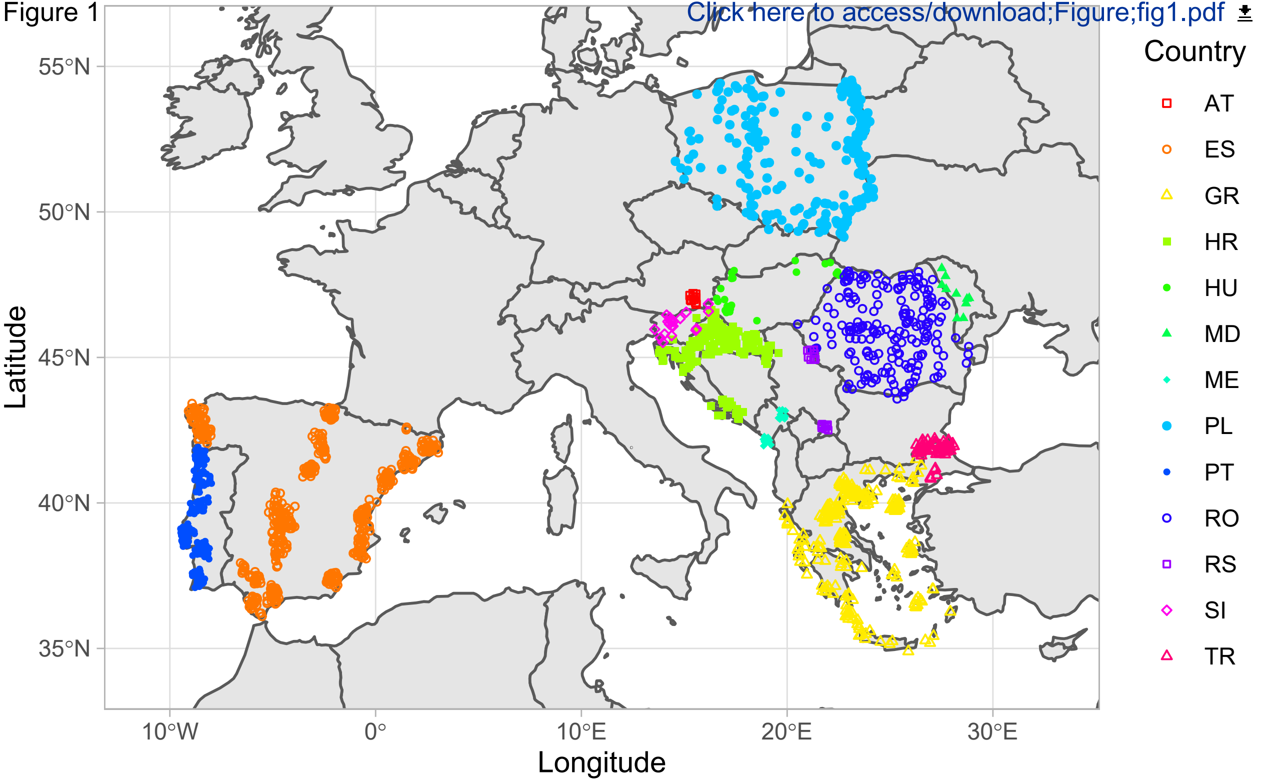

Figure 2

Click here to access/download;Figure;fig2.pdf

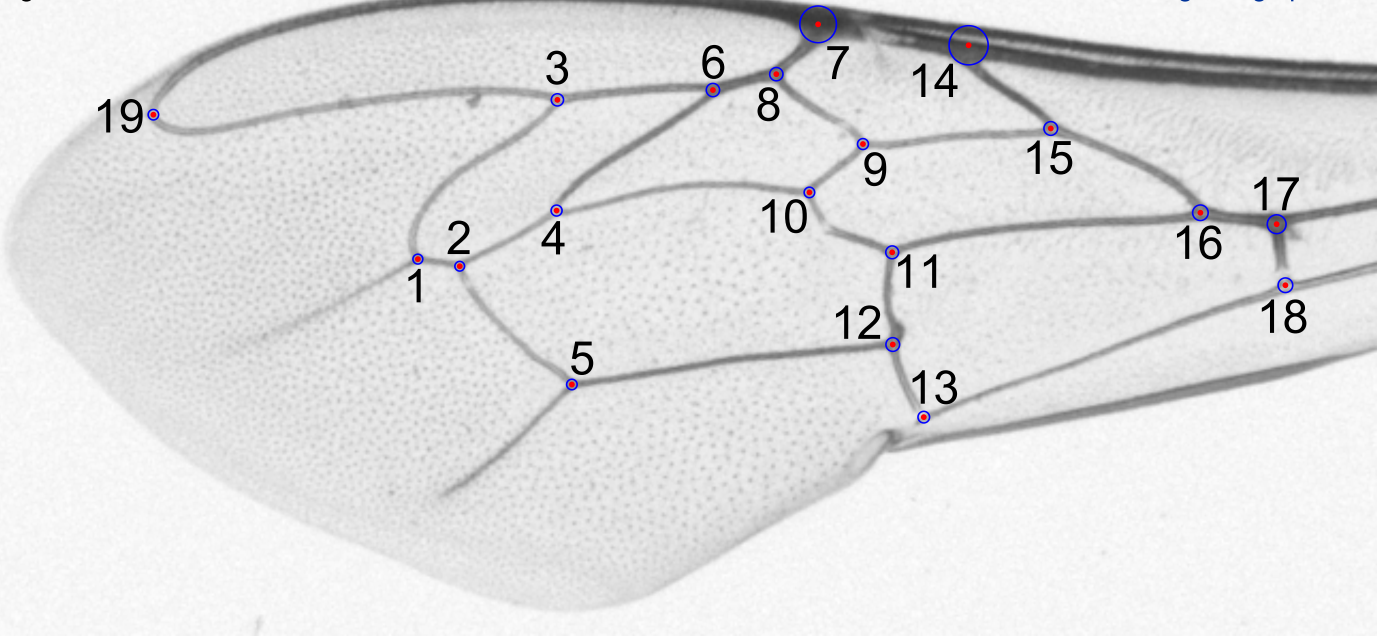

Figure 3

Click here to access/download;Figure;fig3.pdf

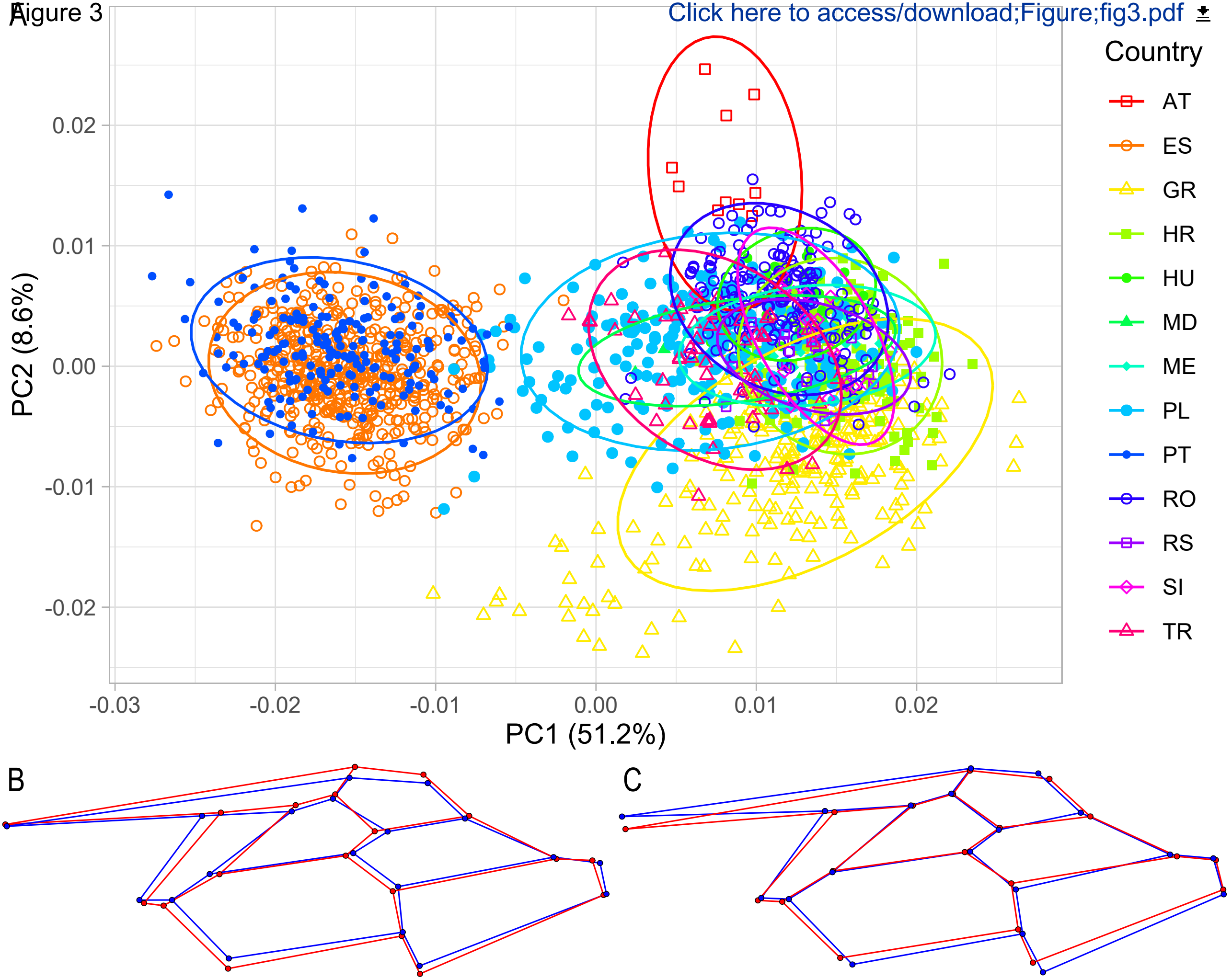

Figure 4

Click here to access/download;Figure;fig4.pdf

A

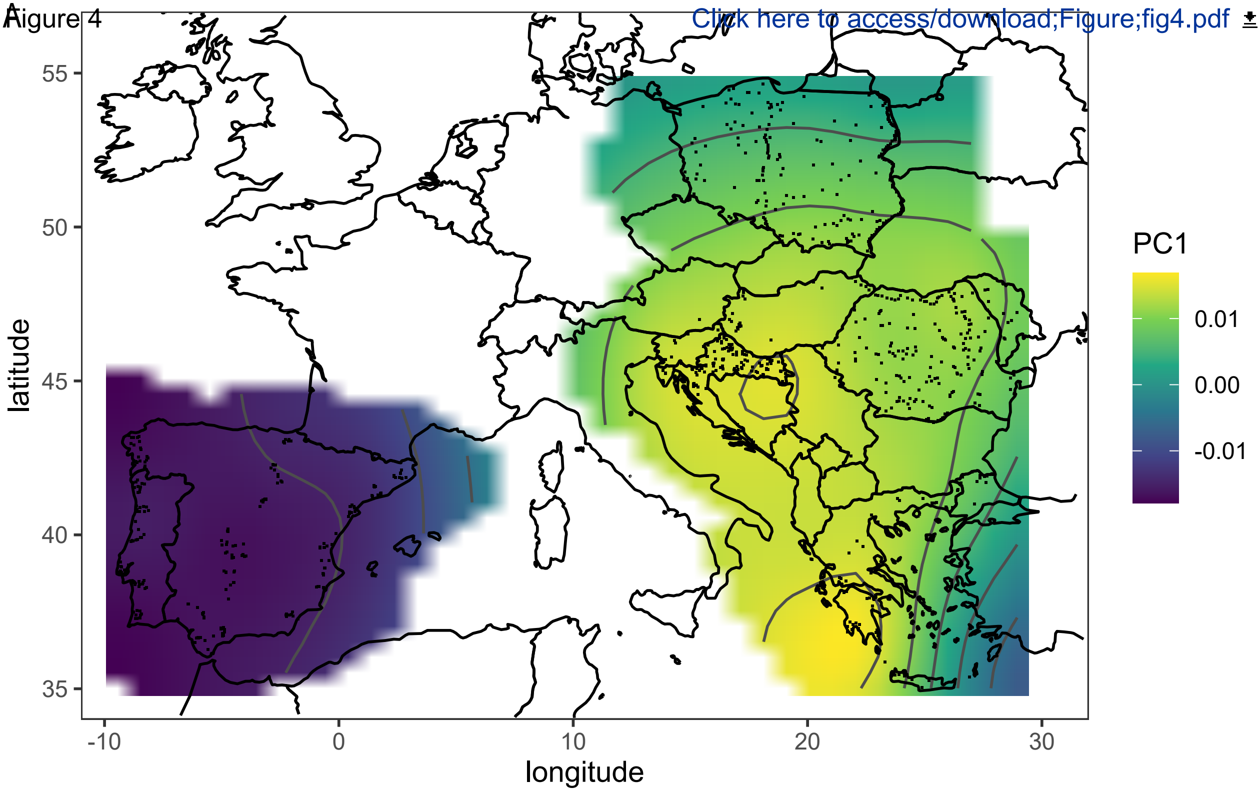

B

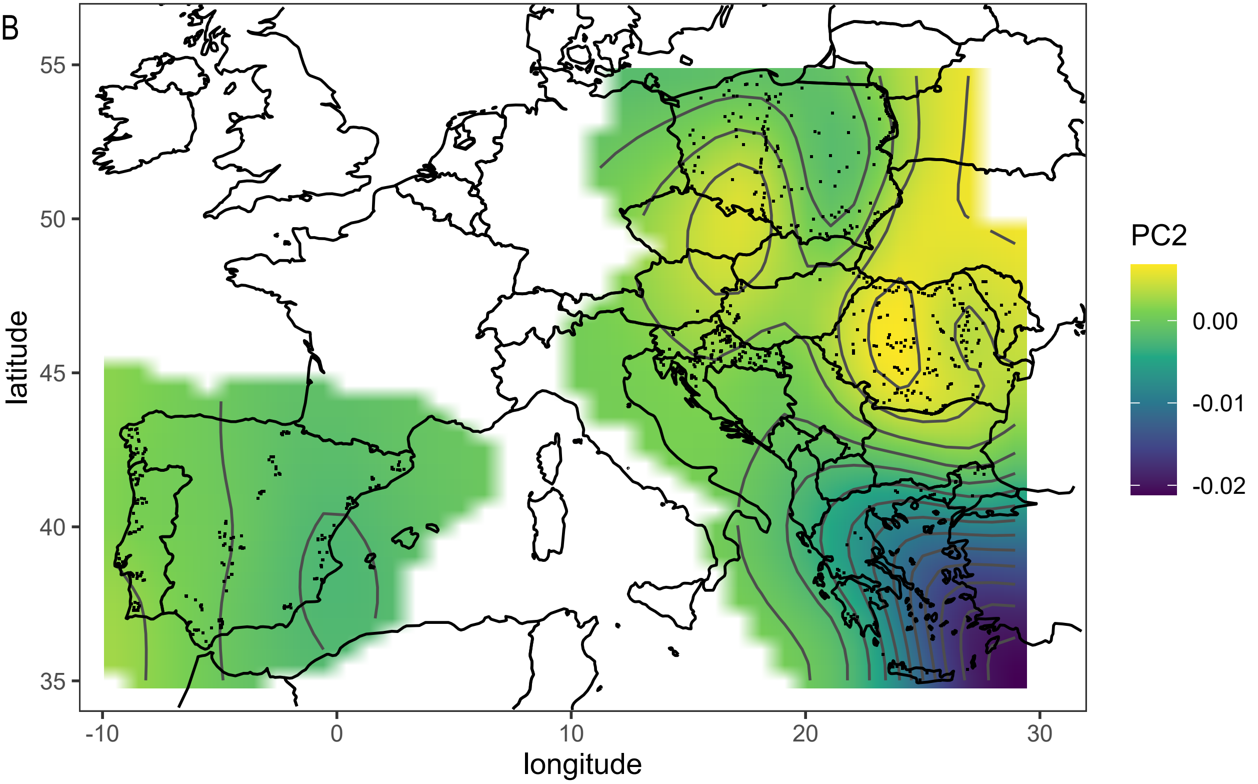

Figure 5

Click here to access/download;Figure;fig5.pdf

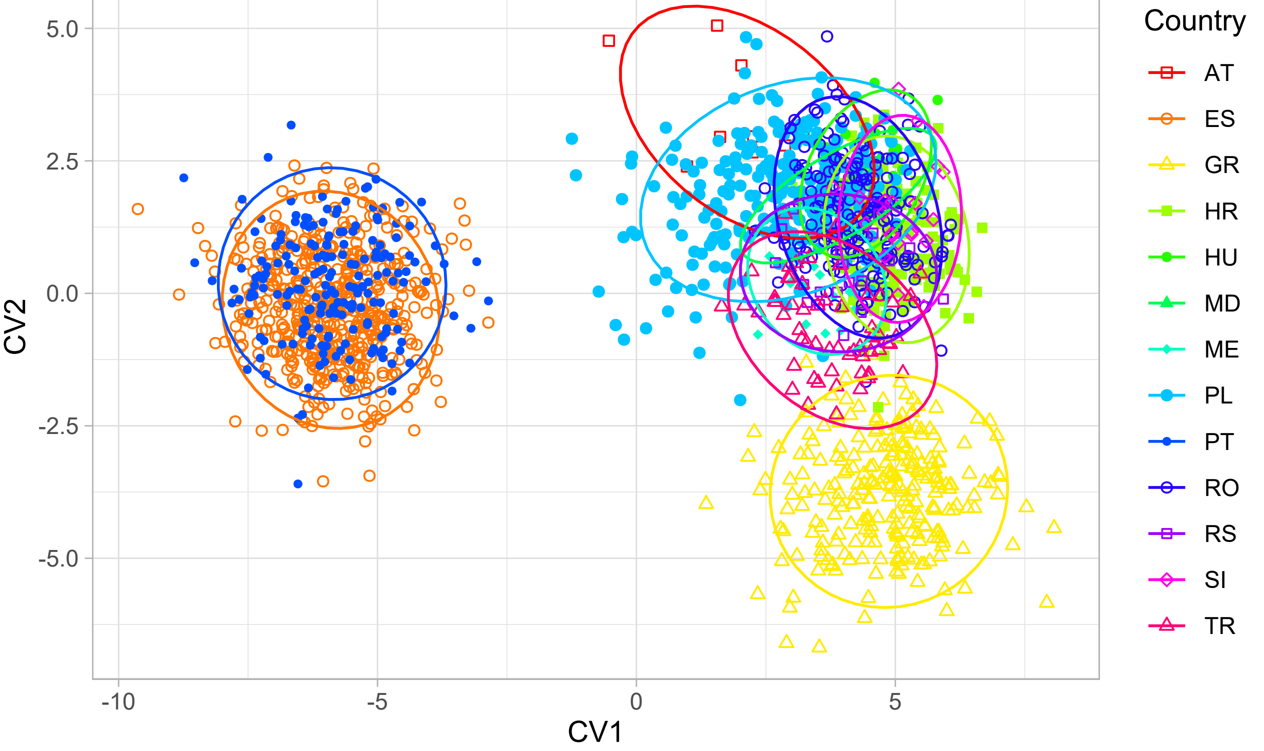

Figure 6

Click here to access/download;Figure;fig6.pdf

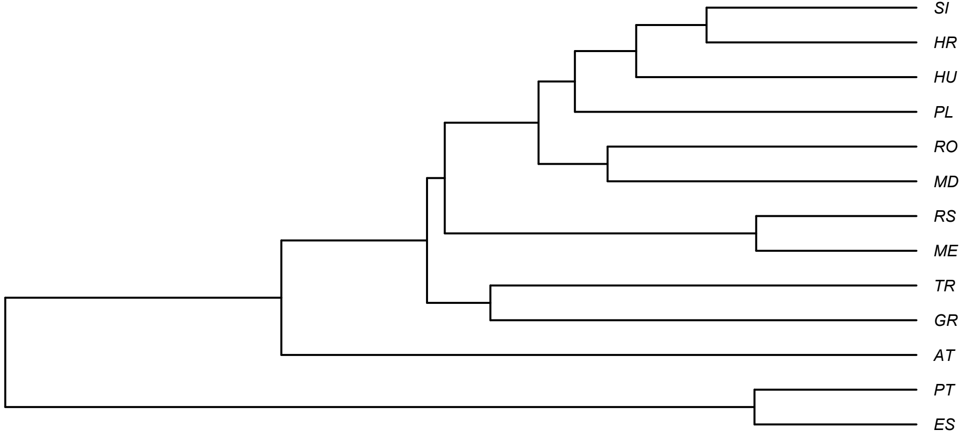

Figure 7

Click here to access/download;Figure;fig7.pdf

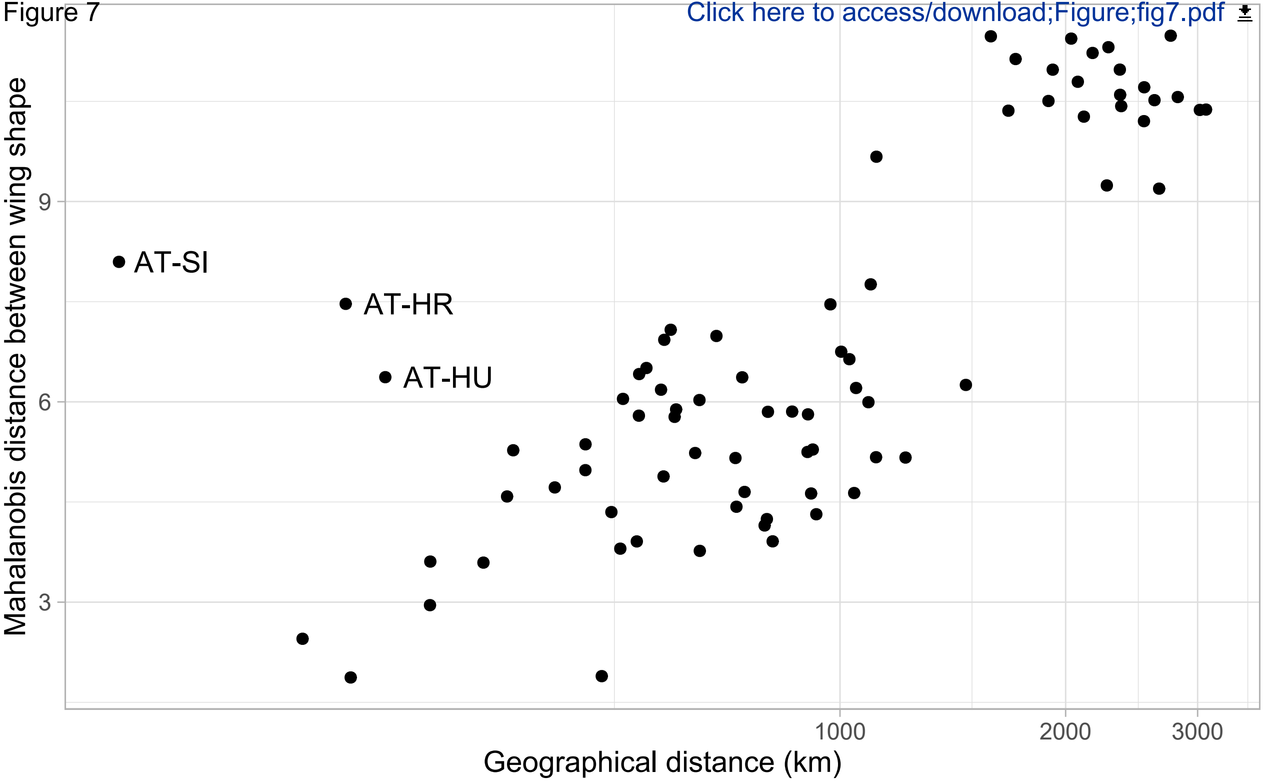

Figure 8

Click here to access/download;Figure;fig8.pdf

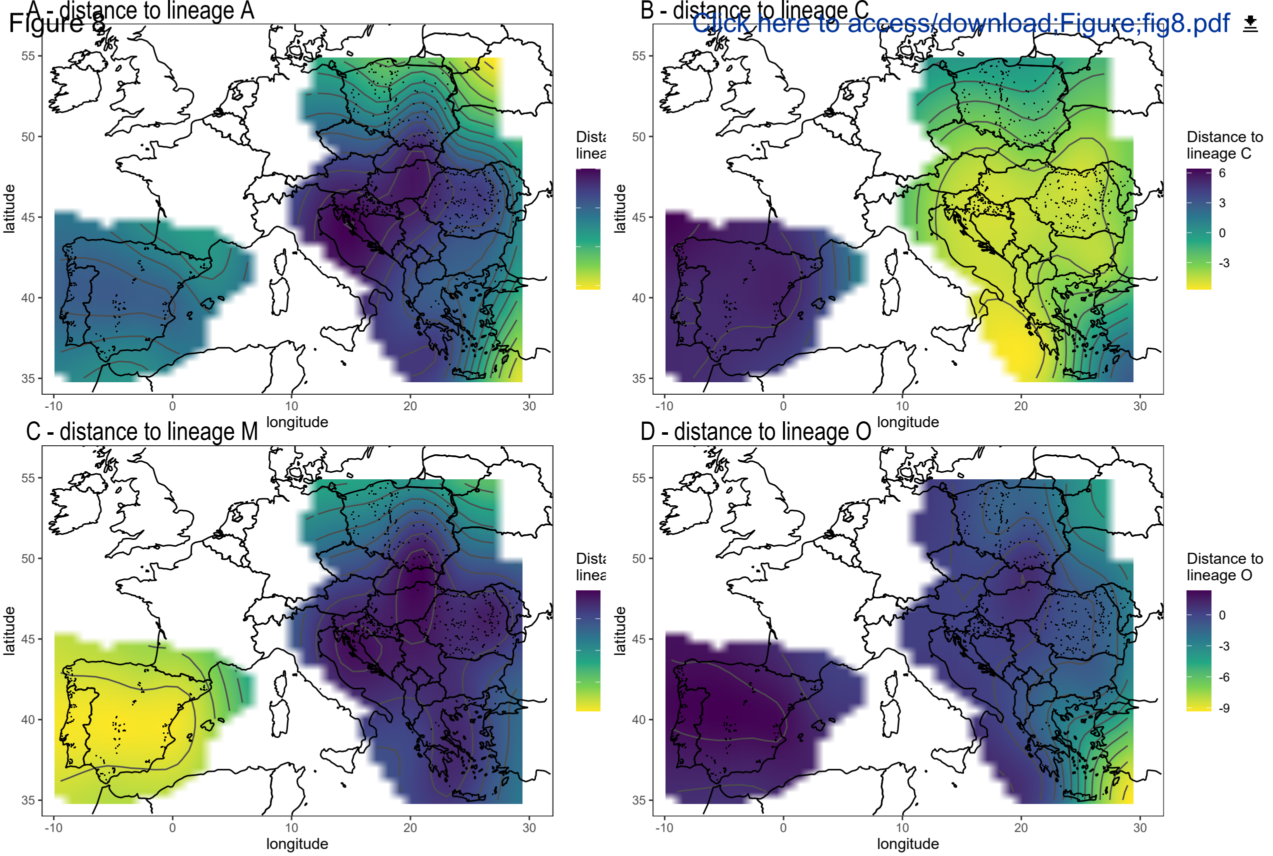

Supplementary figure 1

Click here to access/download;Figure;figS1.pdf

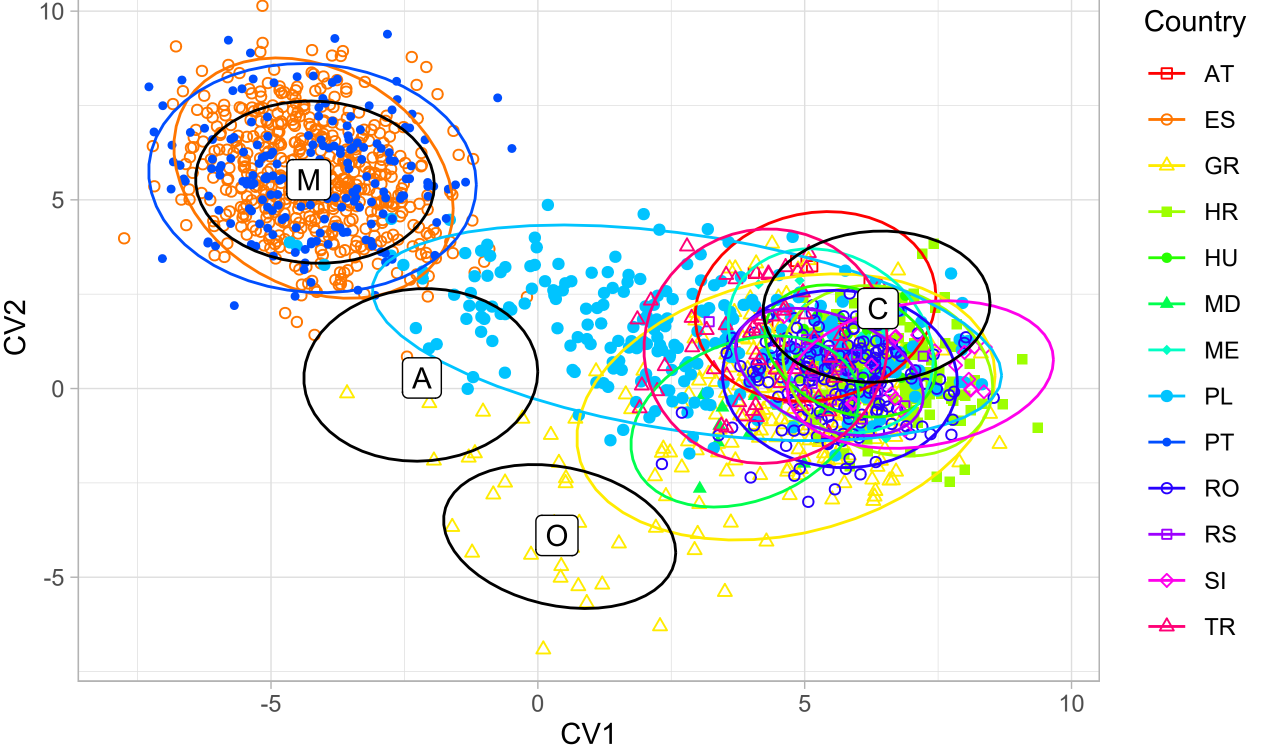

Supplementary figure 2

Click here to access/download;Figure;figS2.pdf

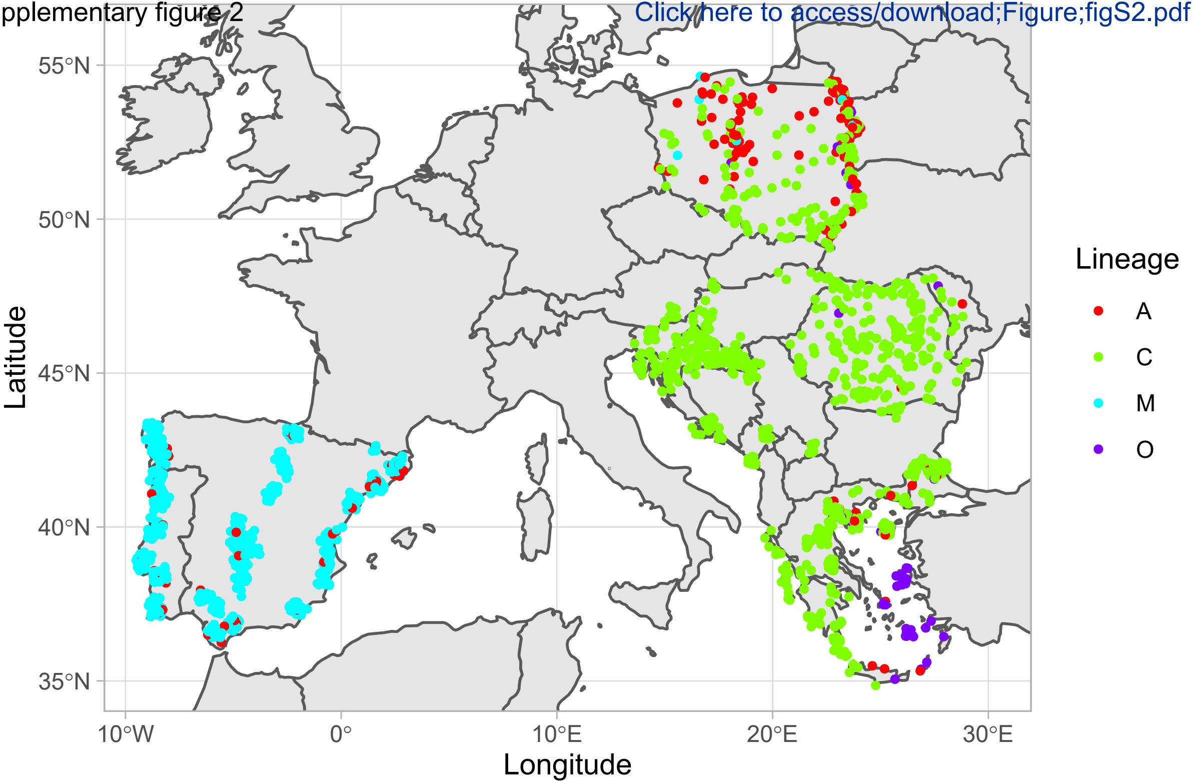

Supplementary document 1

Click here to access/download
Supplementary Material
Apis-mellifera-wings-EU.html
