# Supplementary figures and images for: Honey bee (Apis mellifera) wing images: a tool for identification and conservation
Source: Gigascience. 2023 Mar 27;12:giad019. doi: 10.1093/gigascience/giad019 (PMC10041535; doi:10.1093/gigascience/giad019)

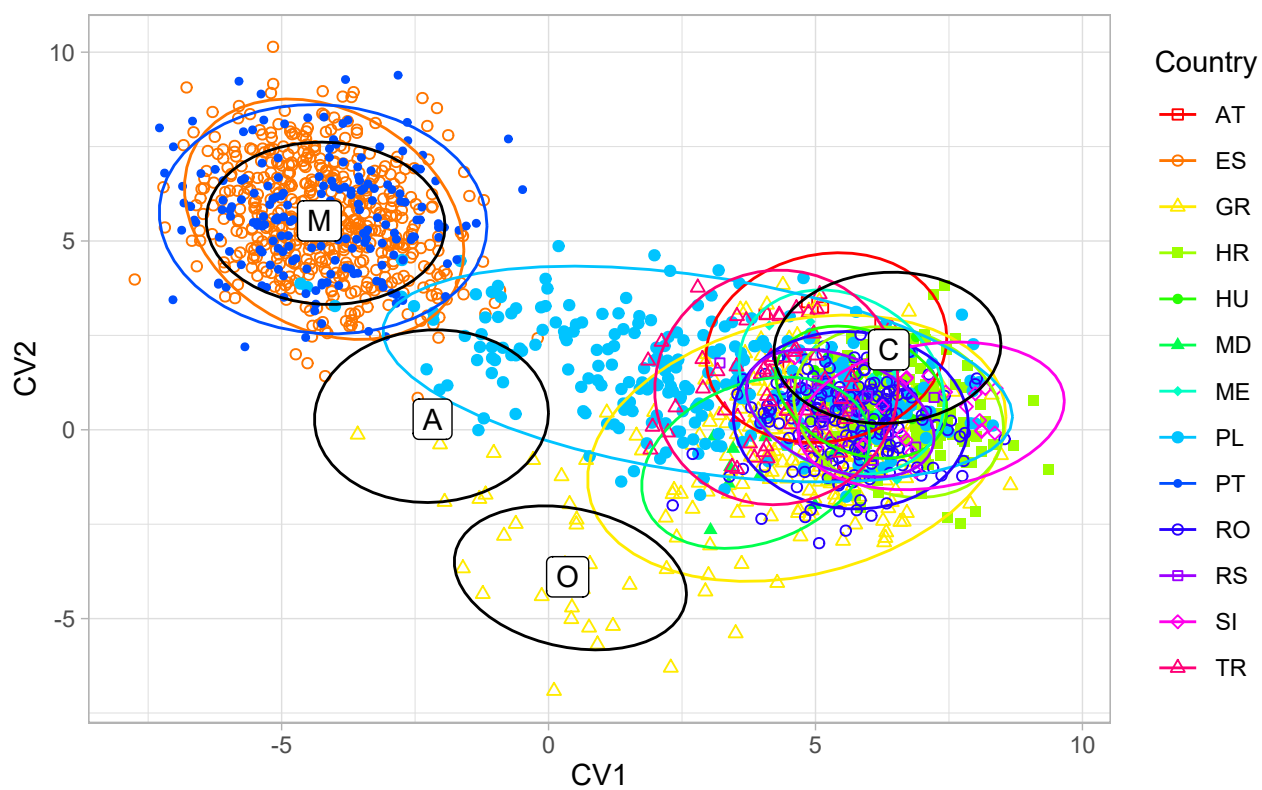

Supplement: giad019_Supplemental_Figures [file giad019_supplemental_figures.zip › figS1.pdf]

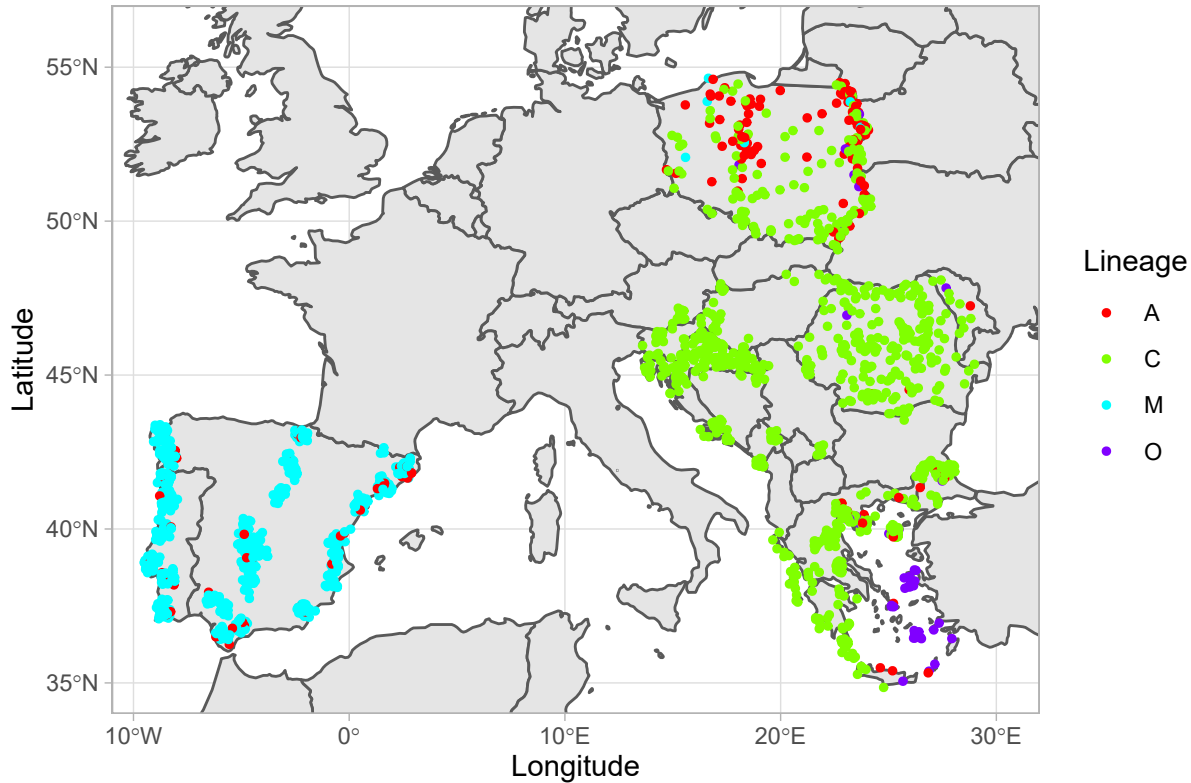

Supplement: giad019_Supplemental_Figures [file giad019_supplemental_figures.zip › figS2.pdf]
