# Supplementary material for: Honey bee (Apis mellifera) wing images: a tool for identification and conservation
Source: Gigascience. 2023 Mar 27;12:giad019. doi: 10.1093/gigascience/giad019 (PMC10041535; doi:10.1093/gigascience/giad019)
Supplement: giad019_Response_to_Reviewer_Comments_Original_Submission [file giad019_response_to_reviewer_comments_original_submission.pdf]

Author's Response To Reviewer Comments

Close

Dear Hongling Zhou,
Please find attached the revised manuscript entitled “Honey bee (Apis mellifera) wing images: a tool for identification and conservation” (manuscript number: GIGA-D-22-00297), which we hope you will find suitable for publication in GigaScience as Data Note.
We are grateful to both Reviewers for their constructive comments and corrections. We have revised the manuscript according to most of them. In addition, we made corrections to the English across the manuscript to improve grammar and its readability. We believe that the changes have improved the manuscript substantially.
Please find below our point-by-point responses to the Reviewer, which we indicate with subheadings “Response:”.
Yours sincerely,
Adam Tofilski

Reviewer #1: This exceptional Data Note reports on honey bee (Apis mellifera) wing images, and showcases how analysis of wing venation patterns can be used to aid honey bee conservation. The authors highlight that "a crucial step for the protection of honey bee biodiversity is reliable differentiation between native and non-native bees" and towards this end provide 26,481 honey bee wing images representing 1,725 samples from 13 European countries. The supporting dataset is archived in Zenodo and the images are of exceptional quality and have very high contrast.

In their analysis, the authors segment the wing venation patterns and use image analysis to delineate branch points. These branch points are anatomical landmark points that are then used in a morphometric analysis to identify honey bee subspecies. The wing venation analysis approach utilised in this Data Note has been published previously [Nawrocka et al. Computer software for identification of honey bee subspecies and evolutionary lineages. Apidologie 49, 172-184 (2018). https://doi.org/10.1007/s13592-017-0538-y]. The position of all 19 landmarks in honey bee wing is is conveniently summarised in the following figure from the original Nawrocka et al (2018) paper [see https://link.springer.com/article/10.1007/s13592-017-0538-y/figures/1].

An important feature of this Data Note is that the authors provide details of geographic (latitude / longitude) coordinates for all honey bees included in the study. Principal Component Analysis of geographic coordinates and honey bee wing landmark coordinates is used to describe how the wing shape varies geographically. The R scripts used in this study are very nicely documented in a supporting HTML file [see Document-1-supplementary-material.html]. As a use case example, the authors showcase how this approach can be used to identify the origin of an unknown sample.

The supporting data, including all wing images, are archived in Zenodo where they have been ascribed a Public Domain Dedication (CC0). The Zenodo repository includes the following:

* 26,481 honey bee wing images representing 1,725 samples from 13 European countries
* Landmark coordinates of wing venation patterns
* Geographic coordinates of honey bee samples
* Map visualisation of geographic coordinates of honey bee samples

For a subset of the image data, additional details of image resolution (pixels per metre) are provided. From a reuse perspective, this is a significant addition as it enables more traditional morphometric analysis (length, width, etc) to be performed on this subset of image data.
Response:
Thank you for summarising our manuscript with some positive notes which are very encouraging to us.

Minor point

I attempted to run the supporting R scripts on MacOS. Whereas many of the R scripts worked, some of the supporting libraries could not be installed and so, for some of the scripts, I was unable tor reproduce the results. This could be due to the version of R (version 3.5.2) that I was using. To illustrate the problem, I attach a screen capture of the issue I experienced when attempting to install the package "phangorn".
Response:
In order to reproduce our results, users need to use a recent version of R (v. 4.0.3). In particular, the package phangorn requires R in a version later than 3.5.2. The first two screenshots with information about problems were probably done before a R upgrade. The last screenshots show the R version 4.2.2; at this stage, package phangorn should work without problem, as long as all its dependencies listed at the bottom of the last screenshots are properly installed.

Towards this end, I invite the authors to detail any additional requirements (e.g. platform dependencies, version of R) that are required to reproduce the analysis showcased in the manuscript. These details should be included in a section of the manuscript entitled "Availability of source code and requirements" as described in the GigaScience Data Note instructions (see https://academic.oup.com/gigascience/pages/data_note), and should include the following:

* Project name: e.g. My bioinformatics project
* Project home page: e.g. http://sourceforge.net/projects/mged
* Operating system(s): e.g. Platform independent
* Programming language: e.g. Java
* Other requirements: e.g. Java 1.3.1 or higher, Tomcat 4.0 or higher
* License: e.g. GNU GPL, FreeBSD etc.
* RRID: if applicable, e.g. RRID: SCR_014986

Response:
In the lines 474-483, we added a section "Availability of source code and requirements" with the relevant information. More detailed information about R and packages can be found at the end of Supplementary Document 1 in section "Information about session". We added information about this project to: bio.tools, and workflowhub.eu.
We attempted to add RRID but our submission to scicrunch.org was rejected because our dataset is not “biomedical” and is not related to “antibodies, organisms, chemicals, plasmids or cell lines”

Reviewer #2: information copied from the attached PDF
In this manuscript, Oleksa et al. present a dataset of > 20.000 honey bee wings images. Each image is accompanied with shape descriptors (19 landmarks) and geographic sampling location. In addition, the authors provide an R script aiming at retrieving the shape and geographic descriptors, allowing to perform multivariate analyses in order (mainly) to: 1/ test whether wing shape descriptors allow to discriminate specimens according to their geographical origin. 2/ assign an unknown sample to a geographical location / evolutionary lineage. The R script allows also to run some other analyses. I think this is a valuable dataset. The R pipeline provided by the authors works smoothly and their analysis pipeline is accurate. Below are some suggestions that could help in improving the manuscript.
Response:
Thank you for your positive comments.

General comment:
According to the abstract, a major objective of the study is to provide a reference dataset that can be used to classify an “unknown sample”. However, this aspect is not given enough importance in the analysis. The coordinates of an “unknown sample” are given as supplementary material but I have not found the analysis of this “unknown sample” in the main text of the manuscript (although such analysis is given in the R script). I recommend to the authors to dedicate a section to this in the “results” section of the manuscript, to show the reader how good is the approach to assign a location to an “unknown sample”.
To make this point stronger, I would appreciate if independent samples from different locations/evolutionary lineages are tested to further investigate how good is the approach to correctly

assign a geographic location and/or evolutionary lineage to a given sample. By doing this, the authors could provide clear guidelines about the conditions for which the method “works” (i.e., correctly assign an unknown sample to a geographic location/evolutionary lineage). For example, from the data it seems clear that the method is good in discriminating Spain/Portugal, Greece and Turkey groups (Figure 2) but that an unknown sample coming from any other of the studied countries would be hardly identified. Is this actually the case? I suggest the authors to be clearer about the conditions under which the identification of an unknown sample is expected to work versus conditions under which the proposed method is likely to give erroneous predictions.
Response:
In lines 292-330, we added the new section "Identification of unknown samples". In this new section, there is information about accuracy of the identification. Apart from leave-one-out cross-validation, we used as unknown samples data from Nawrocka et al. (2018), which were recently uploaded to Zenodo: https://doi.org/10.5281/zenodo.7567336.
In lines 390-432, we added discussion related to this problem.

Other comments:
1/ R script
I have run all the R script. It is tidy, well annotated, and easy to follow. It works very well except in three following cases that need to be corrected:
sample.error <- sample.class[sample.class$error == 'error',
should be
sample.error <- sample.class[sample.class$error == "err\nor"
geo.dist <- distm(country.geo.data[c('longitude','latitude')], fun = distGeo)
should be
geo.dist <- distm(country.geo.data[c('longitude\n','latitude')], fun = distGeo)
Response:
The corrections are not clear to us. The first one can be related to the use of single or double quote. The two ways to delimit characters can be used interchangeably. We have changed all single quotes to double quotes.
In both corrections, the character \n was added. This was probably by accident because when this character is added the script does not work anymore.

Suuplementary file “unknown wings”: Remove all the “” from the dataset as it creates errors upon file importation in R.
Response:
The supplementary file “unknown.csv” was removed from the revised version. In the current version all csv files are without quotation marks.

2/ ‘Introduction’ and ‘Discussion’ sections need some rewriting to focus on the points to which the dataset is designed for. Sometimes I felt the reader get lost in details which are not so relevant.
Response:
We have removed from the discussion the parts that we feel are not essential.
We have added subheadings.

3/ Methods :
*It will help if the authors provide more details about the sampling conditions:
- How were bees captured ?
- How were individuals conserved before their wing was imaged ?
- How were wings mounted and imaged ? Provide imaging conditions + mounting procedure.
Response:
In lines 167-170 and 189-192, we have added more information about sample collection, wing mounting, and image acquisition. The samples originated from different studies and differed to some degree in terms of the methodology used. This is one of the factors that reduces repeatability. However, in the future, the image acquisition methodology for unknown samples will also vary between studies.

*Could the authors provide an actual picture of a wing with the landmarks positions (ref 43 only provides a scheme) ?

Response:
We added Fig. 2 with landmarks marked on the wing image.

*Line 228 : define CVA
Response:
We defined CVA in line 220.

*Line 228 : clarify what is meant by “appropriate transformation”
Response:
We have removed the expression “appropriate transformation” as it is not necessary.

4/ Results :
* If wing shape changes are noticeable, could the authors provide a figure showing how is wing shape varying geographically and/or between evolutionary lineages ? For example, in figure 2, what is the change in landmarks positions from a wing having low values along PC1 (e.g., Spain/Portugal), compared to a wing having high values along PC1 ? (e.g., Greece). Same for PC2. Alternatively, if relevant, this information could be provided for CV1 and CV2 in Figure 4. Practically speaking, this can be obtained with the function plotRefToTarget in R.
Response:
In Fig. 3, we added wireframes graphs (Fig. 3B, 3C), which indicate the shape change along PC1 and PC2. The wireframes graphs in case of CV1 and CV2 would be very similar to those representing PC1 and PC2, therefore, they were not added.

* Visualisation of lineages : I believe the plots obtained from the R code (at section “# Projection of the samples to canonical variate space from Nawrocka et al. 2018” and at the section “Classification of the samples to lineages according to Nawrocka et al. 2018”) are complementary to what is shown in figure 7 of the manuscript to visualise the relationship between the geographical variation and the evolutionary lineages. I suggest these plots to be included as main or supplementary figure.
Response:
We added Figs. S1 and S2. Because there is already 8 figures in the main text, we added them to the supplementary materials.

*Figures 2 and 4. Do ellipse represent 95% confidence interval of the mean ? If so specify in figure legend.
Response:
We added the information about the ellipses in figure captions (lines 818 and 828).

*Line 276 : Since using less than 25 wings/sample leads to misclassifications, should it be a recommendation to use at least 25 wings/sample ? Would “unknown samples “containing less than 25 wings be misclassified ? This technical aspect might be worth mentioning in the discussion.
Response:
We explained that the identification can be based on one sample, as long as it consists of at least 10 wings (lines 433-438).

Close
