# Supplementary material for: Honey bee (Apis mellifera) wing images: a tool for identification and conservation
Source: Gigascience. 2023 Mar 27;12:giad019. doi: 10.1093/gigascience/giad019 (PMC10041535; doi:10.1093/gigascience/giad019)
Supplement: giad019_Reviewer_1_Report_Original_Submission — Chris Armit -- 11/17/2022 Reviewed [file giad019_reviewer_1_report_original_submission.pdf]

Reviewer Report

Title: Honey bee (Apis mellifera) wing images: a tool for identification and conservation

Version: Original Submission **Date:** 11/17/2022

Reviewer name: Chris Armit

Reviewer Comments to Author:

This exceptional Data Note reports on honey bee (Apis mellifera) wing images, and showcases how analysis of wing venation patterns can be used to aid honey bee conservation. The authors highlight that "a crucial step for the protection of honey bee biodiversity is reliable differentiation between native and non-native bees" and towards this end provide 26,481 honey bee wing images representing 1,725 samples from 13 European countries. The supporting dataset is archived in Zenodo and the images are of exceptional quality and have very high contrast.In their analysis, the authors segment the wing venation patterns and use image analysis to delineate branch points. These branch points are anatomical landmark points that are then used in a morphometric analysis to identify honey bee subspecies. The wing venation analysis approach utilised in this Data Note has been published previously [Nawrocka et al. Computer software for identification of honey bee subspecies and evolutionary lineages. Apidologie 49, 172-184 (2018). https://doi.org/10.1007/s13592-017-0538-y]. The position of all 19 landmarks in honey bee wing is is conveniently summarised in the following figure from the original Nawrocka et al (2018) paper [see https://link.springer.com/article/10.1007/s13592-017-0538-y/figures/1].An important feature of this Data Note is that the authors provide details of geographic (latitude / longitude) coordinates for all honey bees included in the study. Principal Component Analysis of geographic coordinates and honey bee wing landmark coordinates is used to describe how the wing shape varies geographically. The R scripts used in this study are very nicely documented in a supporting HTML file [see Document-1-supplementary-material.html]. As a use case example, the authors showcase how this approach can be used to identify the origin of an unknown sample.The supporting data, including all wing images, are archived in Zenodo where they have been ascribed a Public Domain Dedication (CC0). The Zenodo repository includes the following:* 26,481 honey bee wing images representing 1,725 samples from 13 European countries* Landmark coordinates of wing venation patterns* Geographic coordinates of honey bee samples* Map visualisation of geographic coordinates of honey bee samplesFor a subset of the image data, additional details of image resolution (pixels per metre) are provided. From a reuse perspective, this is a significant addition as it enables more traditional morphometric analysis (length, width, etc) to be performed on this subset of image data.Minor pointI attempted to run the supporting R scripts on MacOS. Whereas many of the R scripts worked, some of the supporting libraries could not be installed and so, for some of the scripts, I was unable tor reproduce the results. This could be due to the version of R (version 3.5.2) that I was using. To illustrate the problem, I attach a screen capture of the issue I experienced when attempting to install the package "phangorn".Towards this end, I invite the authors to detail any additional requirements (e.g. platform dependencies, version of R) that are required to reproduce the analysis showcased in the manuscript. These details should be included in a section of the manuscript entitled

"Availability of source code and requirements" as described in the GigaScience Data Note instructions (see https://academic.oup.com/gigascience/pages/data_note), and should include the following:* Project name: e.g. My bioinformatics project* Project home page: e.g. http://sourceforge.net/projects/mged* Operating system(s): e.g. Platform independent* Programming language: e.g. Java* Other requirements: e.g. Java 1.3.1 or higher, Tomcat 4.0 or higher* License: e.g. GNU GPL, FreeBSD etc.* RRID: if applicable, e.g. RRID: SCR_014986

Level of Interest

Please indicate how interesting you found the manuscript: Choose an item.

Quality of Written English

Please indicate the quality of language in the manuscript: Choose an item.

Declaration of Competing Interests

Please complete a declaration of competing interests, considering the following questions:

- Have you in the past five years received reimbursements, fees, funding, or salary from an organisation that may in any way gain or lose financially from the publication of this manuscript, either now or in the future?
- Do you hold any stocks or shares in an organisation that may in any way gain or lose financially from the publication of this manuscript, either now or in the future?
- Do you hold or are you currently applying for any patents relating to the content of the manuscript?
- Have you received reimbursements, fees, funding, or salary from an organization that holds or has applied for patents relating to the content of the manuscript?
- Do you have any other financial competing interests?
- Do you have any non-financial competing interests in relation to this paper?

If you can answer no to all of the above, write 'I declare that I have no competing interests' below. If your reply is yes to any, please give details below.

I work for GigaScience where I perform checks on the quality of image data and the image analysis approach. My funding is not dependent on the outcome of this review, and I declare that I have no competing interests.

I agree to the open peer review policy of the journal. I understand that my name will be included on my report to the authors and, if the manuscript is accepted for publication, my named report including any attachments I upload will be posted on the website along with the authors' responses. I agree for my report to be made available under an Open Access Creative Commons CC-BY license

(http://creativecommons.org/licenses/by/4.0/). I understand that any comments which I do not wish to be included in my named report can be included as confidential comments to the editors, which will not be published.

Choose an item.

To further support our reviewers, we have joined with Publons, where you can gain additional credit to further highlight your hard work (see: https://publons.com/journal/530/gigascience). On publication of this paper, your review will be automatically added to Publons, you can then choose whether or not to claim your Publons credit. I understand this statement.

Yes Choose an item.
